# Study protocol for a randomized clinical trial evaluating the safety and efficacy of autologous adipose-derived stem cell therapy for ulcers in patients with critical limb ischemia

**DOI:** 10.1371/journal.pone.0318922

**Published:** 2025-04-09

**Authors:** Francisco José de Oliveira Filho, Lenize da Silva Rodrigues, Sidia Maria Baptista do Amaral, Pedro Luciano Mellucci Filho, Natália Bronzatto Medolago, Ana Lívia de Carvalho Bovolato, Rita de Cássia Alvarado, Matheus Bertanha

**Affiliations:** 1 Department of Surgery and Orthopedics, São Paulo State University – UNESP, Botucatu Medical School, Botucatu, São Paulo, Brazil; 2 Applied Biotechnology Laboratory, Research Nucleus of Clinical Hospital, São Paulo State University – UNESP, Botucatu Medical School, Botucatu, São Paulo, Brazil; 3 Clinical Hospital of the Botucatu Medical School, São Paulo State University – UNESP, Botucatu, São Paulo, Brazil; 4 Clinical Research Unit, São Paulo State University – UNESP, Botucatu Medical School, Botucatu, São Paulo, Brazil; PLOS: Public Library of Science, UNITED KINGDOM OF GREAT BRITAIN AND NORTHERN IRELAND

## Abstract

**Background:**

Peripheral artery disease (PAD) can develop into critical limb ischemia (CLI), which is characterized by resting pain at rest, ulcerations, or gangrene, with a high risk of amputation. The optimum course of treatment at this point is arterial revascularization, although this has a significant financial cost and is not always feasible or successful in reducing pain, healing ulcers, or preventing amputations. In situations where traditional alternatives for treating PAD have been exhausted, recent developments in cell therapy may offer a viable substitute.

**Objective:**

The purpose of this study is to assess the safety and effectiveness of using expanded autologous adipose-derived stem cells (ASCs) in cellular therapy for the treatment of PAD patients who developed chronic artery ulcers.

**Methods:**

An open randomized clinical trial will be carried out with two groups of twenty patients with CLI: In group 1, 2g of abdominal adipose tissue will be taken to produce ASCs. These cells will then be expanded in a lab (cell processing center) for 14–21 days before being applied to the lesion using bio-dressings and perilesional subcutaneous injections. Group 2 will receive conventional treatment with hydrogel-based dressing. There will be regular clinical assessments, supplementary tests, and photo documentation. The main efficacy outcome will be partial or complete healing of the wound. Safety outcomes will be monitored for infections, gangrene, amputations, and death. Participants will be monitored for 90 days. Cases of major amputation of the studied limb will not be included. The results will be evaluated by an independent external evaluator who is blind to the groups. Considering the high prevalence and socioeconomic consequences related to CLI and limb amputation, this study is expected to provide a positive social and financial impact on the Brazilian Unified Health System.

ClinicalTrials.gov: NCT06326203.

## Introduction

Peripheral arterial disease (PAD) is a clinical manifestation of atherosclerotic disease in arteries that supply the limbs [1,2]. PAD of the lower limbs affects more than 230 million adults worldwide [3,4] and is often associated with other cardiovascular diseases like obstructive atherosclerotic disease of cerebral and coronary vessels, consequently increasing the risk of stroke and myocardial infarction [5,6].

The most urgent form of PAD is critical limb ischemia (CLI) which occurs when the arterial perfusion is severely impaired, leading to intense pain and the occurrence or worsening of ulcers that may be difficult to heal [7,8]. CLI affects between 11% and 20% of patients with PAD [9], and in extreme cases, can lead to gangrene of the extremities, which correlates with high cumulative rates of amputation and death [10].

The treatment of PAD and CLI is complex and involves a combination of medical therapies, endovascular interventions, and, in some cases, surgical bypasses [11–13]. Amputation-free survival may be challenging. Repeated vascular interventions may be necessary to maintain limb viability, sometimes exhausting the surgeon’s repertoire. Furthermore, many patients do not have the minimum clinical conditions to undergo surgical procedures, with persisting ulcers that are difficult to heal, generally resulting from traumas or minor amputations. Ultimately, after exhaustion of therapeutic options, due to the worsening quality of life and resting pain associated with CLI, amputations end up being the only alternative.

Recently, stem cell therapy [14–16] for the treatment of CLI has been presented as a promising alternative to improve ulcer healing and promote the regeneration of ischemic tissues [17–21]. Preliminary studies indicate that the application of stem cells can increase blood perfusion and reduce amputations, offering new hope for these patients [22–24]. In a systematic review study by Jiang et al [25], it was concluded that autologous stem cell therapy is effective and safe for improving chronic ulcer healing of the lower limbs, without serious adverse effects.

Therefore, the objective of this study is to evaluate the safety and efficacy of cellular therapy with autologous adipose-derived stem cells (ASCs) using perilesional subcutaneous injections and bio-dressings [26] over ulcers for patients with PAD in advanced-stage CLI. The results will be compared with a control group that will receive conventional hydrogel dressings (Dersani Hydrogel, Megalabs Pharmaceutical S.A. Rio de Janeiro – RJ, Brazil, registration number 80219190002). Data from this study may corroborate previous literature and support the development of cell therapy to offer a new alternative for patients with PAD and CLI.

## Method

### Study design

This is a phase I/II, single-center, randomized clinical trial (RCT), PROBE (Prospective Randomized Open, Blinded Endpoint), controlled to evaluate the safety and efficacy of using ASCs to treat injuries in patients with PAD and advanced-stage CLI. The study was designed to attend the guidance Standard Protocol Items: Recommendations for Interventional Trials for clinical trial protocols (SPIRIT). (S1 File SPIRIT checklist)

### Study location

The study will be conducted at the Clinical Hospital of the Medical School (HC-FMB), and the Clinical Research Unit (UPECLIN) of São Paulo State University (Unesp), Botucatu Campus. The ASCs will be prepared at the Cell Processing Center (CPC) of the Applied Biotechnology Laboratory in the same institution.

### Study population

The target population will be participants of both sexes with advanced-stage PAD and CLI of the lower limbs, without the possibility of effective revascularization.

### Clinical trials

Information about the study and results will be published in international journals and registered on the United States National Institutes of Health website: www.clinicaltrials.gov, and the registered protocol is “Cell Therapy for Lower Limb Ulcers in Patients With Critical Limb Ischemia” number NCT06326203. This is the first version of the protocol and was registered in clinical trials on 03/16/2024. The last release was made public on 12/04/2024.

### Inclusion criteria

- Ability to understand and accept written free informed consent form (ICF) (S2 File ICF in Portuguese and English).
- Both sexes, of any ethnic origin.
- Age between 18 and 90 years old at the time of signing the ICF.
- Absence of distal pulses of the affected lower limb (anterior tibialis, dorsalis pedis, and posterior tibialis).
- PAD with CLI, classified as Fontaine IV [27] or Rutherford 5 and 6 [28].
- Between 1 and 3 ulcers on the foot or distal third of the leg of at least 1 cm^2^ up to a maximum of 20 cm^2^ in area.
- Previous treatment with conventional dressings, antibiotic therapy, or debridement (if necessary) and no improvement in a minimum period of 3 weeks.
- Having an ankle-brachial index (ABI) < 0.9 for below-knee arteries (anterior tibial, dorsalis pedis, posterior tibial, and fibular) or ABI > 1.3 in one or more of those arteries in association with type 2 diabetes (disease duration > 5 years) [29,30].
- Not undergoing revascularization treatment or failed revascularization treatment in the last 12 months.
- Impossibility of arterial revascularization of the affected limb and/or incomplete revascularization of that limb (subjected to endovascular or surgical treatment that was unable to reestablish blow-knee perfusion), meaning exhaustion of all surgical therapies.
- Optimized drug treatment for PAD and comorbidities.
- Availability to attend medical appointments.

After the participant’s inclusion in the study, regardless of the group allocated, they will be sent to collect laboratory tests: blood glucose, glycated hemoglobin, total cholesterol, high-density lipoprotein (HDL), low-density lipoprotein (LDL), triglycerides, complete blood count, uric acid, urea, creatinine, C-reactive protein (CRP), sodium, potassium, and immunomodulatory cytokine profile.

### Exclusion criteria

- Pregnancy planning, being pregnant, or in the postpartum period.
- Healed ulcer during the screening.
- Signs of systemic infection, active infection, devitalized tissue (necrosis) of the arterial ulcer, or infection of surgical prostheses. Participants excluded by these criteria may be reevaluated for inclusion after successful treatment with antibiotics and removal of the infectious focus (including minor amputations such as toes or forefoot).
- Active cancer, chemotherapy, radiotherapy, or cancer in remission for less than 6 months.
- Use of colchicine or immunomodulators.
- Infectious diseases such as Human Immunodeficiency Virus (HIV), Hepatitis B Virus (HBV), Hepatitis C Virus (HCV), and Human T-cell lymphotropic Virus (HTLV).
- Major amputation (at ankle, leg, or thigh level) of the limb to be studied.
- Diagnosed Coronavirus Disease (COVID-19) for less than 4 weeks.

### Participant replacement

Participants excluded during the screening period – which corresponds to the interval between the initial assessment and first reassessment of the arterial ulcer, before receiving cellular therapy – may be replaced by another participant. The new participant will be allocated to the same randomization position as the excluded participant to preserve the balance between treatment groups and the integrity of the randomization. The eligibility of substitutes will be verified according to the same inclusion and exclusion criteria established in the protocol.

**Fig 1:**
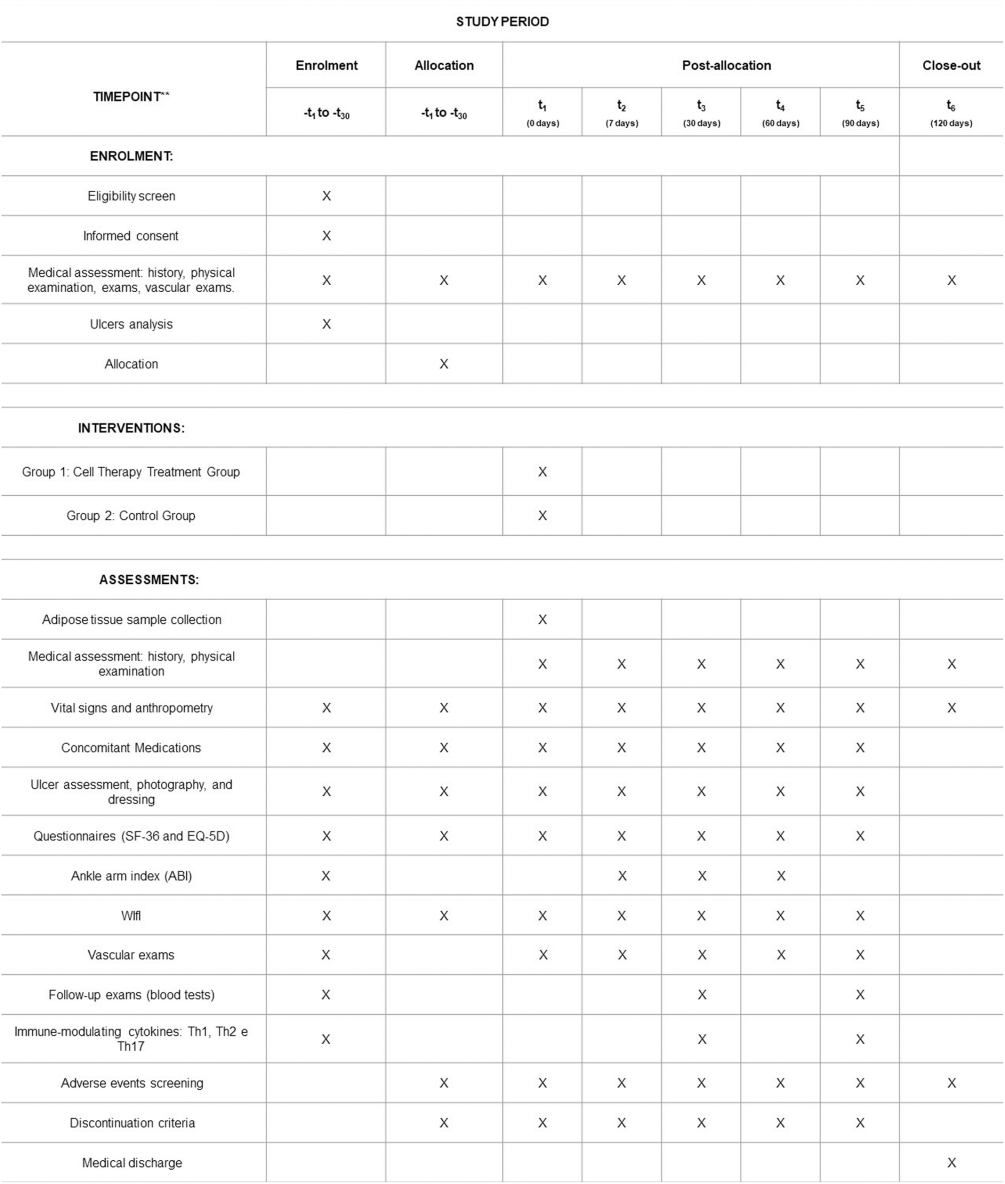
SPIRIT schedule of enrolment, interventions and assesments.

During this phase, participants with unstable clinical conditions will be monitored for up to one month. After this period, clinical reassessment will be carried out. If the underlying conditions are resolved, they may be reevaluated for inclusion (Fig 1).

### Sample size

The sample calculation considered two independent samples with an estimated proportion of the ulcer healing rate of 75% for group 1, which will be treated with cell therapy, and 30% for group 2, which will be treated with hydrogel dressing. Assuming a test power of 80% and a significance level of 5% for a two-sided test, the sample was defined as 20 participants for group 1 and 20 participants for group 2 (1:1 allocation). This study was designed to be analyzed with intention to treat (ITT).

### Randomization

Randomization will be prepared by a technician, using a random numerical table construction program available at http://stattrek.com/Tables/Random.aspx. Twenty positions will be drawn, with number 1 being fixed for Group 1 (cell therapy) and number 2 for Group 2 (control), randomly and consecutively. The envelope containing the randomization sequence will be held by an independent professional, the study team will not have access to the allocation order until time for adipose tissue collection.

#### Group 1.

Cell Therapy Treatment Group: Participants will receive expanded ASC treatment. The participant will undergo a surgical debridement procedure of the ulcer so that it is in its best condition (Visit 1). Subsequently, the participant will receive an ASC application via perilesional subcutaneous injections and a bio-dressing produced by the study team, also containing ASCs (the dressing containing ASC will remain in contact with the ulcer for 7 days in a single session). After removing the biological dressing, the participant will receive local care for the ulcer with a topical hydrogel dressing, dry gauze, and a crepe bandage, changed at least once a day.

#### Grupo 2.

Control Group: Participants will receive local ulcer care. The participant will undergo a surgical debridement procedure of the ulcer so that it is in its best condition (Visit 1). Subsequently, the participant will receive local care for the ulcer with a topical hydrogel dressing, dry gauze, and a crepe bandage, changed at least once a day.

### Recruitment

Participants will be recruited from June 1, 2025, to July 1, 2027. The data analysis should be finished by December 20, 2027. Eligible participants will be those who do not have an option for effective revascularization. They will be screened for inclusion in the study upon admission to the Clinical Hospital of the Faculty of Medicine, Botucatu, for treatment of peripheral artery disease (PAD) with chronic ulcers, or while receiving follow-up care at the Clinical Hospital Vascular Surgery’s Dressings Outpatient Clinic.

### Intervention

In the treatment group, the active component of this project’s cell therapy product is autologous ASCs [31–33], which will be expanded in the Laboratory (Cell Processing Center). The ASC will be made available to the patient in two presentations for use in a single treatment session: injectable solution and stable bio-dressing. The dosage planned for this clinical trial will be a single application of the products.

The procedure for applying the advanced cell therapy product proposed in this clinical trial will be carried out after locoregional (preferred) or general anesthesia, at the discretion of the anesthesiologist who will accompany the procedure, considering the participant’s clinical conditions, since the procedure cannot be performed without adequate analgesia. The application of injections and dressings will follow the rules of surgical procedures, carrying out antisepsis, placement of sterile drapes, manipulation of surgical materials, and opening of envelopes containing the products to be applied in a sterile manner.

Initial surgical cleaning and debridement will be carried out on the wound to make it ready to receive the treatment. Subcutaneous applications of 0.1 mL of the product consisting of the plasma solution and expanded autologous ASC will then be carried out every 1 cm from the edge of the ulcer (in the subcutaneous layer of the intact skin at the perimeter of the lesion), so that approximately 1x10^4^ ASCs are injected by puncture.

**Fig 2:**
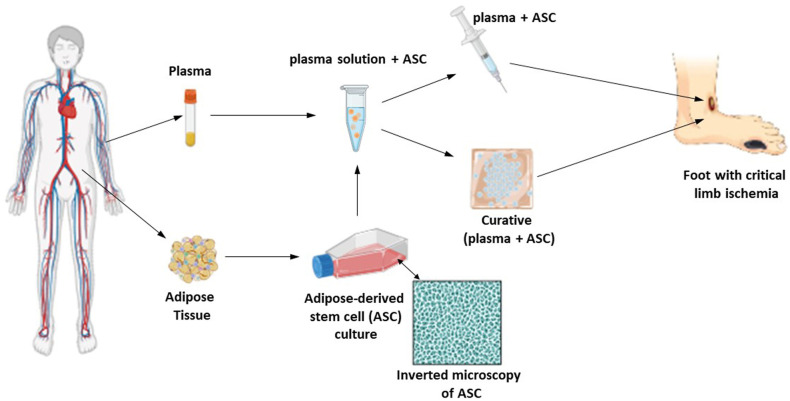
Schematic representation of patient treatment in Group 1. Collection of adipose tissue, ASC expansion, dilution of ASC in plasma, preparation of bio dressing and application to the patient.

After the injections, the topical application of the stable bio-dressing will be carried out containing the ASCs on a membrane FIBRACOL™ Plus (Systagenix Brazil, dressing containing 90% collagen and 10% calcium alginate) with the same plasma solution containing cells in the proportion of 1x10^4^ ASCs per cm^2^ of the ulcer area to be covered (Fig 2). The dressing will be covered with rayon gauze (identifying the end of the primary dressing), the secondary dressing will be composed of a thin layer of moistened gauze with 0.9% saline, a layer with dry gauze, and, finally, a crepe bandage. Only the secondary dressing will be changed daily by the participant or their caregivers, according to instructions given by the study team. After 7 days, the ASCs absorption phase will be completed and the participant will return for reassessment of the ulcer, moving on to the phase in which the dressing will contain only hydrogel.

### Discontinuation criteria – participant withdraw

The participant has the right to withdraw from the study at any time, either of their own will or by decision of the researcher, due to safety issues or poor adherence to follow-up. If a participant communicates their withdrawal from the study or revokes their consent, this communication will be documented by the researcher and no data will be collected.

If, upon withdrawal from the study, the participant has an ongoing adverse event or unresolved clinical or laboratory abnormality, the researcher will offer follow-up until the event is resolved or stabilized.

The study monitoring team will maintain regular contact with participants through telephone calls to encourage them to attend their medical reassessment appointments. Additionally, the research team will be available to address any questions or concerns from participants at any time.

### Discontinuation criteria – suspension of intervention

Participants will be withdrawn from the study if one of the following occurs:

- Major amputation (ankle, leg, or thigh) of the treated limb.
- Loss of follow-up.
- Clinical worsening (at the discretion of the investigator).
- Active ulcer infection.
- Unfit conditions to be subjected to the cellular therapy procedure within one month after the set date for the procedure.

If a participant’s intervention is discontinued, they will be followed up and encouraged to return for scheduled visits.

### Primary outcomes

#### Safety.

- Absence of major amputations (ankle, leg, or thigh).
- Efficacy
- Total ulcer epithelialization and limb salvage rate (non-occurrence of a major amputation).

### Secondary outcomes

#### Safety.

- Death, acute myocardial infarction (AMI), cerebrovascular accident (CVA), thromboembolic events such as deep vein thrombosis (DVT) and pulmonary embolism (PE), systemic infectious condition, cancer, local infections, bleeding, worsening pain and enlargement of the ulcer area.
- Any adverse event not mentioned in the previous items or negative and clinically significant changes in laboratory parameters (glycemia, glycated hemoglobin, HDL, LDL, total cholesterol, triglycerides, leukogram, hematocrit, uric acid, urea, creatinine, CRP, sodium, potassium, alkaline phosphatase (ALP), alanine transaminase (ALT), aspartate transferase (AST), gamma-glutamyl transferase (GGT), bilirubin, total protein, albumin, prothrombin time (PT), partial thromboplastin time (PTT)) and tissue histological analysis.

#### Efficacy.

- Improvement in limb perfusion [34]: Assessed by thermography [35,36] (significant increase in limb temperature > 0.3 ºC), vascular ultrasound with Doppler (flow wave characteristics and flow velocity incrementation), ABI [29,30] (increase in ABI value ≥ 0.15 for participants with ABI < 0.9), transcutaneous capnography [37] (incrementation ≥ 10% in O_2_ levels and/or decrease ≤ 10% in CO_2_ levels measured at the base of the hallux and the ankle, behind the medial malleolus) and WIfI (the Society for Vascular Surgery Lower Extremity Threatened Limb Classification System: risk stratification based on wound, ischemia, and foot infection) [38,39].
- Improvement in quality of life: assessed by the SF-36 scale (Medical Outcomes Study 36 – Item Short-Form Health Survey) [40].
- Reduction in pain: Assessed by the Visual Analogue Scale (VAS) [41] and reduction in the use of analgesics (quantitatively and qualitatively).
- Partial ulcer healing: Assessed by measuring epithelialization of the ulcer. The total ulcer area will be analyzed using the area measurement tool in the free software ImageJ™ [42,43].
- Improvement in health perception: Assessed by the EQ-5D scale by EuroQol Group [44].
- Clinical improvement: Observed through physiological (blood pressure) and laboratory parameters (glycemia, glycated hemoglobin, HDL, LDL, total cholesterol, triglycerides, leukogram, hematocrit, uric acid, urea, creatinine, CRP, sodium, potassium, alkaline phosphatase (ALP), alanine transaminase (ALT), aspartate transferase (AST), gamma-glutamyl transferase (GGT), bilirubin, total protein, albumin, prothrombin time (PT), partial thromboplastin time (PTT)).
- Formation of granulation tissue in the ulcer.
- Changes in the systemic inflammatory profile with a predominantly cellular immune response through analysis of immunomodulatory cytokines (Th1, Th2, and Th17) [45,46].

### Participant timeline

Follow-up of patients will continue for 120 days from the moment they receive treatment with the proposed advanced cell therapy. Time required for screening and preparations will not count toward the study follow-up period. Participants will be reassessed on days 7, 30, 60, 90, and 120 days after randomization. The closing assessment will be carried out after 120 days (assessment 6). After completing their participation in the study, the participants will be referred for routine follow-up at the vascular surgery outpatient clinic of the Clinical Hospital of the Faculty of Medicine of Botucatu. During this period, extra visits may occur, if necessary (S3 File Table 1 Assessment Schedule).

### Data collection plan

The study will be conducted at a single center, which will serve as the coordinating hub and be responsible for all stages of the RCT.

Verification and authenticity of the data quality entered into the electronic clinical record and printed documents will be carried out by the research team at our Clinical Research Unit (UPECLIN) according to the monitoring plan, during the execution and at the end of the clinical study.

To prevent data loss, we will develop standardized data collection forms and monitor the collected data regularly. Patients will be contacted frequently by telephone or social media, with updates often provided during these contacts. The appropriate clinical support will be offered to patients to minimize losses. Disease progression resulting in death or amputations will be defined as endpoints and will not be classified as data loss.

### Source documents

Source documents are original records that contain essential information for the reconstruction and evaluation of the clinical trial. They will be used to verify the accuracy and completeness of the data collected. The following principles will apply:

- Complete Records: All relevant data related to the study will be recorded completely and accurately in the source documents. This includes, but is not limited to, medical records, laboratory test results, scales, questionnaires, and ICF.
- Access and Storage: Source documents will be stored in a secure location with passcode-restricted access.
- Confidentiality: The confidentiality of participant data will be satisfied in all source documents. Identifiable information will be protected by data protection guidelines.
- Verification and Auditing: Source documents will be available for verification and inspection by ethical regulatory authorities if necessary.

### Data standards

Data collected from participants will be entered into the electronic case report form system (e-CRF) accurately and by the records in the source document. The study will be monitored regularly by qualified clinical monitors to verify adherence to the most current approved protocol, the accuracy of recorded data, the rights and safety of participants, and whether the study is being conducted by applicable regulatory requirements. Monitoring reports will be generated and reviewed periodically. Furthermore, inspections may be conducted by regulatory authorities or ethics committees to ensure compliance with legal and ethical requirements.

### Data protection

The privacy and confidentiality of participant data will be strictly protected throughout the study. Measures include:

- Confidentiality: All data collected will be treated as confidential. Only authorized members of the research team will have access to identifiable participant data.
- Anonymization: Participant data will be anonymized whenever possible, replacing identifiable information with unique codes. This will ensure that data cannot be directly linked to participants.
- Secure storage: Data will be stored in secure systems, protected by passwords and other cybersecurity measures. Physical access to data will be restricted to secure areas in the research center.
- Data Sharing: Any sharing of data with third parties will be carried out anonymously, with restricted purposes of scientific research and data analysis. Requests should be made directly to the main researcher, by email.
- Compliance with Regulations: The study will comply with all applicable data protection laws and regulations, including the Brazilian General Data Protection Law (LGPD).

### Blinding

Results will be anonymized, tabulated, and sent to evaluators and statisticians in a blind manner. They will be external members, not belonging to the study execution team, and will electronically receive the images of the ulcers identified only by the participant’s number and an electronic form with the variables already described for ulcer assessment.

### Sample size and Statistics

The results obtained through the primary and secondary outcomes will be tabulated weekly in an electronic spreadsheet by the study monitoring team. A sample of 40 participants is estimated. The data will initially be evaluated using descriptive statistics, with median/variance and/or mean/standard deviation analyses for quantitative variables and contingency table analyses for categorical/qualitative variables. To effectively recruit the intended number of participants, our strategy involves having study team members to actively engage with patients in the outpatient clinics and wards of HC-FMB. This direct approach will ensure that we connect with a wide range of patients who meet the criteria to participate in the study, maximizing our chances of success.

All data monitored in the study for Group 1 (cell therapy) and Group 2 (control) will be statistically compared. Quantitative data will be evaluated for their distribution (using the Shapiro-Wilk normality test) and compared using the T-test or the Wilcoxon test, depending on the type of distribution of the variables. For categorical variables, differences will be evaluated using Pearson’s Chi-square test or Fisher’s exact test. Bonferroni coefficient and multivariate analysis will also be used. In all statistical tests, the significance level of the independent variable will be set at 5% (p < 0.05).

Finally, the evaluation of the entire data set using multiple correspondence analysis (MCA) is proposed. For its application, each quantitative variable will be categorized according to the distribution quartiles of their respective data.

Categorical variables will be evaluated by means of Fisher’s exact test, continuous variables will be compared with non-parametric methods such as the Mann-Whitney test or U test, pairwise comparisons will be evaluated with the Wilcoxon test, multivariate analyses will be evaluated with regression analysis Cox curves, long-term clinical analyzes will be compiled into Kaplan Meir curves.

To prevent data loss, we will implement the measures previously outlined. However, if a category has missing data for 50% or more of the participants, that category will be removed from the analysis. In some cases, where only a portion of the data is missing, the missing data may not be included in the analysis. If more than 10% of the data is missing, we will consider using seasonal adjustment with linear interpolation, especially if the data shows trends and seasonal patterns. For data sets with a significant amount of missing data (while still less than 50%), multiple imputations will be employed.

### Adverse effects

Risks related to the use of autologous ASCs have not yet been described, considered non-existent for this study if they follow a rigorous cell production process. Treatment-related risks are those related to the surgical anesthetic procedure: surgical site infections, surgical wound dehiscence, worsening of pain in the treatment area, bleeding, gangrene, and cellulitis.

Partial data analysis will be conducted once at least five participants have been included in each group. The study will be halted if a statistically significant difference is observed in the primary or secondary safety outcomes for either group. In such a case, the Ethics and Research Committee/National Research Ethics Commission (CEP/CONEP) will be notified, and all necessary measures will be implemented.

### Expected results

This study aims to evaluate an innovative and advanced scientific therapy using autologous ASCs expanding in the laboratory as an alternative to prevent limb amputation for patients with peripheral artery disease (PAD). Cellular therapy could be an alternative to improve participants’ quality of life and overall health. The study results will also evaluate clinical changes such as wound healing, quality of life, blood tests, and additional examinations. These results will provide evidence regarding the effectiveness of the proposed treatment, within the study’s established timelines. By the end of the project, we hope to develop an advanced cell therapy protocol to help in this difficult clinical situation.

### Strengths and limitations

The strong point of the study is that it is a RCT for a disease stage that has practically no solution. Through the methodology employed, it will be possible to conclude the safety and efficacy of cell therapy for PAD plus critical ischemia and ulcers.

Limitations to this study include the impossibility of performing biopsies and tracking the applied stem cells.

### Ethical and regulatory considerations

The protocol, ICF, investigator’s brochure, and other pertinent documents were submitted and approved by the Plataforma Brasil system (https://plataformabrasil.saude.gov.br), the CEP/CONEP under the CAAE protocol 39873320.3.0000.5411, version 4, approved in 02/29/2024.(S4 File Protocolo Stem Cell in Portuguese and English)

Participants will be invited to join the study by a physician who is part of the study team, and they will sign an ICF (S2 File ICF in Portuguese and English). Each participant or their legal representative must complete two copies of the ICF, which will be dated and signed after all doubts have been clarified. Both copies will also require the signature of a witness and the researcher responsible for the RCT. One signed copy will be given to the participant, while the other will be securely archived in both physical and digital formats for a period of 10 years. Participants have the right to withdraw their consent to participate in the RCT at any time without facing any harm or embarrassment. There will be no form of remuneration provided to the participants. However, all care provided to participants during the study will be covered by the study sponsor.

Furthermore, the study will be conducted according to the approved protocol and applicable national and international ethical guidelines:

- Good Clinical Practice Guidelines
- Good Laboratory Practice Guidelines
- World Medical Association (WMA) Declaration of Helsinki

Any change to the protocol, consent form, or brochure will be sent to the Brazilian ethical and regulatory systems in the form of an amendment before being implemented in the study, except in cases where it is necessary to eliminate an immediate risk to participants.

### Data dissemination

The findings from this RCT, which investigates the safety and efficacy of utilizing adipose-derived stem cells (ASCs) for treating wounds in patients suffering from CLI and ischemic ulcers without a good option for treatment, will be published in peer-reviewed, open-access scientific journals. Furthermore, these results will be showcased at national and international scientific conferences and shared with the public to raise awareness. Participants will receive updates on their treatment outcomes in a comprehensive medical report at their final follow-up assessment. We prioritize protecting sensitive information; therefore, participants’ identities will remain confidential, and no identifying images will be disclosed. This study not only aims at the advance of cell therapy but also aspires to pave the way for broader clinical applications in treating CLI. In Brazil, we foresee a future where cell therapy is integrated into public health policies, supported by the Unified Health System (SUS). We are committed to ensuring that the results of this study are reliable and accessible to both the scientific community and the public. Our outreach efforts to disseminate and promote these findings will span a minimum of five years, with the potential to influence the scientific community and the broader public health landscape.

### Conclusion

This protocol describes in detail the planning and execution of the clinical trial aimed at offering an alternative clinical treatment for patients in an advanced stage of PAD who present critical ischemia of the lower limbs (CLI) and arterial ulcers who exhausted all possibilities of revascularization. Therefore, these are patients who have a high probability of limb amputation, and the proposed cell therapy may bring benefits. Few clinical studies with advanced cell therapy have been carried out in Brazil and only recently have we had a clearer regulatory framework in this regard. The results of this clinical trial have the potential to increase the range of treatments for patients with PAD and CLI. The findings could influence future research and clinical practices, promoting significant advances in cell therapy.

## Supporting information

S1 File SPIRIT checklist. (PDF)

S2 File ICF in Portuguese and English. (PDF)

S3 File Table 1 assessment schedule. (DOCX)

S4 File Protocolo stem cell in Portuguese and English. (PDF)
