# Supplementary material for: Study protocol for a randomized clinical trial evaluating the safety and efficacy of autologous adipose-derived stem cell therapy for ulcers in patients with critical limb ischemia
Source: PLoS One. 2025 Apr 9;20(4):e0318922. doi: 10.1371/journal.pone.0318922 (PMC11981164; doi:10.1371/journal.pone.0318922)
Supplement: S2 File — (PDF) [file pone.0318922.s002.pdf]

CONSENTIMENTO LIVRE E ESCLARECIDO (TCLE)
RESOLUÇÃO 466/2012

CONVIDO, o Senhor(a) para participar do Projeto de Pesquisa intitulado "**ESTUDO CLÍNICO RANDOMIZADO, PROSPECTIVO, CONTROLADO, UNICÊNTRICO E ABERTO DE FASE II PARA AVALIAR A SEGURANÇA E A EFICÁCIA DA TERAPIA COM CÉLULAS-TRONCO MESENQUIMAIS AUTÓLOGAS PARA ÚLCERAS EM PORTADORES DE ISQUEMIA CRÍTICA DE MEMBROS INFERIORES – ESTUDO STEM-CELL I**", que será desenvolvido por mim Prof. Dr. Matheus Bertanha, Cirurgião Vascular, com a colaboração dos Profs. Drs. Marcone Lima Sobreira, Cirurgião Vascular.

Estou estudando a doença arterial periférica (DAP), responsável pela diminuição da circulação sanguínea dos membros inferiores e causadora das suas feridas que não cicatrizam. Para que eu possa ter um resultado nesse momento preciso coletar 20ml do seu sangue que será utilizado para realização dos seguintes exames laboratoriais (hemograma completo, ureia, creatinina, sódio, potássio, glicose em jejum, hemoglobina glicada, CPK, TGO, TGP, PCR, bilirrubinas, PTF, coagulograma, Ácido Úrico, Colesterol total,HDL, LDL, Triglicérides, HIV tipo I e II, HTLV tipo I e II e Hepatites B e C). Isso será realizado em três momentos durante o estudo ou mais vezes se considerarmos necessário, sendo a primeira coleta antes de qualquer procedimento do estudo, para averiguarmos as condições gerais de sua saúde. O risco com a coleta de sangue será a picadinha da agulha e uma manchinha roxa que desaparecerá bem rapidamente.

Você será incluído no estudo para participar de forma aleatória de dois possíveis grupos de tratamento e não será possível fazer a mudança de um grupo para o outro. Um dos grupos será o grupo de pacientes que receberão tratamento apenas com curativos com Hidrogel, sendo que se você participar neste grupo, você terá uma rotina de consultas para comparecer e deverá seguir corretamente as instruções que serão dadas mais à frente. Da mesma forma, você poderá participar do grupo que será submetido ao tratamento com células-tronco mesenquimais (CTM) que serão retiradas de você mesmo(a). Dois procedimentos terão que ser realizados. No primeiro, será agendada uma pequena cirurgia para coleta de um pequeno fragmento de pele e gordura da sua barriga com 2cm de diâmetro, que será realizada com anestesia local e sem necessidade de internação hospitalar. Esse material será levado para o nosso laboratório e dele vamos retirar as CTM e alimenta-las para que aumentem em número. Se você participar deste grupo, os riscos desse primeiro procedimento são pequenos, tais como: infecção, não cicatrização da ferida, alergia ao medicamento anestésico, entre outros. O segundo procedimento será realizado três ou quatro semanas após a coleta da gordura da barriga. Mais uma vez, precisaremos de um pouco do seu sangue, em torno de 50ml, já na hora que você chegar para ser atendido(a), para fazermos os preparos das suas células. Assim que as células estiverem preparadas, em torno de 3 horas depois do atendimento, você será submetido(a) a um procedimento cirúrgico com anestesia por bloqueio local ou por raquianestesia e alguma sedação para você não sentir dor, que será escolhido pelo anestesiologista de forma que seja o mais seguro para você. Depoisdas preparações cirúrgicas, será feita uma boa limpeza da ferida, a injeção das suas CTM por pequenas punções nas bordas da ferida e um curativo contendo CTM também será colocado na ferida. Você receberá orientações de cuidados com esse curativo. Neste momento, os riscos relacionados serão: os riscos anestésicos; alergias aos medicamentos, além da possibilidade de não funcionamento da nova técnica. Você deve receber alta ainda neste mesmo dia.

Informo que o material biológico colhido do Senhor(a), CTM obtidas do tecido gorduroso da barriga,não será usado em sua totalidade, sendo que parte desse material será utilizado para controles rigorosos da sua qualidade e parte será armazenado na Faculdade de Medicina. Esse material será utilizado somente no tempo de vigência desta pesquisa (2 anos a partir da coleta), estritamente como foi explicado e após os dois anos será descartado.

Solicito também seu consentimento para consultar seu prontuário médico para coletar outras informações contidas em consultas feitas pelo (a) Senhor (a) e dados que possam ser relevantes a essa pesquisa

Além disso, o(a) Senhor(a) deverá comparecer em consultas que serão agendadas (7 dias, 30 dias, 60dias, 90 dias e 120 dias) para avaliação dos curativos. Nestas consultas você responderá alguns questionários para o acompanhamento da sua saúde, o que levará cerca de 15 minutos de duração, em cada consulta.

Informo também que, participando em qualquer dos dois grupos, alguns outros exames serão realizados, como ultrassonografia vascular com Doppler, Termografia da pele com uso de uma câmera fotográfica especial, aferição dos níveis de oxigênio e gás carbônico da pele por um eletrodo simples, o que vai demorar por volta de uma hora e será realizado em 3 consultas. A sua úlcera será fotografada apenas parauso na pesquisa ou para demonstração de resultado científico sem que o(a) senhor(a) seja identificado(a). Todos esses procedimentos não precisam de nenhum preparo e não devem expor você a nenhum risco adicional.

Seu benefício em participar da pesquisa será o de ter a chance de receber um novo tratamento com CTM retiradas de você mesmo(a) que pode vir a ajudar na melhora da sua circulação e aumentar as chances de cura da sua úlcera. Caso não houver benefício neste momento e de forma direta para o Senhor(a), esta pesquisa trará benefícios para futuros pacientes, após a coleta e conhecimento dos resultados dessa pesquisa.

Fique ciente de que sua participação neste estudo é voluntária e que mesmo após ter dado seu consentimento para participar da pesquisa, você poderá retirá-lo a qualquer momento, sem qualquer prejuízo na continuidade do seu tratamento.

Este Termo de Consentimento Livre e Esclarecido foi elaborado em 2 vias de igual teor, o qual 01via será entregue ao Senhor(a) devidamente rubricada, e a outra via será arquivada e mantida pelos pesquisadores por um período de 5 anos após o término da pesquisa.

Qualquer dúvida adicional você poderá entrar em contrato com o Comitê de Ética em Pesquisa através dos telefones (14) 3880-1608 ou 3880-1609. Ele funciona de 2ª a 6ª feira das 8.00 às 11.30 e das 14.00 às 17horas, na Chácara Butignolli s/nº em Rubião Júnior – Botucatu - São Paulo. Os dados de localização dos pesquisadores estão abaixo descritos.

Após terem sido sanadas todas minhas dúvidas a respeito deste estudo, CONCORDO EM PARTICIPAR de forma voluntária deste estudo, estando ciente que todos os meus dados estarão resguardados através do sigilo que os pesquisadores se comprometeram. Estou ciente que os resultados desseestudo poderão ser publicados em revistas científicas, sem, no entanto, que minha identidade seja revelada.

Botucatu,_______/_____/20_____.

____ _______________________________________

Pesquisador Participante da Pesquisa

Nome: Matheus Bertanha
Endereço: Av. Prof. Mário Rubens Guimarães Montenegro, s/n. Bairro: UNESP - Campus de Botucatu CEP 18618687 - Botucatu, SPPABX: (14) 3880-1001
Telefone: (14) 38801444
Email: matheusbertanha@fmb.unesp.br

TERM OF CONSENT FREE AND ENLIGHTENED (TCLE)
RESOLUTION 466/2012

I INVITE you to participate in the Research Project entitled " **CLINICAL STUDY RANDOMIZED, PROSPECTIVE, CONTROLLED, SINGLE-CENTER AND OPEN PHASE II TO TO ASSESS THE SECURITY AND THE EFFECTIVENESS FROM THE THERAPY WITH STEM CELLS MESENCHYMAL AUTOLOGOUS FOR ULCERS IN CARRIERS OF CRITICAL ISCHEMIA OF LOWER LIMBS – STEM-CELL STUDY I** ", which will be developed by me Prof. Dr. Matheus Bertanha, Vascular Surgeon, with the collaboration of Profs. Drs. Marcone Lima Sobreira, SurgeonVascular.

I am studying the disease peripheral arterial (DAP), responsible for the decrease of circulation blood in the lower limbs and causing their wounds that do not heal. So that I can have a result at this point I need to collect 20ml of your blood which will be used to perform the following exams laboratory (blood count full, urea, creatinine, sodium, potassium, glucose in fast, hemoglobin glycated, CPK, TGO, TGP, PCR, bilirubin, PTF, coagulogram, Acid Uric, Cholesterol total,HDL, LDL, Triglycerides, HIV type I and II, HTLV type I and II and Hepatitis B and C). This will be done in three moments during the study or more times if we consider it necessary, with the first collection being before any study procedure, to assess your general health conditions. The risk of collecting of blood it will be the minced meat from the needle and a little spot purple that will disappear good quickly.

You it will be included node study to to participate of form random of two possible groups of treatment and it will not be possible to change from one group to another. One of the groups will be the patients who will receive treatment only with Hydrogel dressings, and if you participate in this group, you will have a routine of appointments to attend and you must correctly follow the instructions that will be given more the front. From the same form, you may to participate of group that will be submitted to thetreatment with stem cells mesenchymal (CTM) what will be withdrawals of you same. Two procedures will have to be performed. In the first, a small surgery will be scheduled to collect a small fragment of skin and fat from your belly measuring 2cm in diameter, which will be performed under anesthesia local and without the need for hospital admission. This material will be taken to our laboratory and from there we will remove the CTM and feed them so that they increase in number. If you participate in this group, the The risks of this first procedure are small, such as: infection, non-healing of the wound, allergy to the anesthetic medication, among others. The second procedure will be performed three or four weeks after the collection of belly fat. Again, we will need some of your blood, around 50ml, as when you arrive for care, so we can prepare your cells. As soon as the cells are prepared, around 3 hours after the appointment, you will undergo a surgical procedure with local block or spinal anesthesia and some sedation for you no to feel pain, what it will be chosen for the anesthesiologist of form what it is the more safe to you. Afterof the surgical preparations, a good cleaning of the wound will be done, the injection of your MSCs by small punctures on the edges of the wound and a dressing containing MSC will also be placed on the wound. You will receive guidelines of care with that dressing. In this moment, you risks related will be: you risks anesthetics; allergies to medications, in addition to the possibility of the new technique not working. You must receive high yet in this same day.

I hereby inform you that the biological material collected from you, CTM obtained from the fatty tissue of your belly,will not be used in its entirety, as part of this material will be used for strict controls of the its quality and part will be stored at the Faculty of Medicine. This material will only be used in the duration of this research (2 years from the collection), strictly as explained and after the two years will be discarded.

I request also your consent to consult your medical record doctor to collect others information contained in queries made by you and data that may be relevant to this search

In addition, you must attend appointments that will be scheduled (7 days, 30 days, 60 days)days, 90 days and 120 days) for evaluation of dressings. During these consultations you will answer some questionnaires for the monitoring of your health, what will take about of 15 minutes of duration, in each query.

I inform also what, participating in any of the two groups, some others exams will be performed, such as vascular ultrasound with Doppler, Thermography of the skin using a camera special photographic, measurement of oxygen and carbon dioxide levels in the skin using a simple electrode, which will take about an hour and will be performed in 3 consultations. Your ulcer will be photographed only foruse in research or to demonstrate scientific results without you being identified. All these procedures do not require any preparation and should not expose you to any risk. additional.

Your benefit in participating in the research will be to have the chance to receive a new treatment with CTM taken from yourself that can help improve your circulation and increase your chances healing of your ulcer. If there is no benefit at this time and directly for you, this search will bring benefits to futures patients, after the collect and knowledge of the results of this search.

Please be aware that your participation in this study is voluntary and that even after you have given your consent, consent to participate in the research, you may withdraw it at any time, without any prejudice in continuity of your treatment.

This Term of Consent Free and Enlightened he was elaborated in 2 ways of equal content, the which 01via it will be delivered to the Madam) properly initialed, and the other via it will be archived and maintained by the researchers by one period of 5 years after the end from the search.

If you have any additional questions, you can contact the Research Ethics Committee. through of the phones (14) 3880-1608 or 3880-1609. He works of 2nd the 6th fair of the 8.00 to the 11.30 and of the 2:00 pm to 5:00 pm, at Chácara Butignolli s/nº in Rubião Júnior – Botucatu - Sao Paulo. The data from location of the researchers they are described below.

After have been healed all my doubts the respect of this study, I AGREE IN TO PARTICIPATE of form volunteer of this study, being aware what all you my data will be protected by the confidentiality to which the researchers have committed. I am aware that the results of thisstudy may to be published in magazines scientific, without, in the however, what my identity it is revealed.

Botucatu, _____/ _____/20 _____.

____ ____

Participating Researcher from the Search

Name: Matheus Bertanha
Address: Av. Prof. Mário Rubens Guimarães Montenegro, s/n.
Neighborhood: UNESP - Campus of Botucatu CEP 18618687 -
Botucatu, SPPABX: (14) 3880-1001
Telephone: (14) 38801444
E-mail: matheusbertanha@fmb.unesp.br
