# Supplementary material for: Study protocol for a randomized clinical trial evaluating the safety and efficacy of autologous adipose-derived stem cell therapy for ulcers in patients with critical limb ischemia
Source: PLoS One. 2025 Apr 9;20(4):e0318922. doi: 10.1371/journal.pone.0318922 (PMC11981164; doi:10.1371/journal.pone.0318922)
Supplement: S3 File — (DOCX) [file pone.0318922.s003.docx]

| **Table 1**. Assessment Schedule | | | | | | | |
| --- | --- | --- | --- | --- | --- | --- | --- |
| **PROCEDURES** | **Screening** | **Assessment** | | | | | |
|  |  | **1** | **2** | **3** | **4** | **5** | **6 – Final** |
|  | -1 to -30 days | Day 0 | 7 days after inclusion  (+/-3 days) | 30 days after inclusion  (+/-3 days) | 60 days after inclusion  (+/-3 days) | 90 days after inclusion  (+/-3 days) | 120 days after inclusion  (+/-3 days) |
| Free and informed consent form (ICF) | X |  |  |  |  |  |  |
| Medical assessment: history, physical examination | X | X | X | X | X | X | X |
| Vital signs and anthropometry | X | X | X | X | X | X | X |
| Concomitant medications | X | X | X | X | X | X |  |
| Inclusion and exclusion criteria | X | X |  |  |  |  |  |
| Pain scale (VAS)[39] | X | X | X | X | X | X |  |
| SF-36[38] and EQ-5D[42] | X | X | X | X | X | X |  |
| Ulcer assessment, photography, and dressing | X | X | X | X | X | X |  |
| Ankle arm index (ABI) | X |  |  | X | X | X |  |
| WIfI[45,46] | X | X | X | X | X | X |  |
| Thermography | X |  | X | X | X | X |  |
| Vascular Doppler ultrasound | X |  |  | X |  | X |  |
| Transcutaneous monitoring | X |  | X | X | X | X |  |
| Blood sampling | X |  |  | X |  | X |  |
| HIV, HTLV, Hepatitis B, and C screening | X |  |  |  |  |  |  |
| Complete blood count | X |  |  | X |  | X |  |
| Blood glucose | X |  |  | X |  | X |  |
| Glycated hemoglobina | X |  |  | X |  | X |  |
| Total cholesterol, LDL, HDL, triglycerides | X |  |  | X |  | X |  |
| Total bilirubin and fractions | X |  |  | X |  | X |  |
| ALP | X |  |  | X |  | X |  |
| ALT | X |  |  | X |  | X |  |
| AST | X |  |  | X |  | X |  |
| GGT | X |  |  | X |  | X |  |
| Total Proteins, albumin, and globulin | X |  |  | X |  | X |  |
| PT and PTT | X |  |  | X |  | X |  |
| Immune-modulating cytokines: Th1, Th2 e Th17 | X |  |  | X |  | X |  |
| Adipose tissue sample collection | X |  |  |  |  |  |  |
| Randomization | X |  |  |  |  |  |  |
| ASC application (G1) and dressing (G2) |  | X |  |  |  |  |  |
| Adverse events screening |  | X | X | X | X | X | X |
| Discontinuation criteria |  | X | X | X | X | X |  |
| Medical discharge |  |  |  |  |  |  | X |
| Participants in the control group can be subjected to screening and assessment 1 on the same day as long as the results of the screening tests have already been checked by an investigator. | | | | | | | |
