# Supplementary material for: Study protocol for a randomized clinical trial evaluating the safety and efficacy of autologous adipose-derived stem cell therapy for ulcers in patients with critical limb ischemia
Source: PLoS One. 2025 Apr 9;20(4):e0318922. doi: 10.1371/journal.pone.0318922 (PMC11981164; doi:10.1371/journal.pone.0318922)
Supplement: S4 File — (PDF) [file pone.0318922.s004.pdf]

ESTUDO CLÍNICO RANDOMIZADO, PROSPECTIVO, CONTROLADO, UNICÊNTRICO E FASE I/II PARA AVALIAR A SEGURANÇA E A EFICÁCIA DA TERAPIA COM CÉLULAS-TRONCO MESENQUIMAIS AUTÓLOGAS EM ÚLCERAS DE PORTADORES DE ISQUEMIA CRÍTICA DE MEMBROS INFERIORES – ESTUDO STEM CELL I

ESTUDO STEM CELL I

Prof Dr. Matheus Bertanha

Botucatu2023

IDENTIFICAÇÃO DA PROPOSTA, PROPONENTE E INSTITUIÇÃO EXECUTORA	
**TÍTULO DO PROJETO**	ESTUDO CLÍNICO RANDOMIZADO, PROSPECTIVO, CONTROLADO, UNICÊNTRICO E FASE I/II PARA AVALIAR A SEGURANÇA E A EFICÁCIA DA TERAPIA COM CÉLULAS-TRONCO MESENQUIMAIS AUTÓLOGAS EM ÚLCERAS DE PORTADORES DE ISQUEMIA CRÍTICA DE MEMBROS INFERIORES – ESTUDO STEM CELL I
**NOME DO PROPONENTE**<br>**Currículo Lattes CNPqRegime de Trabalho**<br>**Cargo**<br><br>**Contatos**	MATHEUS BERTANHA<br>http://lattes.cnpq.br/4513014379461383Regime RDIDP<br>Prof. Assist. Dr. da Disciplina de Cirurgia Vascular do Depto. Cirurgia e Ortopedia<br>matheusbertanha@gmail.com matheus.bertanha@unesp.br<br>Telefone: 14 3880-1444<br>Celular: 14 997351711
**CPF**	259.258.048-48
**INSTITUIÇÃO EXECUTORA**	FACULDADE DE MEDICINA DA UNIVERSIDADE ESTADUAL PAULISTA – CAMPUS DE BOTUCATU UNESP<br>CNPJ – 48.031.918/0019-53<br>Avenida Mário Rubens Guimarães Montenegro s/nRubião Júnior<br>CEP - 18618-687
**INSTITUIÇÕES/EMPRESAS PARCEIRAS**	
**ÁREA**	ÁREA: CIÊNCIAS DA SAÚDE<br>Subárea: Medicina – Clínica Médica – AngiologiaÁrea do Conhecimento CNPq – 4.01.01.01-0

PRAZOS DE EXECUÇÃO DO PROJETO
Duração total: 36 meses Data de Início: 01/12/2023
Data de encerramento do estudo: 01/12/2026

Solicita-se sigilo de patente ao analisar o conteúdo deste projeto.

ESTUDO CLÍNICO RANDOMIZADO, PROSPECTIVO, CONTROLADO, UNICÊNTRICO E ABERTO DE FASE II PARA AVALIAR A SEGURANÇA E A EFICÁCIA DA TERAPIA COM CÉLULAS-TRONCO MESENQUIMAIS AUTÓLOGAS PARA ÚLCERAS EM PORTADORES DE ISQUEMIA CRÍTICA DE MEMBROS INFERIORES – ESTUDO STEM CELL I

Resumo

Contexto: A doença arterial periférica (DAP) pode evoluir para a isquemia crítica do membro (ICM) inferior acometido, caracterizada por dor em repouso, ulcerações ou gangrenas, com elevado risco de amputação. Nessa fase, o melhor tratamento é a revascularização arterial do membro, mas nem sempre issoé possível ou mesmo eficaz para promover o alívio da dor, cicatrização de úlceras ou evitar as amputações, além do alto custo socioeconômico provocado pela doença. Avanços recentes em terapia celular representamuma alternativa coadjuvante promissora para o tratamento da DAP em casos onde as alternativas convencionais tenham se esgotado. **Objetivo**: Avaliar a segurança e a eficácia da terapia celular com células-tronco mesenquimais autólogas expandidas no tratamento de portadores de DAP com ICM e úlceras arteriais crônicas. **Métodos**: Um estudo clínico randomizado aberto será feito com 2 grupos de 20 pacientes com ICM: no grupo 1 será coletado um fragmento de tecido adiposo abdominal (10g) para obtenção de células-tronco mesenquimais, que serão expandidas e aplicadas utilizando injeção subcutânea perilesionalno membro inferior acometido, além da aplicação na forma de biocurativo nas feridas. O grupo 2 receberá tratamento convencional com curativo com Hidrogel com ácidos graxos essenciais. Serão realizadas avaliações clínicas periódicas, exames complementares e registro fotográfico. O principal desfecho de eficácia será a cicatrização parcial ou total da ferida. Desfechos de segurança serão monitorados em relaçãoa infecções, gangrenas, amputações e óbitos. O acompanhamento dos participantes será por 120 dias. Casos de amputação não serão incluídos. Os resultados serão avaliados por avaliador externo independente e cego para os grupos. Trata-se de procedimento inovador de alto impacto e retorno financeiro para o SUS, tendo em vista a alta prevalência da doença e o alto impacto socioeconômico da doença quando evolui para amputação de membros.

Palavras chave: Terapia Baseada em Transplante de Células e Tecidos, Cicatrização de Feridas, Doença Arterial Periférica; Células-Tronco Mesenquimais; Ensaio Clínico Fase II; Amputação.

Terapia Celular, Cicatrização de Feridas, Doença Arterial Periférica; Células-Tronco Mesenquimais;Amputação.

Randomized, prospective, controlled, unicentric and open phase ii clinical trial to analise the safetyand efficacy of autologous stem cell treatment in peripheral arterial ischemic of lower limbs

Abstract

Background: Peripheral arterial disease (PAD) can progress to critical limb ischemia (CLI) of the affected lower limb, characterized by pain at rest, ulcerations or gangrene, with a high risk of amputation. In this phase, the best treatment is arterial limb revascularization, but this is not always possible or even effective for promoting pain relief, healing of ulcers or preventing amputations, in addition to the high socioeconomiccost caused by the disease. Recent advances in cell therapy represent a promising supporting alternative for the treatment of PAD in cases where conventional alternatives have run out. **Objective**: To evaluate the safety and efficacy of cell therapy with expanded autologous mesenchymal stem cells in the treatment of patients with PAD with CLI and chronic arterial ulcers. **Methods**: An open randomized clinical study will be performed with 2 groups of 20 patients with CLI: in group 1, a fragment of abdominal fat tissue (10g)will be collected to obtain mesenchymal stem cells, which will be expanded and applied using subcutaneousperilesional injection in the affected lower limb, in addition to the application in the form of a personalized curative biological on the wounds. Group 2 will receive conventional treatment with a Hydrogel dressing with essential fatty acids. Periodic clinical evaluations, complementary exams and photographic record will be carried out. The main outcome of effectiveness will be partial or total wound healing. Safety outcomes will be monitored for infections, gangrene, amputations and deaths. Participants will be monitored for 120 days. Major amputation cases will not be included. An independent external evaluator and blind to the groups will evaluate the results. It is an innovative procedure with high impact and financial return for SUS, in view of the high prevalence of the disease and the high socioeconomic impact of the disease when it progresses to limb amputation.

Keywords: Cell and Tissue-Based Therapy, Wound Healing, Peripheral Arterial Disease, Stem Cells, Clinical Trial Phase II, Amputation.

ÍNDICE

Resumo..4
Abstract..5
LISTA DE TABELAS.. 9
LISTA DE FIGURAS.. 9
LISTA DE QUADROS.. 10
1. FUNDAMENTAÇÃO E CONTEXTUALIZAÇÃO..13
1.1 Úlceras 15
1.2 Úlceras em Doença Arterial Periférica e Isquemia crítica..15
1.3 Mecanismos de cicatrização e cronificação das feridas..16
1.4 Curativos ou coberturas..18
1.5 Células-tronco mesenquimais..18
1.6 Estado atual da terapia celular para Doença Arterial Periférica e Isquemia Crítica..19
2. JUSTIFICATIVA..20
3. OBJETIVOS..21
3.1 Objetivos Primário..21
3.2 Objetivos Secundários..22
4. DESENHO DO ESTUDO..22
4.1. Descrição do desenho do estudo..22
4.2. Justificativa para o desenho do estudo..22
4.3 Local do Estudo e ética..23
4.4 Cálculo amostral e tratamento estatístico dos resultados..23
4.4.1 Tamanho da Amostra e Recrutamento..23
4.4.2 Grupos proposto no ensaio clínico..23
4.5 Critérios de Elegibilidade..24
4.5.1 Critérios de Inclusão..24
4.5.2 Critérios de Exclusão..25
4.6 Randomização..26
4.6.1 Metodologia e garantia de qualidade de resultados no estudo aberto..27
4.7 Procedimentos do estudo clínico nos grupos propostos..27
4.7.1 Coleta de Tecido Adiposo para obtenção de células-tronco mesenquimais autólogas para uso no Grupo 1 27
4.7.2 Processamento da amostra de Tecido Adiposo..28
4.7.3 Procedimentos de Expansão Celular..28
4.7.4 Controle de qualidade celular..29
4.7.5 Controle microbiológico da cultura celular..29
4.7.6 Controle de qualidade das célula-tronco..29
4.7.7 Avaliação de expressão gênica por RT-qPCR..30
4.8 Justificativa para quantidade de células (Dose)..31
4.9 Preparação, armazenamento e procedimentos para dispensação do produto..31
4.10 Procedimento de aplicação do produto..32
4.11 Controle de qualidade do estudo..33
4.11.1 Procedimentos de amostragem..33
4.11.2 Requisitos para aceitação de lote de materiais, reagentes e produtos para diagnóstico *in vitro* utilizados na manufatura do produto e nos processos de controle de qualidade..35
4.11.2 Requisitos de segurança e qualidade para liberação do produto de terapia avançada investigacional para administração..35
4.11.3 Caracterização do componente ativo, incluindo, quando couber, sua identidade, quantidade, pureza, viabilidade, potência, estabilidade genética e esterilidade, devendo descrever as metodologias analíticas empregadas..38
4.12 Reações ou riscos relativos ao tratamento..38
4.13 Seguimento, Avaliações, Procedimentos e Cronograma de Visitas..38
4.13.1 Avaliações Clínicas..38
4.13.2 Avaliação da úlcera:..40
4.13.3 Avaliações Laboratoriais..40
4.13.4 Curativos..41
4.13.5 Avaliação de Acompanhamento..41

4.13.6 Critérios de descontinuação no estudo 43
4.14 Desfechos 43
4.14.1 Desfecho primário 43
4.14.2 Desfecho secundário 43
4.15 Coleta e monitoramento dos dados 44
4.15.1 Coleta e monitoramento dos dados do estudo clínico 44
4.15.2 Plano de Monitoria do estudo clínico 47
4.15.3 Relato e Gerenciamento de Eventos Adversos 48
4.16 Comitê Independente de Monitoramento de Segurança 49
4.17 Processo de relatório de segurança 49
4.18 Plano de análise interinas 49
4.19 Medicações concomitantes 50
4.20 Critérios de descontinuidade dos participantes 50
4.21 Métodos estatísticos 50
5. RESPONSABILIDADES 51
5.1 Boas Práticas Clínicas 51
5.2 Princípios Éticos 51
5.2.1 Leis e Regulamentações 51
5.2.2 Consentimento Livre e Esclarecido 51
5.2.3 Comitê de Ética em Pesquisa (CEP)/CONEP 51
5.2.4 Emenda ao protocolo de estudo clínico 52
5.2.5 Desvios ao Protocolo 52
5.3 Definição dos Dados-fonte 52
5.3.1 Retenção de registro no centro de estudo – Arquivo de documentos 52
5.4 Compensação do Seguro 53
5.5 Publicações e Comunicações 53
5.6 Controle de Qualidade e Garantia de Qualidade 53
6. RESULTADOS ESPERADOS E SUA RELEVÂNCIA PARA OS DOENTES E O SUS 54
6.1 Resultados esperados 54
6.2 Alinhamento com políticas públicas de saúde 55
6.3 Impactos para o sus 56
7. Custo-Efetividade 56
8. Impactos e contribuições para o complexo econômico-industrial da saúde 57
9. CARÁTER INOVADOR DO PROJETO 58
9.1 Estudo de viabilidade patentária 58
10. PLANEJAMENTO DA GESTÃO DE RISCOS: 59
11. PROPRIEDADE INTELECTUAL 59
12. INFRAESTRUTURA INSTITUCIONAL E CAPACIDADE TÉCNICA OPERACIONAL DA EQUIPE DO ESTUDO 60
12.1. Infraestrutura institucional 60
12.1.1 Unidade de Pesquisa Clínica da FMB/UNESP - UPECLIN 60
12.1.2 Laboratórios de Pesquisa do Hemocentro de Botucatu 61
12.2 Capacidade intelectual e técnica operacional da equipe do estudo 62
12.2.1 Compilação sucinta das atividades de pesquisa desenvolvidas, consideradas pelo requerente as mais relevantes, indicando a produção gerada por elas até 2019 62
12.2.2 Compilação sucinta das atividades de pesquisa desenvolvidas, consideradas pela colaboradora principal as mais relevantes, indicando a produção gerada por elas até 2019 64
13. PLANO DE TRABALHO 66
13.1 Identificação do objeto a ser executado - Plano de trabalho 66
13.2 Elaboração, preparação e envio de dossiês regulatórios 68
13.3 Início da execução do estudo clínico 68
13.4. Execução do estudo clínico 68
13.5 Fim do período de inclusão de participantes no estudo 68
13.6 Encerramento da execução do estudo clínico 68
13.7 Gerenciamento dos dados e estatística 69
13.8 Elaboração e envio de relatórios 69

13.9 Participação em eventos científicos divulgação dos resultados 69
14. ORÇAMENTO DETALHADO 69

15. RESUMO DOS INVESTIMENTOS 74
16. CONTRAPARTIDA DA INSTITUIÇÃO 74
17. REFERÊNCIAS BIBLIOGRÁFICAS...74
18. Anexos e Apêndice 79

LISTA DE TABELAS

Tabela 1. Classificação de Rutherford e Fontaine para DAP........ 14
Tabela 2. Critérios de inclusão 24
Tabela 3. Critérios de exclusão........ 25
Tabela 4. Padrão de conformidade para células-tronco mesenquimais 30
Tabela 5. Cronograma de Avaliações e Visitas........ 41
Tabela 6. Cronograma 66

LISTA DE FIGURAS

Figura 1. Fluxo Monitoramento dos dados da pesquisa clínica com terapia celular avançada proposta nesteestudo.47

LISTA DE QUADROS

Quadro 1: Gestão de riscos relativos a execução do presente estudo........ 57

1. FUNDAMENTAÇÃO E CONTEXTUALIZAÇÃO

A aterosclerose é uma doença cardiovascular crônica e progressiva que se manifesta mais frequentemente, a partir da idade adulta, sendo caracterizada pela inflamação crônica da túnica íntima das artérias de grande e médio calibre (aterosclerose), ocorrendo devido ao acúmulo e oxidação de lipoproteínas na parede arterial, provocando uma série de lesões multifocais, sendo a mais comum, a placa de ateroma, que cresce para dentro da luz, podendo restringir fluxo para os segmentos distais por estreitamento parcial (estenoses) ou oclusão total da luz.[1] É considerada a principal causa de mortalidade dos países industrializados.[1-4] Com a redução do fluxo sanguíneo para o membro acometido, ocorre diminuição do aporte de nutrientes e oxigênio aos tecidos e, consequentemente, isquemia. [5, 6]

A doença arterial periférica (DAP) é a manifestação da doença aterosclerótica que afeta a circulação arterial periférica, principalmente dos membros inferiores, com frequência estimada em estudos internacionaisde 10% a 12% na população adulta e 20% da população acima dos 75 anos, com predominância para o sexo masculino.[7] De modo geral, a incidência estimada de isquemia crítica de membros (ICM) é de 500 a 1.000 membros inferiores acometidos por milhão de habitantes por ano.[7] Entre esses doentes, o índice de amputação primária varia de 10% a 40%, caracterizando um grande problema de saúde pública. Quanto aos fatores de risco, são considerados maiores: diabetes *mellitus* tipo 2 (DMT2), o tabagismo, a hipertensão arterial sistêmica (HAS) e a hiperlipidemia, responsáveis por 80 a 90% das doenças cardiovasculares.[7] Outrosfatores de risco tem sido implicados como atuantes na gênese do processo aterosclerótico, mas devido aoseu papel eventualmente secundário ou a necessidade de mais estudos controlados que indiquem seu real papel na aterogênese são considerados fatores de risco menores; entre eles: hiperhomocisteinemia, hiperfibrinogenia, fatores inflamatórios, fatores genéticos, agentes infecciosos, alterações no metabolismo do cálcio, estados de hipercoagulabilidade, desequilíbrio de metaloproteinases, insuficiência renal e estresse oxidativo.[8, 9]

Os sinais e sintomas da DAP resultam da limitação do fluxo sanguíneo nas artérias dos membros inferiores, imposta por uma estenose crítica ou oclusão arterial, secundárias à formação da placa aterosclerótica, manifestando clinicamente a claudicação intermitente, sintoma inicial patognomônico da DAP, caracterizada pelo doente como a sensação de fadiga muscular, adormecimento do membro, aperto na panturrilha, câimbra ou paralisia em certos grupamentos musculares do membro inferior, desencadeadas pelo exercício físico e melhorando com a sua interrupção, normalmente limitando as caminhadas a distâncias específicas.[10, 11] Os sintomas podem, ainda, se manifestar na coxa ou nas nádegas, e associar-se à impotência sexual, dependendo da artéria e da extensão da lesão.[12, 13] Na avaliação clínica, manifestam diminuição ou ausência de pulsos e sinais exteriores de isquemia, como cianose, palidez, fragilidade da pele, unhas quebradiças, ausência de pelos e úlceras, caracterizados como sinais tróficos da doença isquêmica periférica.[14] Com a evolução da doença o doente vai limitando sua caminhada a distâncias cada vez menores,

com intervalos de tempo para recuperação cada vez maior.

Ao final do ciclo de estabilidade da doença, a isquemia tecidual grave começa a comprometer a funcionalidade dos tecidos, causando limitação extrema da movimentação, dor em repouso, úlceras e gangrenas, o que caracteriza a ICM.[15] O doente passa a adotar posições antálgicas (flexão joelhos oumembro pendente, massageando o local ou forçando a deambulação), evoluindo com piora progressiva do fluxo sanguíneo.[16] A doença arterial periférica pode

ser classificada através de parâmetros clínicos propostos por Rutherford[17] e também por Fontaine,[18] conforme apresentado no Tabela 1.

Tabela 1. Classificação de Rutherford e Fontaine para DAP

**Fontaine**		**Rutherford**		
**Estágio**	**Quadro Clínico**	**Categoria**	**Quadro Clínico**	**Dados Laboratoriais**
I	Assintomático	0	Assintomáticos	Lesões sem repercussão hemodinâmica. Teste esteira normal.
IIa	Claudicação leve	1	Claudicação leve	Completa o exercíciuo de esteira; índice tornozelo braquial >50mmHg mas no mínimo 20mmHg menor que o estado em repouso.
IIb	Claudicação moderada a grave	2	Claudicação moderada	Entre a categoria 1 e 3
		3	Claudicação grave	Não termina teste esteira, índice tornozelo braquial <50mmHg
III	Dor em repouso	4	Dor isquêmica emrepouso	Índice tornozelo braquial em repouso <40mmHg; pressão de tonozelo ou volume de pulso metatarsal ou indice de hálux <30mmHg
		5	Perda tecidual pequena	Índice tornozelo braquial em repouso <60mmHg; pressão de tonozelo ou volume de pulso metatarsal ou indice de hálux <40mmHg
IV	Úlcera ou gangrena	6	Perda tecidual queavança o antepé	Idênticos ao da categoria 5

Desse ponto em diante, quando observamos as classificações clínicas de Fontaine IIb a IV e Rutherford categoria 3 a 6, o tratamento clínico não promove mais incremento de qualidade de vida para o paciente, necessitando de uma intervenção com revascularização cirúrgica, sendo este considerado o tratamento padrão para DAP.[7] As opções cirúrgicas de revascularização atualmente disponíveis são: pontes arteriais ou by-pass (com uso de veia autóloga ou próteses sintéticas), tratamentos endovasculares ou angioplastias arteriais transluminais percutâneas (com balões, stents, endopróteses e aterótomos), além de tratamentos paliativos como simpatectomias e neurotripsias, sendo que ao final restam apenas as amputações menores ou maiores.[7, 19] Muitos pacientes, mesmo nas melhores condições de tratamento cirúrgico persistem com úlceras de difícil cicatrização (Fontaine IV, Rutherford categoria 5 e 6),

principalmente as decorrentes de amputações menores, que frequentemente necessitam de cicatrização por segunda intenção e acabam por cronificar, com alta probabilidade de evoluir abruptamente para amputação.[20-22]

1.1 Úlceras

O termo úlcera se refere à destruição da camada de epiderme, com a extensão pelos planos da pele podendo ser variável, desde limitada a derme ou eventualmente atingindo tecidos subcutâneos ou mesmo tecidos mais profundos.[23] Define-se como crônica uma úlcera que não cicatriza após 6 semanas de tratamento adequado (terminologia mais aplicável para úlceras de etiologia venosa).[24] As úlceras das extremidades inferiores são muito comuns, com uma prevalência estimada de 1 a 2% entre os adultos nos EUA[25] e sua ocorrência têm um efeito importante na saúde pública, que consome recursos, causando frustração para os profissionais de saúde e para os doentes, condicionando degradação da sua qualidade de vida e dias de trabalho perdidos.[23]

A etiologia da úlcera pode ser decorrente de diversas causas: doença arterial periférica, insuficiênciavenosa crônica, doenças neuropáticas (incluindo diabéticas), sendo essas três as responsáveis por mais de 90% dos casos, nos EUA [25] além das de origem traumática (incluindo as ocasionadas por queimaduras), osteomielite crônica, anemia falciforme, vasculites, tumores cutâneos (basocelulares e espinocelulares), doenças infecciosas crônicas (lepra, tuberculose, leishmaniose), por linfedema, por pressão e radioterapia.[26] Em aproximadamente 3,5% dos doentes, a causa da úlcera não é identificada.[27] Ressalta-se ainda, a importância do diagnóstico correto, tendo em vista que os tratamentos divergem e que a introdução da terapêutica equivocada acarretará danos ao paciente.[28]

O tratamento das úlceras geralmente envolve aspectos sistêmicos e locais, focando a limpeza e a cobertura do ferimento, até que o organismo consiga restabelecer a integridade da pele e controlar as condições de base para proporcionar melhor qualidade de vida.[29] O avanço do conhecimento nessa área propiciou o desenvolvimento de uma ampla variedade de produtos que podem ser utilizados na terapêutica das úlceras. Vale ressaltar que o tratamento desses doentes deve ser multiprofissional, dirigido não apenas à úlcera, mas sim indivíduo como um todo, pois o sucesso é diretamente ligado a mudanças em hábitos devida que o expõe a riscos.[29]

1.2 Úlceras em Doença Arterial Periférica e Isquemia crítica

As úlceras arteriais decorrem da inadequada perfusão tecidual, devido a bloqueio completo ou parcial do suprimento arterial. São encontradas principalmente em áreas de protuberância óssea como maléolos e falanges pois nestes tecidos mais distais dos membros inferiores as artérias podem ser únicas e, portanto, a angiopatia pode levar a insuficiência arterial permanente, não apresentando suficiente circulação colateral para o seu suprimento.[27, 30]

As úlceras arteriais apresentam dimensões e profundidade variável (geralmente profundas acometendo músculos e tendões), circundadas por pele de coloração pálida, com pouca quantidade de exsudato, por vezes secreção seropurulenta, discreto edema local, necrose tecidual, pele fria e atrófica, odor

fétido, difícil cicatrização, extremamente dolorosas, sendo a aterosclerose, a doença subjacente na maioria dos casos.[25]

Para validação do diagnóstico percebe-se: histórico de claudicação intermitente, lesões tróficas da pele e alteração da perfusão tissular periférica, corroborado pela ausência de pulsos, índice tornozelo braquial (ITB) menor que 0,9, além de outros exames complementares de imagem como arteriografia, ultrassonografia vascular com Doppler, angiotomografia entre outros, demonstrando alterações da circulação arterial.[7]

Deve-se levar em consideração que, a úlcera em um paciente portador de DAP indica um comprometimento tecidual muito maior do que a ferida visível, pois de um modo geral ocorre em fases maistardias da doença em consequência do grave quadro isquêmico do membro.[14] Acrescenta-se ainda o fato de que haja uma chance reduzida de resolução desses quadros sem alguma intervenção cirúrgica que restabeleça o aporte sanguíneo para esses tecidos, o que nem sempre é possível, e com isso, ocorrem elevadas taxas de amputação de membros. [14] A revascularização arterial do membro acometido é condição mandatória nesse estágio da doença, sendo que nos casos nos quais isso não é possível, seja por impossibilidade clínica do paciente, seja por inexistência de uma árvore de desague arterial ou por falha terapêutica, estes podem evoluir com amputação do membro acometido. [14] Nesta fase onde nada se pode fazer em prol da revascularização e o doente não aceita a amputação, a sua qualidade de vida assemelha-se com a de portadores de câncer terminal.[31] A amputação acarreta um declínio produtivo para os pacientes, piora da qualidade de vida e altos custos para os sistemas de saúde.[7] Acrescenta-se a isso o fato que a amputação maior das extremidades inferiores, nesses pacientes, incide um risco de mortalidade cirúrgicaque pode chegar até a 50% em algumas séries, corroborando a necessidade de se tentar a cicatrização dessas úlceras por métodos não ortodoxos.[7]

1.3 Mecanismos de cicatrização e cronificação das feridas

Após eclodir uma ferida superficial, uma sequência de eventos se sucede para eliminar os debris de tecido desvitalizado. Então, para o processo de reparação tecidual subsequente, será necessário que haja a ativação de células inflamatórias variadas, as quais são induzidas por citocinas e exposição a moléculas da matriz extracelular.[32] Uma demanda extra de nutrientes será necessária no local da ferida com a finalidadede suprir o metabolismo acelerado provocado pelo tecido em reparação. Esses processos ocorrem simultaneamente e são geralmente divididos em três fases principais para a cicatrização de feridas: inflamatória, proliferativa e remodeladora.[32]

A fase inflamatória da cicatrização da ferida começa logo após a hemostasia ser alcançada, e o objetivo primário dessa fase é limpar os patógenos, bem como o material estranho da ferida e restringir os danos a uma área localizada.[33] A permeabilidade vascular aumenta através da indução da vasodilatação, permitindo que os neutrófilos e monócitos se desloquem para o local da ferida. Uma sequência complexa de interação entre citocinas vai regular esta fase, culminando na conversão de monócitos em macrófagos, frequentemente considerados como o principal regulador desta fase inflamatória da cicatrização de feridas.[33]Os macrófagos não só fagocitam e digerem restos de tecidos e neutrófilos remanescentes, mas também secretam fatores de crescimento e citocinas que promovem a proliferação tecidual e migração celular.[34]

Após cerca de 3 dias da ferida inicial, a fase proliferativa está baseada na produção de fibroblastos e de colágeno, substância fundamental que formará o suporte básico de tecido cicatricial.[34] Enquanto isso, as células endoteliais entram em uma fase de rápido crescimento e a angiogênese ocorre dentro do tecido de granulação, criando uma rica rede vascular que fornece essa área de cura muito ativa.[34] Após cerca de 2 a 3 semanas, a ferida passa para uma fase de remodelação ou maturação no qual o tipo de colágeno é o usual (tipo I, ao invés do tipo III visto em uma nova ferida) e o tecido da ferida amadurece, resultando em pleno *cross-linking* e restauração de uma estrutura próxima do normal, o que pode ser um processo longo.[35]

Uma consideração importante na cicatrização fisiológica de feridas é o suprimento de oxigênio e a tensão de oxigênio no leito da ferida.[36] A cicatrização de feridas requer oxigênio para interagir com numerosas citocinas, suprir as células ativamente proliferativas, bem como para os neutrófilos.[36] Estima-se que uma ferida requeira pelo menos 20 mmHg de tensão de oxigênio para poder cicatrizar, sendo que tensões menores que 5 mmHg tem baixa probabilidade de cicatrização.[37] Esses efeitos parecem estar intimamente relacionados um ao outro em situações de baixa tensão de oxigênio, não somente haverá mais detritos necróticos que pode facilitar o crescimento bacteriano, como também compromete a efetividade do sistema imunológico no combate a patógenos.[37] Assim, portadores de DAP bem como portadores de DMT2,com baixas tensões de oxigênio tecidual, tem um fator negativo para a evolução de suas úlceras.

No entanto, quando outros fatores patológicos entram em jogo, como um estado de doença subjacente, uma úlcera crônica pode se formar. Isso se refere a uma ferida que de alguma forma se desviou do curso fisiológico natural de eventos anteriormente descritos e estacionou em algum momento. O mecanismo subjacente varia muito, mas inclui fatores que influenciam o suprimento de sangue (doença vascular periférica), função imunológica (como imunossupressão ou imunodeficiência adquirida), doenças metabólicas (como DMT2), medicamentos ou lesão tecidual local anterior (como radioterapia). Fatores externos como pressão sustentada, temperatura e umidade, também desempenham um papel importante em permitir ou não que uma ferida cicatrize.[38]

Nas afecções que não cicatrizam normalmente, a coordenação do processo fisiológico de reparo tecidual exercida por sinalizadores celulares (TGF-β, PDGF, IGF-1, VEGF, FGF) não ocorre de forma adequada e os mecanismos bioquímicos mediados por citocinas não são efetivos (TNF-α, IL-1, IL6, INF- γ).[39] Há uma extensa relação entre fatores vasculares, imunológicos e infecciosos que predispõem ao aparecimento de uma úlcera crônica.[35, 40]

Muitas vezes, a cronicidade das lesões ocorre como uma resposta imunológica exacerbada e prejudicial frente ao tecido danificado, não eliminando, suprimindo ou controlando o problema. Duranteuma resposta inflamatória que cronifica uma úlcera, o perfil predominante de citocinas é característico de uma resposta tipo Th2 (aumento de IL4, IL5, IL9, IL10, IL13), isto é, uma resposta inflamatória desequilibrada que combina as respostas imunes inata e celular, com a interação de diversos fatores de crescimento.[38] Além disso, o desarranjo no controle de metaloproteases (MMP) e células regenerativas. Ainda são desconhecidos todos os processos fisiopatológicos envolvidos em cada situação clínica que justifica maiores investigações.[38]

1.4 Curativos ou coberturas

O curativo ou cobertura pode ser definido como sendo um meio terapêutico que consiste na limpeza e aplicação de material sobre uma ferida para sua proteção, absorção e drenagem, com o intuito de melhorar as condições do leito da úlcera e auxiliar em sua resolução.[41] Podem ser, em algumas ocasiões, o próprio tratamento definitivo; em outras, apenas uma etapa intermediária para o tratamento cirúrgico.[41] Há, no mercado mundial, diversos materiais para uso em curativos que podem ser utilizados nas diferentes etapas de tratamento das úlceras, a saber: higienização, desbridamento, diminuição da população bacteriana, controle do exsudato, estímulo à granulação e proteção para a epitelização. Fan e cols,[42] sugerem que os curativos sejam classificados em: curativos passivos; curativos com princípios ativos; curativos inteligentes; curativos biológicos; e compostos. Outra característica dos curativos a serem utilizados para o tratamento deúlceras crônicas é que estes devem manter uma superfície úmida para prevenir a desidratação e a morte celular do tecido de reparação. Além disso, uma superfície úmida pode favorecer a angiogênese, estimular a formação do tecido de granulação e a epitelização, facilitar a remoção do tecido necrótico e da fibrina; servircomo barreira protetora contra microrganismos, promover a diminuição da dor, evitar a perda excessiva de líquidos e evitar traumas do tecido de cicatrização.[43]

Os principais tipos de curativos incluem: curativos simples clássicos (impregnados ou não com outros componentes); curativos pouco aderentes ou não aderentes (como filme de silicone, tela de poliamidacom silicone, filme transparente de poliuretano); filmes semipermeáveis; hidrogéis; hidrocolóides; alginatos;colágenos; espumas de poliuretano; polissacarídeos; açúcar; hidrofibras (carboximetilcelulose 100%); ácido hialurônico; enzimas; inibidores de proteases; carvão ativado; prata; telas de tecidos (impregnados ou não), curativos biológicos, entre outros.[44,45] Além do tratamento das úlceras com curativos, ainda são necessários desbridamentos químicos ou mecânicos, cuidados locais com a úlcera, profilaxia de infecções, antibioticoterapia, terapia antifúngica, terapias de compressão, diminuição dos pontos de atrito ou de pressão, entre outros.[46,47]

Embora haja uma grande variedade de curativos, um só tipo de curativo não preenche os requisitos para ser aplicado em todos os tipos de úlceras, sendo que, a depender da evolução da cicatrização, estes devem ser constantemente readequados.

1.5 Células-tronco mesenquimais

As células-tronco mesenquimais (CTM) estão presentes em maior ou menor quantidade em todos ostecidos do organismo, mesmo após a fase embrionária, sendo mais facilmente obtidas em alguns tecidos queas preservam em maior quantidade e com menor grau de diferenciação, como por exemplo as encontradas em sangue de cordão umbilical,[48] medula óssea[49] e tecido adiposo[50] ou até mesmo de sangue periférico após indução de proliferação medular com G-CSF.[51]

A Sociedade Internacional de Terapia Celular estabeleceu critérios de definição para as CTM, sendoque estas devem obrigatoriamente ser aderentes ao plástico do frasco de cultura durante várias passagens, deve preservar a plasticidade, ser capaz de se diferenciar em pelo menos três tecidos diferentes (cartilagem, tecido ósseo e tecido adiposo), deve expressar alguns marcadores de superfície celular (CD73, CD90 e

CD105, CD71, CD44, CD106) e não deve expressar outros (c-kit, CD11b, CD14, CD19, CD34, CD45, CD79α, CD31), além de serem negativas para os antígenos leucocitários humanos (HLA-DR).[52, 53]

O tecido adiposo representa uma abundante fonte de CTM, é de fácil obtenção cirúrgica e praticamente não apresenta contraindicação para sua obtenção. A fração do estroma vascular do tecido adiposo se tornou foco de investigações após a comprovação de suas características multipotentes, com vantagens potenciais de aplicabilidade na engenharia de tecidos.[54, 55]

Contudo, a preparação das células em laboratório para a cultura não é idêntica. A maioria dos pesquisadores isolam as CTM obtidas de tecido adiposo através da metodologia descrita por Rodbel e col,[56] muito antes da descoberta de seu potencial uso em engenharia de tecidos, na qual os tecidos são digeridos com colagenase e o fracionamento celular é feito por centrifugação e as células podem ser expandidas em frascos de cultura.

Muitas possíveis aplicações para o uso de CTM em engenharia de tecidos vem sendo estudadas,[57] mas uma descoberta fundamental foi a de que as CTM por si só tem um importante papel imunomoduladore por isso pode atuar orquestrando a atuação de outras células tanto inflamatórias como células maduras dos tecidos ao redor.[58-60] Dessa forma, é possível aventar a hipótese de que o tratamento de úlceras arteriais com células possa ter benefício duplo, com a diferenciação destas células em novos vasos sanguíneos como também no ajuste dos mecanismos imunológicos para otimização do reparo tecidual, mas os mecanismos fisiológicos envolvidos nesse processo ainda não estão completamente elucidados.

1.6 Estado atual da terapia celular para Doença Arterial Periférica e Isquemia Crítica

Tateishi-Yuyama e cols,[61] em um estudo pioneiro realizado com participantes portadores isquemia crítica (IC) de membro inferior, infundiu células mononucleares autólogas de medula óssea nos membros afetados, incluindo pacientes com dor em repouso, e obtiveram melhora significativa no índice de ITB (>0.09), aumento na árvore arterial observado pela comparação de angiografias pré e pós procedimento, aumento da oxigenação tecidual transcutânea (aumento de 13mmHg), e melhora de úlceras isquêmicas de extremidades em 60% dos participantes do grupo tratado.

Kawamura e cols,[62] em um estudo com uma série 92 participantes com ICM, injetaram células mononucleares autólogas obtidas de sangue periférico por estimulação medular com G-CSF nos membros, promovendo o aumento da temperatura e melhora dos sintomas. Compararam, na população do estudo, a presença de DMT2, necessidade de hemodiálise e a classificação Fontaine para DAP. Como resultado, observaram boa evolução das úlceras dos participantes em quase todos os grupos experimentais, sendo que não se observou amputação em quase todos os grupos experimentais que realizaram a terapia celular, exceto no grupo de pacientes isquêmicos classificados como Fontaine IV com DMT2 que estavam em hemodiálise.Este mesmo autor realizou um estudo mais amplo com 162 doentes nas mesmas condições e com período deobservação médio maior (26,4 meses) e concluiu que há diferença quando se trata portadores de doenças com diferentes etiologias, além do fato de se observar pior prognóstico para os participantes onde se obteve baixa contagem de células CD34 positivas e portadores de isquemia coronariana, sugerindo a necessidade demais estudos nessa linha de pesquisa.[63] (HORIE, 2010, p.461)

Em um estudo de revisão sistemática feito por Jiang e cols,[64] foi possível observar que apenas 14 estudos clínicos e randomizados controlados puderam ser compilados, sendo que na maioria foi realizado implante de CTM derivadas de medula óssea ou de sangue periférico após estimulação medular e nenhumfoi realizado com CTM obtidas e expandidas de tecido adiposo. A revisão pode observar que a terapia baseada em células-tronco autólogas foi associada a uma melhor cicatrização de úlceras de membros inferiores (sendo 12 comparações, 290 pacientes, RR = 3,07) com pouca heterogeneidade entre os dados. Concluiram que a terapia baseada em células-tronco autólogas é eficaz e segura para melhorar a cicatrizaçãode úlceras crônicas dos membros inferiores, sem nenhum efeito adverso maior relacionado a esse tipo de tratamento.

Recentemente, muitos cirurgiões plásticos estudaram o potencial de aplicação clínica das células- tronco derivadas do tecido adiposo (ASCs), para o tratamento de úlceras, já que estas células-tronco temfácil acesso e tem alto potencial de diferenciação celular, além de secretar fatores de crescimento que podemmelhorar os processos de cicatrização de feridas, promover a angiogênese e aumentar o suprimento de sangue local. Essa tendência têm sido estudada *in vitro* e *in vivo* em modelos animais, mas existem poucos ensaios clínicos randomizado sem humanos não havendo consenso sobre um protocolo clinicamente viável eque assegure resultados reproduzíveis.[65]

Dessa forma, o uso de CTM para o tratamento de úlceras arteriais pode ser uma alternativa viável, diminuindo as taxas de amputação de membros inferiores com baixo risco relativo ao tratamento. Entretanto,terapias com base na estimulação medular para obtenção de CTM de medula óssea ou de sangue periférico ainda não representam o cenário ideal para a terapia celular, pelos baixos, porém existentes, riscos relativos a necessidade de mobilização e de ampliação dessas células por fator estimulante de colônias granulocíticas (G-CSF).[66] A coleta e o uso de CTM derivadas de tecido adiposo é uma alternativa viável e considerada, por alguns pesquisadores, como a de melhor escolha, principalmente pelo seu bom rendimento em cultura, baixos riscos relacionados ao procedimento cirúrgico envolvido, facilidade de obtenção e plasticidade celular.[67]

2. JUSTIFICATIVA

De modo geral, o índice de amputação primária entre os portadores de DAP e ICM varia entre 10%a 40%, caracterizando um grande problema de saúde pública. Desse ponto em diante da doença, o tratamento clínico não promove melhoria para qualidade de vida do paciente, sendo necessário a realização de uma intervenção com revascularização cirúrgica para o membro acometido, sendo este, considerado o tratamento padrão para DAP e ICM. Apesar das várias técnicas disponíveis para revascularização de um membro, nem sempre isso será possível. Muitos doentes não apresentam condições clínicas para qualquer procedimento cirúrgico, alguns já esgotaram todas as formas de tratamento e outros, apesar da realização do melhor tratamento cirúrgico, ainda persistem em uma situação de ICM, com úlceras de difícil cicatrização, geralmente traumáticas ou decorrentes de amputações menores deixadas para cicatrização por segunda intensão. Nesses casos, a ocorrência de amputações maiores aumenta bruscamente.

Além, obviamente, da degradação final da qualidade de vida e da dor provocada neste estágio da doença, comparável apenas aos portadores de câncer terminal, as amputações acabam sendo a melhor

alternativa clínica disponível para esses doentes. As amputações geram um importante impacto socioeconômico, com perda da capacidade laboral (afastamento do trabalho), de socialização e, consequentemente, da qualidade de vida, constituindo-se como uma das mais devastadoras complicaçõesdas doenças degenerativas crônicas, associando-se à expressiva morbidade, incapacidade e mortalidade, não sendo, portanto, uma opção fácil para o doente, seus familiares e a própria equipe de saúde envolvida. Isso leva, frequentemente a um comprometimento dos familiares ou acompanhantes com os cuidados de um paciente que já não apresenta autonomia para os próprios cuidados, o que faz com que este se torne mais umproblema para a manutenção da estrutura familiar.

Em algumas situações, tanto pela não aceitação do paciente e seus familiares como pela inexistênciade um exame que possa predizer com certeza o melhor momento da decisão pela amputação, ou o contrário, posterga-se a amputação ou, ao contrário, realizam-se amputações em um momento onde se poderia ter uma conduta mais conservadora. Geralmente, os doentes amputados se tornam mais dependentes e há um agravamento geral da saúde, correlacionado diretamente com a perda de mobilidade. Com isso, há uma elevação dos gastos em saúde, especialmente pelo SUS, no caso do Brasil, considerando-se atendimentos ambulatoriais, atendimentos emergenciais, hospitalização, uso de analgésicos, antibióticos, curativos, entre outros.

Estudos clínicos com CTM autólogas ainda são incomuns e não foi possível encontrar na literatura científica o emprego de CTM com origem de tecido adiposo, expandidas em laboratório, utilizadas em injeções perilesionais em associação com aplicação na forma de um bicurativo estável personalizado, para o tratamento de doentes com DAP e ICM sem possibilidade de revascularização/revascularização incompletae portadores úlceras arteriais e risco eminente de perda do membro, quando na impossibilidade de revascularização cirúrgica, em todo o mundo.

Este ensaio clínico foi elaborado de forma bastante criteriosa para observar os efeitos do tratamento das úlceras arteriais com CTM autólogas de tecido adiposo expandidas em cultura. As mesmas serão aplicadas com injeção perilesional e também aplicadas com uso de curativo que possa ser mantido por 7 dias,a fim de proporcionar tempo suficiente para a migração celular auxiliando na cicatrização de úlceras arteriais em portadores de DAP e ICM. Serão avaliadas as taxas de cicatrização de feridas, salvamento de membros, seu possível efeito promotor de aumento da perfusão e, por fim, melhora da qualidade de vida para esses doentes. Assim, espera-se que as CTM exerção um papel imunomodulador orquestrando a atuação de outras células tanto inflamatórias como células maduras dos tecidos ao redor como também possam atuar em direção a diferenciação celular para promover a angiogênese como também diferenciarem- se em outros tipos celulares auxiliando na reparação tecidual (fibroblastos, queratinócitos e outras células da derme e epiderme).

3. OBJETIVOS

3.1 Objetivos Primário

Avaliar a segurança e a eficácia de injeções perilesionais de células tronco mesenquimais (CTM) adultas autólogas expandidas *in vitro* obtidas de tecido adiposo abdominal e diluídas em plasma autólogo

em associação com a aplicação das mesmas CTM na forma de um biocurativo estável, em uma única sessão de tratamento, para pacientes com doença arterial periférica (DAP) com isquemia crítica de membro (ICM), com úlcera arterial em membro inferior por tempo maior que 3 semanas, sem possibilidade de revascularização do membro, revascularização incompleta ou revascularização com reestenose ou oclusão, classificados como Fontaine IV e Rutherford 5 para DAP, avaliando-se comparativamente a ocorrência de amputações de membros e a cicatrização parcial ou total da úlcera, em comparação com o grupo que receberá curativos convencionais com Hidrogel com ácidos graxos essenciais (AGE).

3.2 Objetivos Secundários

- Verificar os efeitos do tratamento com injeções perilesionais e biocurativo estável com CTM adultasautólogas sobre:
- Perfusão do membro e tecidual;
- Qualidade de vida;
- Dor;
- Leito da úlcera;
- Parâmetros clínicos e fisiológicos
- Perfil inflamatório sistêmico
- Avaliação histológica da lesão
- Avaliar o nível de percepção de saúde dos participantes;
- Avaliar a taxa de salvamento de membro (não ocorrência de amputação)

4. DESENHO DO ESTUDO

4.1. Descrição do desenho do estudo

Estudo clínico, prospectivo, comparativo, randomizado, controlado e aberto com avaliação "cega" para os desfechos - *Prospective randomized open blinded endpoint* (PROBE).[68]

O estudo consiste de triagem/início (critérios de inclusão e exclusão, randomização) manuseio dasCTM, aplicação, acompanhamento do participante por 120 dias (visitas 2,3,4,5) e a visita final (6).

4.2. Justificativa para o desenho do estudo

É um ensaio clínico fase I/II, prospectivo, randomizado, controlado e aberto com avaliação "monocega" para os desfechos.

Foi delineado como um estudo clínico fase I/II porque não há possibilidade de realização de um estudo fase I, uma vez que os procedimentos necessários para sua realização em voluntários saudáveis (com pele íntegra) não são justificáveis do ponto de vista ético-profissional segundo a Declaração de Helsinki. Dessa forma, um número mínimo de participantes será alocado neste estudo, justificando-se resultados prévios obtidos em literatura científica e seguindo rigorosamente as normas de Boas Práticas em Células Humanas para uso terapêutico e pesquisa clínica, estabelecidas pelas resoluções RDCs Nº 214 de 2018 e Nº 260 de 2018 - ANVISA.[69, 70]

O estudo será aberto, mas os desfechos serão analisados por avaliadores independentes com julgamento dos desfechos de forma "cega". Não foi possível elaborar o ensaio clínico controlado por placebo e duplo cego, pois poderíamos infringir questões éticas pois teríamos que expor os voluntários, bastante debilitados, a um procedimento cirúrgico (retirada de amostra de tecido adiposo) desnecessário parao grupo placebo, além da aplicação de injeções e curativos placebo, que poderiam prejudicar ainda maisesse grupo de doentes. Acreditamos que o ensaio clínico proposto possa apresentar os resultados de forma clara, sem aumentar a exposição aos riscos causados por um grupo placebo, sendo este "cego" para os desfechos - Prospective randomized open blinded endpoint (PROBE).

4.3 Local do Estudo e ética

O estudo será executado no Hospital das Clínicas da Faculdade de Medicina de Botucatu. As CTMserão preparadas no Centro de Processamento Celular (CPC) do Laboratório de Biotecnologia Aplicada da Faculdade de Medicina de Botucatu – UNESP. O estudo está aprovado sobre o protocolo CEP/CONEP CAAE39873320.3.0000.5411

4.4 Cálculo amostral e tratamento estatístico dos resultados

4.4.1 Tamanho da Amostra e Recrutamento

A amostra foi estimada considerando o número de doentes atendidos anualmente no ambulatório de curativos vasculares do Hospital das Clínicas da Faculdade de Medicina de Botucatu – UNESP, no período de 20 meses com folga considerável e que se enquadrem nos critérios de elegibilidade do estudo. O recrutamento ocorrerá nas dependências do Hospital das Clínicas da Faculdade de Medicina de Botucatu - UNESP e na Unidade de Pesquisa Clínica da Faculdade de Medicina de Botucatu – UPECLIN- FMB/UNESP.

O cálculo amostral levou em consideração duas amostras independentes com proporção estimada na taxa de cicatrização das úlceras de 75% para o grupo 1, que será tratado com a terapia celular proposta neste estudo e 30% para o grupo 2, que será tratado com Hidrogel com AGE.

Prevendo-se um poder de teste de 80% e um nível de significância de 5% para um teste bilateral, a amostra fica definida como 20 participantes para o grupo 1 e 20 participantes para o grupo 2 (alocação 1:1). Prevendo que 5 participantes possivelmente não receberão o tratamento proposto após a inclusão, estesserão substituídos, totalizando uma amostra provável de 45 participantes no total. Este estudo foi desenhado para ser analisado por intenção de tratar (*intention to treat* – ITT). Os participantes serão de ambos os gêneros, com idade entre 18 a 90 anos.

Variáveis categóricas serão avaliadas pela média com o teste exato de Fisher, variáveis contínuas serão comparadas com métodos não paramétricos como teste de Mann-Whitney ou teste U, comparações pareadas serão avaliadas com o teste de Wilcoxon, análises multivariadas serão avaliadas com análise de regressão de Cox, análises clínicas de longo prazo serão compiladas em curvas de Kaplan-Meir.

4.4.2 Grupos proposto no ensaio clínicoGrupos de estudo

O estudo terá dois grupos de participantes que serão aleatorizados (conforme será melhor explicado no item Randomização):

Grupo 1 – Tratamento com Terapia Celular. Será composto por 20 participantes (n=20) que receberão o tratamento com CTM expandidas. O paciente comparecerá para realização de um procedimento de desbridamento cirúrgico da úlcera para que esta fique em sua melhor condição (Visita 1). Em sequência,o paciente receberá a aplicação de CTM por injeções perilesionais e um biocurativo produzido pela equipe do estudo também contendo as mesmas células (sessão única - o curativo contendo CTM ficará em contato com a úlcera por 7 dias). Após a retirada do curativo biológico, receberá cuidados locais da úlcera com curativo tópico de hidrogel (Curatec Hidrogel com AGE), gaze seca e faixa de crepe com troca no mínimo uma vez ao dia, conforme será melhor detalhado nos itens a seguir.

Grupo 2 – Grupo Controle. Será composto por 20 participantes (n=20) que receberão cuidados locais da úlcera. O paciente comparecerá para realização de um procedimento de desbridamento cirúrgico daúlcera para que esta fique em sua melhor condição (Visita 1). Em sequência, o paciente receberá a aplicação de curativo convencional com Hidrogel (Curatec Hidrogel com AGE), gaze seca e faixa de crepe. Será orientado a realizar a troca ao mínimo uma vez ao dia, conforme será melhor detalhado nos itens a seguir.

4.5 Critérios de Elegibilidade

Os critérios de elegibilidade foram definidos para selecionar participantes para os quais o tratamentoproposto for considerado adequado

4.5.1 Critérios de Inclusão

Tabela 2. Critérios de inclusão

**Descrição do critério de inclusão**	**Descrição do método de aferição docritério de inclusão**
Termo de consentimento livre e esclarecido assinado e datado	Concordar com todos os termos da pesquisa
Ambos os sexos, de qualquer origem étnica, com idade entre 18 e 90 anos	Ser maior de idade
Ausência de pulsos distais na perna (tibial anterior,pedioso e tibial posterior) e presença ou ausência de pulso poplíteo	Avaliação da ausência de pulsos pelaavaliação clínica
Doença Arterial Periférica	Comprovada por arteriografia ou por ultrassonografia vascular com Doplller;
Ter úlcera (s) no pé ou na perna (terço distal) de nomínimo 1cm^2 de área e máximo de 3 úlceras totalizando até 20cm^2 de área em membro inferior.	Avaliação clínica do membro inferior afetado
Ter recebido previamente tratamento com curativos convencionais para úlcera caracterizando que não houve melhora em um período mínimo de 3 semanas (antibioticoterapia – caso necessário – cuidados locais como desbridamentos mecânico, cirúrgicos ou químicos – caso necessário, curativos);	Avaliação clínica do membro inferior afetado em pelo menos duas consultas clínicas especializadas

DAP com IC classificada como Fontaine IV E Rutherford 5;	Apresentar úlcera arterial clinicamente comprovada em membro inferior afetado
Ter índice tornozelo braço (ITB) <0,9 nas artérias infrageniculares (tibial anterior, pediosa, tibial posterior e fíbular) ou ITB>1,3 em uma ou mais artérias infrageniculares quando portador de DMT2 crônico (tempo de doença > 5 anos) não submetidos a tratamento de revascularização ou submetidos ao tratamento com revascularização parcial ou falha do tratamento (estenose ou oclusão) nos últimos 12 meses.	Realização de ITB, avaliação clínica e de prontuário médico.
Impossibilidade de revascularização arterial do membro acometido (não apresentam artérias visíveis infrageniculares para abordagem cirúrgica) e ou revascularização incompleta desse membro (submetido a tratamento endovascular e ou cirúrgico que não foi capaz de restabelecer pulsos infrageniculares) e definido pelo médico que o melhor tratamento disponível do ponto de vista cirúrgico vascular já foi realizado e não se obteve sucesso em promover a cicatrização da úlcera;	Exame radiológico (ultrassonografia com Doppler, Arteriografia ou Angiotomografia) realizado em até 12 meses da avaliação clínica comprovando a impossibilidade de novas abordagens cirúrgicas para o membro acometido
Tratamento medicamentos cocomorbidades;	Constatação clínica do tratamento padrãocom antiagregantes plaquetários em uso pelo paciente no momento da inclusão etratamento das demais comorbidades.
Disponibilidade para comparecer nas consultas médicas;	Responder que apresenta interesse em comparecer às consultas médicas que serão agendadas durante a pesquisa.

Após a inclusão do doente no estudo, independentemente do grupo alocado, estes serão encaminhados para a coleta de exames laboratoriais: glicemia, hemoglobina glicada, colesterol total, HDL, LDL, triglicérides, hemograma completo, ácido úrico, uréia, creatinina, PCR, sódio e potássio, perfil de citocinas imunomoduladoras.

4.5.2 Critérios de Exclusão

Tabela 3. Critérios de exclusão

Descrição do critério de exclusão	Descrição do método de aferição docritério de exclusão
Estar grávida ou no puerpério	Confirmação diagnóstica de gravidez ou estar em puerpério.
Ter úlcera cicatrizada durante o período de triagem	Confirmação ao exame clínico que o participante teve as úlceras cicatrizadas durante a triagem.
Ter sinais de infecção sistêmica ou infecção ativa na úlcera arterial ou infecção de próteses cirúrgicas (bypass, pinos, parafusos). Poderão ser novamente elegíveis se submetidos à tratamento com antibióticos com sucesso e remoção do foco infeccioso.	Alteração em nível laboratorial (leucograma ou PCR) revelando sinais de infecção atividade sistêmica ou local. Avaliação clínica das características da úlcera arterial apresentando sinais de infecção ativa (rubor, secreção purulenta, calor local e crepitação).

Ter úlcera com tecido desvitalizado (necrose). Poderão ser novamente elegíveis se submetidos à desbridamento cirúrgico com sucesso ou amputações menores (amputação de artelhos ou limitadas ao antepé).	Avaliação clínica da úlcera arterial que comprove necrose tecidual.
Ter neoplasia e/ou estar em tratamento quimioterápico ou radioterápico ou em remissão há menos de 6 meses;	Avaliação de histórico clínico confirmando que se trata de paciente oncológico, em tratamento ou remissão há menos de 6 meses.
Estar em uso de colchicina ou imunomoduladores;	Histórico clínico de uso de colchicina ou imunomoduladores.
Ter doenças infectocontagiosas como Vírus da Imunodeficiência Humana (HIV/AIDS), Hepatites virais tipo B e C, Vírus Linfotrópico da Célula T Humana (HTLV); devido ao risco de contágio durante as manipulações de material biológico em laboratório.	Exames laboratoriais de comprovação diagnóstica.
Ter realizado amputação em nível de perna ou de coxa no membro que se pretende estudar.	Exame clínico comprovando amputação maior do membro que se pretende tratar.
Ter COVID-19, diagnósticado em tempo inferior há 4 semanas	Diagnóstico laboratorial por qRT-PCR. Após a negativação do exame, os pacientes podem tornar-se elegíveis para o estudo.

Os participantes excluídos no período de triagem, que corresponde ao momento entre a avaliação inicial e o momento de retorno para checagem dos exames de triagem e reavaliação da úlcera arterial, antes de receber o tratamento com terapia celular avançada, poderão ser substituídos por outro na mesma posição de randomização. Ainda nesta fase, os participantes poderão ser observados por um mês para melhoria das condições clínicas e iniciar os procedimentos efetivos do estudo. Após a coleta de tecido adiposo para obtenção das CTM autólogas expandidas, motivos de agravamento da saúde e da lesão podem ser fatores impeditivos para o participante receber o tratamento proposto neste estudo, sendo possível que o mesmo aguarde por até um mês para receber o tratamento do estudo. Caso o participante não receba o tratamento proposto no estudo, este será excluído e um novo participante será elegido para ocupar a posição daquele participante excluído, pois o estudo somente terá validade a partir do momento do efetivo tratamento do participante com a terapia celular avançada, sendo somente nestes casos é possível a avaliação por intenção de tratar (*Intention to treat* – ITT).

4.6 Randomização

Os participantes serão divididos em 2 grupos randomizados 1:1, aleatoriamente. Desta forma, receberão um número de inclusão sequencial a partir de uma tabela numérica sorteada eletronicamente e receberão o tratamento proposto.

Os participantes alocados no Grupo 1 - Terapia Celular - receberão o tratamento com a terapia celular proposta neste ensaio clínico (Injeções de CTM perilesionais em associação com biocurativo também contendo CTM) em uma sessão única de tratamento.

Participantes alocados no Grupo 2 - Controle - receberão tratamento com curativos de troca diária e seguirão a mesma agenda de visitas do primeiro grupo.

A randomização será elaborada por um técnico, utilizando programa de construção de tabelas numéricas aleatórias disponível em http://stattrek.com/Tables/Random.aspx. Serão sorteadas 20 posições, sendo fixado o número 1 para o Grupo 1 – terapia celular e o número 2 para o Grupo 2 – controle de forma aleatória e consecutiva. O envelope contendo a randomização estará de posse de um profissional independente, de forma que os membros da equipe do estudo não saberão a ordem de alocação até o momento da coleta do tecido adiposo.

4.6.1 Metodologia e garantia de qualidade de resultados no estudo aberto

Trata-se de um estudo clínico, prospectivo, unicêntrico, controlado, randomizado, aberto com avaliador cego para os desfechos (PROBE – *prospective, randomized, open, blinded endpoint* – estudo prospectivo, randomizado, aberto, com resultado cego).[66]

Os avaliadores serão membros externos, não pertencentes à equipe de execução do estudo e receberão eletronicamente as imagens das úlceras identificadas somente com o número do participante e visita e um formulário eletrônico com as variáveis já descritas para avaliação da úlcera. Após o término do estudo, os dados serão encaminhados a um estatístico independente para análise.

4.7 Procedimentos do estudo clínico nos grupos propostos

4.7.1 Coleta de Tecido Adiposo para obtenção de células-tronco mesenquimais autólogas para uso noGrupo 1

Após a inclusão e randomização do participante para o grupo 1 este retornará em um prazo de 7 diaspara checagem de exames laboratoriais (ver mais detalhes no item 4.16). Será agendada uma cirurgia ambulatorial, com anestesia local, para a coleta de gordura abdominal autóloga (amostra de tecido adiposo pesando 5g a 10g) o mais brevemente possível (estimativa de até 7 dias).

A coleta cirúrgica ambulatorial seguirá todas as normas de técnicas de procedimentos cirúrgicos: 1. assepsia; 2. colocação de campos estéreis; 3. anestesia local com Lidocaína 2% sem vasoconstritor não ultrapassando a dose máxima de 4,5mg/kg, na região ínfero lateral abdominal direito ou esquerdo; 4. proceder-se-á uma incisão elíptica em fuso orientada para as linhas de força da pele abdominal com aproximadamente 3cm de comprimento e 1,5cm de largura, aprofundando-se até o limite com a fáscia muscular, sem utilização de cauterização elétrica. A hemostasia será feita com cauterização elétrica se necessário, a incisão será suturada com pontos separados com Vicryl 4-0 para o subcutâneo, a pele será suturada com pontos simples de NYLON 4-0. Um curativo estéril com gaze de algodão e Micropore® será aplicado sobre o local.

O tecido obtido será colocado em tubo plástico cônico, estéril de 50ml contendo 30 ml de meio de transporte HEPES e este será transportado em condição refrigerada entre 4ºC e 8ºC para o CPC do Hemocentro do Hospital das Clínicas da Faculdade de Medicina de Botucatu (FMB) - UNESP.

4.7.2 Processamento da amostra de Tecido Adiposo

A amostra de tecido adiposo será processada no CPC sendo codificada com uma sequência numérica para identificação e armazenamento de material humano, com registro eletrônico, de forma a proporcionar o fornecimento seguro de CTM autólogas para o participante.

O tecido adiposo será manipulado em cabine de fluxo laminar de forma asséptica. Inicialmente, serálavado com soro fisiológico 0,9% e colocado em um novo frasco cônico de plástico estéril de 50ml de meio HEPES acrescido de ciprofloxacino 10% (32 µg/mL) e anfotericina B 50µg/ml para evitar a proliferação de bactérias e fungos, mantido nesta solução em geladeira própria à 4°C por 24 horas para quarentena de descontaminação.

Após este período, o tecido adiposo será submetido à dissociação enzimática, onde o tecido será fragmentado com o intuito de aumentar a superfície de ação da enzima e será adicionado 4mg colagenase tipo I em 2ml de Xeno free aditivado (MesenCult®, Stem Cell™) para a proporção de 1g de tecido adiposo. Essa solução contendo o tecido adiposo será armazenado em ambiente de estufa por 15h para que a enzima dissolva a matriz de colágeno e libere as células. Nesse momento, o mesmo volume de Xeno free aditivado (MesenCult®, Stem Cell™) será adicionado para inativação da ação enzimática da colagenase tipo I. Esta solução será submetida a centrifugação a 1200 rpm durante 10 minutos. O sobrenadante será desprezado e o *pellet* obtido será submetido a 3 lavagens com ressuspensão em meio HEPES para eliminação dos debris celulares, de gordura liquefeita, da matriz extracelular e de células sanguíneas. O *pellet* obtido no final desseprocesso de lavagem será suspenso em 1ml de meio de cultura Xeno free aditivado (MesenCult Stem Cell®)para a semeadura. Uma alíquota de 30uL será coletada para contagem celular em câmara de Neubauer para verificação da viabilidade celular e quantificação do número de células totais viáveis (protocolo de controle de qualidade inicial). Na sequência, as células viáveis obtidas serão plaqueadas em frascos de cultura de 25cm^2 na proporção aproximada de 2.000 a 3000 CTM/cm^2 e então será acrescentado 4mL de meio de cultura Xeno free aditivado (MesenCult®, Stem Cell™) por frasco para dar seguimento à cultura.
Todas as informações obtidas durante o processamento celular serão registradas eletronicamente.

4.7.3 Procedimentos de Expansão Celular

Os frascos de cultura de 25cm^2 obtidos após o processamento da amostra serão colocados em estufa úmida à 37ºC, com 5% de CO_2 (Thermo Class 100®) até que se observe uma confluência superior a 80% daárea do fundo da placa, através de monitoramento direto por microscopia invertida. O meio de cultura será trocado a cada 48h. Após atingir a confluência de 80% da área da placa, será realizado o procedimento de ampliação do número de frascos, de forma a não inibir o crescimento celular, (competição por nutrientes e espaço) momento este chamado de passagem. O procedimento de passagem será feito com o descolamento das células do frasco de cultura adicionando 0,1mL de tripsina (Stem Cell™) para cada cm^2 de área do frasco, mantidos por 5 minutos em estufa a 37ºC. Após esse tempo, será adicionado igual volume de meio

de cultura Xeno free aditivado (MesenCult®, Stem Cell™) para neutralização da tripsina. Esse volume contendo as células será centrifugado e ressuspendido duas vezes e as células obtidas serão contadas e replaqueadas em frascos de 75cm^2 (métodos descritos em detalhes na sessão anterior). As passagens serão repetidas até que se atinja o número de células necessárias para os procedimentos de aplicação e para os controles de qualidade (ver mais detalhes a seguir no item controle de qualidade celular 4.11). Espera-se queo número de células seja atingido em 3 ou 4 passagens (pelo menos 1X10^7 CTM, com tempo estimado em21 a 28 dias do processamento inicial). Todas as informações obtidas do processamento celular serão registradas eletronicamente.

4.7.4 Controle de qualidade celular

A preparação do produto a ser empregado seguirá as normas da ANVISA para segurança de materialbiológico, especificamente as resoluções RDC 214/2018 e RDC 260/2018.[67, 68] Todas as amostras celulares serão catalogadas eletronicamente em um banco de dados seguro e serão destinadas exclusivamente ao participante do estudo (autóloga), utilizando-se código de barras nos tubos para identificação.

4.7.5 Controle microbiológico da cultura celular

Para o controle de qualidade celular, no que tange a infecção por fungos e bactérias uma amostra de 1mL de meio de cultura dos frascos contendo células em cada passagem será acondicionado em frasco tipo Bactec® e encaminhado ao laboratório de microbiologia para análise. Em caso de contaminação, o frasco de cultura será descartado. Para análise de presença de micoplasma, uma amostra de 1x10^6 CTM de primeira passagem será enviada ao laboratório de Biologia Molecular. Em caso de confirmação de infecção por micoplasma o laboratório deverá suspender as atividades e todas as culturas serão descartadas e o laboratório será submetido aos procedimentos de descontaminação ambiental.

Todas as culturas serão monitoradas três vezes por semana por microscopia ótica invertida direta, para verificação da confluência celular e prevenção de eventuais contaminações com fungos e bactérias.

4.7.6 Controle de qualidade das célula-tronco

A análise para caracterização imunofenotípica das CTM será realizada por citometria de fluxo, de forma que, as células obtidas serão melhor caracterizadas como CTM. Para isto, serão realizadas análises de um painel mínimo de 3 marcadores positivos (dentre esses: anti-CD44, anti-CD73, anti-CD90 ou anti- CD105) e 3 marcadores negativos (dentre esses: anti-CD11b, anti-CD14, anti-CD19, anti-CD31, anti-CD34,anti-CD41, anti-CD45 ou anti-CD79α). Uma amostra de 1x10^6 CTM de terceira passagem, em forma de *pellet,* será encaminhada ao laboratório de citometria fluxo, que deverá ser processada em um prazo máximode 2 horas.

A análise de viabilidade celular será feita por citometria de fluxo através da determinação do índice de apoptose/necrose usando kit de anexina V, iodeto de propídio e Caspase-3. Para isso, uma amostra de 1x10^6 CTM de terceira passagem, em forma de *pellet,* será encaminhada ao laboratório de citometria fluxo, que deverá ser processada em um prazo máximo de 2 horas.

A análise da diferenciação celular será realizada como um teste adicional para garantir que as CTM são capazes de se diferenciar em outros tipos celulares. Uma amostra de 3x10^6 células de terceira passagem

serão plaqueadas em 3 frascos de cultura de 25cm^2 e serão submetidas ao protocolo de diferenciação em tri- linhagem (3 linhagens celulares diferentes - adipócitos, condrócitos e osteócitos) através do uso do kit apropriado para diferenciação celular *StemPro adipogenesis*, *chondrogenesis*, *and osteogenesis kits*, Invitrogen®, segundo diretrizes da *International Society for Cellular Therapy* (ISCT).

4.7.7 Avaliação de expressão gênica por RT-qPCR

Uma amostra de 1x10^6 CTM de terceira passagem será enviada ao Laboratório de Biologia Molecular para proceder testes genéticos através da técnica de reação em cadeia da polimerase em temporeal após transcrição reversa (RT-qPCR) para um painel de ao menos 20 genes, incluindo genes relacionadosa indiferenciação celular (Nanog, c-Myc, Oct4, Sox2 e Klf4), gene relacionado a característica da linhagem adipogênica (PPARG), genes supressores tumorais (APC, WT-1, RB, p53, NF-1 e DCC), genes relacionadosa receptores de fatores de crescimento (ERBa, ERBb e p28^{sis}), gnenes relacionados a proteínas reguladoras de transcrição (p110myc, p55fos, p48myc) e de identificação de alguns oncogenes que podem apresentar-se em desarranjo, para prevenção de neoplasias (C-myc, N-myc, L-myc, C-erb, Neu/erb-B2).

Serão seguidas as orientações do *Minimum* information for Publication of Quantitative Real-Time PCR Experiment (MIQE).[71] Inicialmente o ácido ribonucleico (RNA) total de CTM serão extraídas utilizando Trizol (Life Technologies, EUA), conforme orientações do fabricante. As amostras de RNA serãotratadas com TURBO DNase (Ambion, EUA) para remoção de contaminação com DNA genômico. Em seguida, o RNA será quantificado por espectrofotometria utilizando o equipamento NanoDrop (ThermoFisher, EUA). A qualidade do RNA extraído será determinada pelo número da integridade do RNA (RNA Integrity Number, RIN) a partir da análise dos RNAs ribossomais utilizando-se o sistema 2100 Bioanalyzer (Agilent, EUA).[72] Após a avaliação da qualidade do RNA será realizada a transcrição reversa para mRNA. Para tal, será utilizado o kit High Capacity RNA-to-cDNA Master Mix (Life Technologies, EUA), seguindo as orientações do fabricante para um volume total de 20µL de reação. As condições de termociclagem serão as mesmas indicadas pelo fabricante. Na sequência será realizado PCR quantitativo emTempo Real (qPCR). Serão confeccionadas utilizando SYBRgree Universal PCR Master Mix, No AmpErase® UNG por duas vezes (Life Technologies, EUA) para um volume total de reação de 20µL, conforme recomendações do fabricante. Serão utilizados primers específicos para os transcritos (mRNA)dos genes alvo. Para construção dos primers será utilizado o Primer Blast,[72] preconizando flanquear regiões entre exons. A quantificação relativa da expressão gênica será realizada utilizando o método de Cq comparativo.[73]

Tabela 4. Padrão de conformidade para células-tronco mesenquimais

Teste	Resultado	Conformidade
Contaminação à microscópia invertida	Ausente	Conforme
Microbiologia	Aeróbio: Negativo<br>Anaeróbio: Negativo<br>Fungos: Negativo<br>Micoplasma: Negativo	Conforme
Diferenciação Celular	Adipogênica: Positivo para Oil Red<br>Condrogênica: Positivo azul de toluidina<br>Osteogênica:Positivo para Alizarin Red	Conforme
Imunofenotipagem	CD44, CD73, CD90, CD105: Positivo >90%<br>CD11b, CD14, CD19, CD31, CD 34, CD45, CD79α: Negativo < 5%	Conforme

Anexina V / Iodeto de propídio /Caspase-3	Índice de Apoptose/ Necrose < 5%	Conforme
Biologia Molecular	Identificação de expressão de genes de indiferenciação Funcionalidade: positivo<br><br>ConformeIdentificação de expressão de genes tumorais: negativo	Conforme

4.8 Justificativa para quantidade de células (Dose)

Com base nos poucos estudos encontrados na literatura científica em que a terapia celular foi empregada para o tratamento de DAP com IC, sem perspectivas de tratamento com revascularização, foi observada grande variabilidade metodológica na forma de aplicação e "dose". As principais variáveis encontradas foram em relação à quantidade de CTM que devem ser aplicadas. Geralmente utiliza-se como base de cálculo a superfície de área da úlcera. Em estudos que foram utilizados injeções intrarteriais ou periarteriais, os cálculos foram baseados na extensão dos vasos. Nos estudos onde foi feita a injeção intramuscular levou-se em consideração o volume muscular. Por outro lado, o número de células cultivadase a garantia de qualidade das CTM foi dependente de vários fatores como: o tempo de cultura, o número de passagens em cultura, a idade do participante, o sítio de obtenção (sangue periférico ou medula óssea) entre outros.

Então, de forma que, para padronizarmos o método que será empregado possibilitando sua repetição,este estudo vai utilizar número de células proporcional à área da úlcera (dose). Dessa forma, cada punção aplicará 0,1ml de solução (plasma autólogo) contendo 10.000 CTM ($1X10^4$ CTM/0,1ml de solução). A injeção contendo a solução (plasma + CTM) será aplicada no tecido subcutâneo da borda da lesão, a cada um cm, de forma a circundar a úlcera.

Para aumentar as chances de epitelização, as CTM também serão aplicadas de forma tópica. Para isso, um curativo biológico e bioabsorvível, que será produzido para este estudo, vai adicionar 10.000 CTM para cada cm^2 de úlcera ($1X10^4$ CTM/cm^2) que será coberto secundariamente com gaze e faixa crepom (ver mais detalhes a seguir em Curativo com CTM).

4.9 Preparação, armazenamento e procedimentos para dispensação do produto

Assim que se obtiver a contagem celular em cultura para que se possa fazer o uso clínico no participante, a equipe do CPC acionará a equipe do estudo para convocação do participante e a realização dotratamento proposto (Grupo 1 – Terapia celular) em um período máximo de 7 dias.

Na impossibilidade de comparecimento ou presença de infecção ativa na úlcera no dia do comparecimento, as CTM serão criopreservadas em nitrogênio líquido à 196ºC nas dependências do CPC. O participante do estudo ainda poderá receber a terapia com suas células caso apresente-se apto em um período máximo de 1 mês.

Uma amostra de $1X10^6$ CTM de cada participante do Grupo 1 serão criopreservadas em freezer à80ºC nas dependências do CPC com registro celular para possíveis análises futuras, se necessário e como estoque de garantia caso haja contaminação da cultura em estágio de amplificação. Estas células serão armazenadas durante o período do estudo e depois serão descartadas em lixo apropriado para incineração.

Para o grupo 1, que receberá o tratamento com terapia celular avançada, com 20 participantes, o produto teste será apresentado de duas formas que serão aplicadas conjuntamente: na forma de biocurativo estável, este será

constituído de uma lâmina fina (3mm de espessura) de Fibracol™ Plus (colágeno 90% + Alginato de Cálcio 10%) que será embebido em uma solução contendo plasma autólogo fresco e CTM do participante, de forma personalizada, onde o curativo será produzido de forma a promover a cobertura da área da úlcera, sendo que uma proporção de $1x10^4$ CTM serão disponibilizadas para cada cm^2 de curativo. Durante a produção do curativo, a solução de plasma contendo as CTM será induzida a “gelificar” (ganhar consistência para não escorrer) com a adição de Trombina 800–1200UI/mL na proporção de 1/10mL da solução de plasma a ser aplicado + Gluconato de Cálcio 10% na mesma proporção que a Trombina. O curativo pronto será acondicionado em placas de Petri e lacrados. Uma embalagem secundária estéril de plástico isolará o produto. O produto deverá ser transportado em uma caixa de isopor refrigerada (entre 2 e 8°C), lacrada e com a identificação do participante da pesquisa; na forma de solução para injeção perilesional, plasma autólogo fresco será utilizado para diluição de $1X10^5$ CTM para cada 1mL da solução e será disponibilizado em seringas de 1mL já com agulha hipodérmica 27G. A aplicação será de 0,1mL para cada centímetro de borda da lesão. As seringas contendo a solução de plasma fresco e CTM serão produzidas na proporção que estima-se que serão aplicadas naquele participante. As seringas preparadas serão acondicionadas em uma embalagem estéril de plástico, que isolará o produto. Este produto deverá ser transportado em uma caixa de isopor refrigerada (entre 2°C e 8°C), lacrada e com a identificação do participante da pesquisa, sendo o mesmo isopor que transportará o curativo.

Após o produto devidamente embalado, este deve ser aplicado no participante em prazo máximo de 4 horas. A embalagem será transportada pelo responsável pela distribuição deste produto até o local onde será aplicado (centro cirúrgico).

Já os participantes do grupo 2, controle, com 20 participantes, receberão cuidados locais da úlcera, sendo que o participante comparecerá para realização de um procedimento de revitalização da úlcera, para que esta fique em condições de equidade com o grupo 1, o que será realizado na Visita 1. Em sequência, o participante receberá a aplicação de curativo convencional com Hidrogel (Curatec Hidrogel com AGE), gaze seca e faixa de crepe. Será orientado a realizar a troca ao mínimo uma vez ao dia e seguirá o mesmo cronograma de consultas que os participantes do grupo 1.

4.10 Procedimento de aplicação do produto

A posologia prevista para este ensaio clínico será a de uma aplicação em dose única do produto, queserá disponibilizado para uso através de duas vias de administração: biocurativo estável para aplicaçãotópica sobre a úlcera arterial e solução injetável para aplicação hipodérmica perilesional.

O procedimento de aplicação do produto de terapia celular avançada proposta neste ensaio clínico será realizado após anestesia locorregional (preferencial) ou geral, a critério do anestesiologista que realizará o procedimento, levando-se em consideração as condições clínicas do participante, uma vez que o procedimento não pode ser realizado sem a devida analgesia. A aplicação das injeções e do curativo seguirá as normas de procedimentos cirúrgicos, realizando-se a antissepsia, colocação de campos estéreis, materiais cirúrgicos de forma estéril e abertura dos envelopes contendo os produtos a serem aplicados de forma estéril.Uma limpeza e um desbridamento cirúrgico iniciais serão realizados na ferida de forma a torná-la apta areceber o tratamento. Serão, então, procedidas as aplicações subcutâneas de 0,1mL do produto que consiste na solução de plasma e CTM autólogas expandidas a cada 1 cm da borda da úlcera (no subcutâneo da pele íntegra no perímetro da lesão), de forma que aproximadamente $1x10^4$ CTM sejam injetadas por punção. Após as injeções, será procedida a aplicação tópica do biocurativo estável contendo as CTM “gelificadas”

na membrana de Fibracol™ sobre a área da úlcera arterial. Em seguida, o curativo será coberto com uma gaze de rayon (identificando o término do curativo primário), depois será colocado o curativo secundário com uma fina camada de gazes umidificadas com soro fisiológico 0,9% e outra camada com gaze seca e, por fim, a faixa crepe. Somente o curativo secundário será trocado diariamente pelo participante ou seus cuidadores, conforme orientações que serão dadas pela equipe do estudo. O participante receberá alta assim que estiver clinicamente estável. Após 7 dias, retornará para a reavaliação da úlcera, sendo realizada a troca do curativo primário e secundário pela equipe do estudo, passando então, para a fase onde realizará apenas curativos com Hidrogel com AGE (a faze de absorção das CTM já foi completada).

4.11 Controle de qualidade do estudo

4.11.1 Procedimentos de amostragem

- **Obtenção de tecido adiposo:** a amostra de tecido adiposo será mantida estéril tanto pelo processo cirúrgico como pelo acondicionamento em tubo cônico estéril contendo HEPES . O transporte será feito em caixa de isopor refrigerada com temperatura controlada entre 2 e 8°C, lacrada e com a identificação do participante da pesquisa. O tecido adiposo será manipulado no CPC em prazo máximo de 2 horas, em capela de fluxo laminar. Será lavado 3 vezes com solução fisiológica 0,9% etrocado de tubo, identificado para o participante do estudo, agora contendo HEPES (xenofree - STEMCELL™ Technologies) acrescido de ciprofloxacino 10% (32 µg/mL) e anfotericina B 50µg/ml. Será mantido nesta solução em câmara fria, específica para essa finalidade, à 4°C por 24 horas para quarentena de descontaminação.
- **Obtenção de CTM para expansão:** Após a quarentena, a amostra de tecido adiposo será submetidaa dissociação enzimática por 15h. A solução dissociada será submetida a centrifugação a 600xg durante 10 minutos. O sobrenadante será desprezado e o *pellet* obtido será submetido a 3 lavagens com ressuspensão em meio HEPES (xenofree - STEMCELL™ Technologies) para eliminação dos debrís celulares, gordura liquefeita, matriz extracelular e células sanguíneas. O *pellet* obtido no finaldesse processo de lavagem será suspenso em 1ml de meio de cultura (MensenCult-S - xenofree - STEMCELL™ Technologies). Uma alíquota de 30uL será coletada para contagem celular em câmara de Neubauer para verificação da viabilidade celular e quantificação do número de células totais viáveis (protocolo de controle de qualidade inicial). As células serão plaqueadas em frascos decultura de 25cm^2 para dar seguimento à cultura.

- **Controle de expansão celular:** Os frascos de cultura de $25cm^2$ obtidos após o processamento da amostra serão colocados em estufa úmida à 37°C, com 5% de CO2 (Thermo Class 100®) até que se observe uma confluência superior a 80% da área do fundo da placa, através de monitoramentodireto por microscopia invertida. O meio de cultura será trocado a cada 48h, sempre de maneira estéril em fluxo laminar. Se observadas alterações no frasco de expansão celular que sugiram contaminação leve, o sobrenadante será enviado para análise microbiológica e o frasco de cultura será descartado. Em observação de contaminação externa do frasco de cultura, estes serão descontaminados com seguindo protocolo do laboratório e todos os frascos de cultura da estufa serão observados em microscopia invertida para identificação de contaminação interna, sendo os positivos descartados. A estufa será desligada e descontaminada segundo protocolo do laboratório.O procedimento de passagem celular será monitorado conforme descrito abaixo. Todas as informações obtidas do processamento celular serão registradas eletronicamente, incluindo contagem celular e viabilidade celular. Todos os frascos sempre serão identificados para o participante.
- **Controle microbiológico da cultura celular (fungos e bactérias):** uma amostra de 1mL de meio de cultura dos frascos contendo células em cada passagem será acondicionado em frasco tipo Bac- tec® e encaminhado ao laboratório de microbiologia para análise. Em caso de contaminação, o fras-co de cultura será descartado.
- **Controle microbiológico da cultura celular (micoplasma):** uma amostra de $1x10^6$ CTM de pri- meira passagem será enviada ao laboratório de Biologia Molecular. Em caso de confirmação de in- fecção por micoplasma o laboratório deverá suspender as atividades e todas as culturas serão des- cartadas e o laboratório será submetido aos procedimentos de descontaminação ambienta
- **Controle de qualidade das CTM (caracterização imunofenótipica**): Uma amostra de $1x10^6$ CTMde terceira passagem, em forma de pellet, será encaminhada ao laboratório de citometria fluxo.
- **Controle de qualidade das CTM (viabilidade celular):** uma amostra de $1x10^6$ CTM de terceira passagem, em forma de pellet, será encaminhada ao laboratório de citometria fluxo para realizarteste de determinação do índice de apoptose/necrose usando kit de anexina V, iodeto de propídio e Caspase-3.
- **Controle de qualidade das CTM (diferenciação celular):** uma amostra de $3x10^6$ células de terceira passagem serão plaqueadas em 3 frascos de cultura de $25cm^2$ e serão submetidas ao protocolo de diferenciação em tri-linhagem (3 linhagens celulares diferentes - adipócitos, condrócitos e osteócitos).
- **Controle de qualidade das CTM (Avaliação de expressão gênica por RT-qPCR):** uma amostra de $1x10^6$ CTM de terceira passagem será enviada ao Laboratório de Biologia Molecular para proceder testes genéticos através da técnica de reação em cadeia da polimerase em tempo real após transcrição reversa (RT-qPCR).

- **Congelamento de amostra:** uma amostra de 1x10^6 CTM de cada participante do grupo que receberá o tratamento com terapia celular avançada serão criopreservadas em freezer à -80°C para possíveis análises futuras durante o perído do estudo.
- **Dose da terapia celular :** será caracterizada por contagem automática de amostra de 30uL da solução de 1mL contendo as células-tronco.

4.11.2 Requisitos para aceitação de lote de materiais, reagentes e produtos para diagnóstico *in vitro* utilizados na manufatura do produto e nos processos de controle de qualidade

Todos os meios de cultura, reagentes e suplementos que serão utilizados em cultura celular deverão ter controle de qualidade xenofree e esterilidade garantidos pelo fabricante, além de identificação de lote de fabricação e origem na embalagem para rastreabilidade do produto; prazo de validade superior a 6 meses, identificação de concentração dos seus componentes químicos e embalagem não violada. Todos os materiais utilizados em cultura celular serão de uso único, descartados após o uso, adquiridos de forma estéril direto do fabricante, com identificação de lote de fabricação e origem na embalagem para rastreabilidade do produto; prazo de validade superior a 6 meses, identificação seus componentes químicos eembalagem não violada. Kits diagnósticos devem apresentar identificação de lote de fabricação e origem na embalagem para rastreabilidade do produto; prazo de validade superior a 6 meses, identificação dos seus componentes químicos e embalagem não violada. Todos os procedimentos serão realizados por procedimentos operacionais padrão e estarão de acordo com a RDC 214/2018 e RDC 260/2018.

Para controle de qualidade interno, após a abertura dos frascos de meio de culturas e aditivos, e/ou outros produtos, os mesmos serão conservados conforme descrito no manual de uso dos fabricantes (em câmeras frias de 2°C a 8°C e/ou freezer -20°C e/ou freezer -80°C). Os mesmos serão submetidos a testes microbiológicos sempre que forem observadas alterações de turbidez e/ou observado formação de grânulos ou mudanças de coloração ou outras mudanças sugeridas pelo fabricante e descartados. Todos os produtos serão aliquotadas por participante e os frascos serão identificados, de forma que nenhum produto que possa apresentado contato ambiental com células de um participante possa ser colocado em contato com outro participante.

4.11.2 Requisitos de segurança e qualidade para liberação do produto de terapia avançadainvestigacional para administração

- **Controle de qualidade**

  A preparação do produto de terapia celular avançado deste estudo seguirá as normas da ANVISA para segurança de material biológico, especificamente as resoluções RDC 214/2018 e RDC 260/2018. Todasas amostras celulares serão catalogadas eletronicamente em um banco de dados seguro e serão destinadas exclusivamente ao participante do estudo (autóloga), utilizando-se código de barras nos tubos para identificação.

- **Controle microbiológico da cultura celular**

  Para o controle de qualidade celular, no que tange a infecção por fungos e bactérias uma amostra de 1mL de meio de cultura dos frascos contendo células em cada passagem será acondicionado em frasco tipo

Bactec®e encaminhadoao laboratório de microbiologia para análise. Em caso de contaminação, o frasco de cultura será descartado. Para análise de presença de micoplasma, uma amostra de $1x10^6$ CTM de primeira passagem será enviada ao laboratório de Biologia Molecular. Em caso de confirmação de infecção por micoplasma o laboratório deverá suspender as atividades e todas as culturas serão descartadas e o laboratório será submetido aos procedimentos de descontaminação ambiental.Todas as culturas serão monitoradas três vezes por semana por microscopia ótica invertida direta, para verificação da confluência celular e prevenção de eventuais contaminações com fungos e bactérias.

- **Controle de qualidade das célula-tronco**

A análise para caracterização imunofenotípica das células tronco será realizada por citometria de fluxo, de forma que, as células obtidas serão melhor caracterizadas como células-tronco. Para isto, serão realizadas análises de um painel mínimo de 3 marcadores positivos (dentre esses: anti-CD44, anti-CD73, anti-CD90 ou anti-CD105) e 3 marcadores negativos (dentre esses: anti-CD11b, anti-CD14, anti-CD19, anti-CD31, anti-CD34, anti-CD41, anti-CD45 ou anti-CD79α). Uma amostra de $1x10^6$ CTM de terceira passagem, em forma de pellet, será encaminhada ao laboratório de citometria fluxo, que deverá ser processada em um prazo máximo de 2 horas. A análise de viabilidade celular será feita por citometria de fluxo através da determinação do índice de apoptose/necrose usando kit de anexina V, iodeto de propídio e Caspase-3. Para isso, uma amostra de $1x10^6$ CTM de terceira passagem, em forma de pellet, será encaminhada ao laboratório de citometria fluxo, que deverá ser processada em um prazo máximo de 2 horas. A análise da diferenciação celular será realizada como um teste adicional para garantir que as células-tronco são capazes de se diferenciar em outros tipos celulares Uma amostra de $1x10^6$ células de terceira passagem será plaqueada para cada um dos 3 frascos de cultura de $25cm^2$ que serão submetidas ao protocolo de diferenciação em tri-linhagem (3 linhagens celulares diferentes - adipócitos, condrócitos e osteócitos) através do uso do kit apropriado para diferenciação celular StemPro adipogenesis, chondrogenesis, and osteogenesis kits, Invitrogen. Os resultados positivos para a diferenciação servirão como controle de qualidade celular. Será realizado, ainda, um teste de controle de qualidade celular por Biologia Molecular. Uma amostra de $1x10^6$ CTM de terceira passagem será enviada ao Laboratório de Biologia Molecular para proceder a análise através de um painel de ao menos 20 genes com o intuito de garantir sua funcionalidade celular e sua qualidade não tumoral. Tais análises são importantes para garantir o padrão mínimo de quali- dade para as células-tronco sendo assim aptas para uso terapêutico em seres humanos, segundo as diretrizes da International Society for Cellular Therapy (ISCT).

- **Avaliação de expressão gênica por RT-qPCR:**

Uma amostra de $1x10^6$ CTM de terceira passagem será enviada ao Laboratório de Biologia Molecular da mesma Instituição, para que sejam realizados testes genéticos através da técnica de reação em cadeia da polimerase em tempo real após transcrição reversa (RT-qPCR) para um painel de ao menos 20 genes, incluindo genes relacionados a indiferenciação celular (Nanog, c-Myc, Oct4, Sox2 e Klf4), gene relacionado a característica da linhagem adipogênica (PPARG), genes supressores tumorais (APC, WT-1, RB, p53, NF-1 e DCC), genes relacionados a receptores de fatores de crescimento (ERBa, ERBb e p28sis), gnenes relacionados a proteínas reguladoras de transcrição (p110myc, p55fos, p48myc) e de identificação de

alguns oncogenes que podem apresentar-se em desarranjo, para prevenção de neoplasias (C-myc, N-myc, L-myc, C-erb, Neu/erb-B2). Serão seguidas as orientações do *Minimum Information for Publication of Quantitative Real-Time PCR Experiment* (MIQE). Inicialmente o ácido ribonucleico (RNA) total de CTM será extraído utilizando Trizol (Life Technologies, EUA), conforme orientações do fabricante. As amostras de RNA serão tratadas com TURBO DNase (Ambion, EUA) para remoção de contaminação com DNA genômico. Em seguida, o RNA será quantificado por espectrofotometria utilizando o equipamento NanoDrop (ThermoFisher, EUA). A qualidade do RNA extraído será determinada pelo número da integridade do RNA (RNA Integrity Number, RIN) a partir da análise dos RNAs ribossomais utilizando-se osistema 2100 Bioanalyzer (Agilent, EUA). Após a avaliação da qualidade do RNA será realizada a transcrição reversa para captura dos mRNAs. Para tal, será utilizado o kit High Capacity RNA-to-cDNA Master Mix (Life Technologies, EUA), seguindo as orientações do fabricante para um volume total de 20µLde reação. As condições de termociclagem serão as mesmas indicadas pelo fabricante. Na sequência será realizado PCR quantitativo em Tempo Real (qPCR). Serão confeccionadas utilizando SYBRgree Universal PCR Master Mix, No AmpErase® UNG por duas vezes (Life Technologies, EUA) para um volume total de reação de 20µL, conforme recomendações do fabricante. Serão utilizados primers específicos para os transcritos (mRNA) dos genes alvo. Para construção dos primers será utilizado o Primer Blast (YE, 2012, p.134), preconizando flanquear regiões entre exons. A quantificação relativa da expressão gênica será realizada utilizando o método de Cq comparativo.

4.11.3 Caracterização do componente ativo, incluindo, quando couber, sua identidade, quantidade, pureza, viabilidade, potência, estabilidade genética e esterilidade, devendo descrever as metodologias analíticas empregadas

O componente ativo produto de terapia celular avançada deste projeto trata-se de células-tronco mesenquimais (CTM) autólogas originadas de tecido atiposo e que serão expandidas em laboratório. As CTM serão disponibilizadas para o participante da pesquisa em duas apresentações para uso em uma única sessão de tratamento: solução injetável e biocurativo estável. Conforme apresentado no controle de qualidade do produto, as CTM serão caracterizadas quanto a sua pureza, sendo necessário que apresentem ascaracterísticas determinadas pelas diretrizes da International Society for Cellular Therapy (ISCT). Todas as características de identidade, pureza, viabilidade e potência estão descritas na Tabela 1 e no item I - PROCEDIMENTOS DE AMOSTRAGEM. Quanto ao número de células (dose), serão obtidas amostras de tecido adiposo abdominal (5 a 10 gramas) que devem fornecer uma amostra inicial de aproximadamente $2x10^3$ a $3 x10^3$ células e após a expansão, pretende-se atingir pelo menos $2x10^6$ CTM para uso para o paciente (sendo as amostras de controle de qualidade e de back up já reservadas). Essas serão contadas automaticamente em *cell counter* e confirmadas através de contagem manual em camara de Neubauer. Será considerada amostra viável quando o número de células inviáveis não supere 10% do número total de células. As CTM serão aliquotadas de forma personalizada, de forma que sejam entregues $1x10^5$ CTM por 1mL de solução de plasma a ser aplicado nas bordas da úlcera em volume de 0,1mL para cada centímetro deborda. Para o curativo estável, pretende-se embeber o Fibracol com a mesma solução de plasma contendo células na proporção de $1x10^4$ CTM por cm^2 de área da úlcera a ser coberta. Assim, acredita-se que a oferta de CTM deve ser suficiente para promover o processo de reparação tecidual pretendido.

4.12 Reações ou riscos relativos ao tratamento

Riscos relativos ao uso de CTM autólogas ainda não foram descritos e para este estudo sãoconsiderados inexistentes.

Riscos relativos ao tratamento são considerados os relacionados ao procedimento anestésico cirúrgico: infecções dos sítios cirúrgicos, deiscências da ferida cirúrgica, piora da dor na área do tratamento, sangramentos, gangrena, celulites.

4.13 Seguimento, Avaliações, Procedimentos e Cronograma de Visitas

4.13.1 Avaliações Clínicas:

- Dados demográficos: inclui sexo, data de nascimento, etnia, atividade física e nível de escolaridade;
- História médica: doenças de base com data do diagnóstico;
- Exame físico: aparelho vascular periférico – inspeção (características da pele e lesões tróficas) palpação de pulsos, ausculta de sopros;
- Sinais vitais: incluem frequência cardíaca, pressão arterial e temperatura;
- Antropometria: inclui altura (somente na triagem) e peso (todas as visitas);
- Registro de medicações concomitantes: devem ser registrados todos os medicamentos, vitaminas, fitoterápicos, de uso oral, tópicos, inalatórios, intranasais e injetáveis, com posologia, dose, data de início do uso e indicação terapêutica. Em cada visita, o participante deve ser questionado se iniciouou descontinuou algum tratamento. A data de descontinuação também deve ser registrada;
- Avaliação de eventos adversos: inclui avaliação de eventos adversos locais (úlcera e pele peri úlcera)e sistêmicos (exames laboratoriais e sintoma clínicos) com data de início e término dos sintomas, intensidade, se é grave ou não, causalidade em relação tratamento do estudo (sim ou não);
- De acordo com a Conferência Internacional para Harmonização (ICH), evento adverso (grave ou não) é qualquer ocorrência clínica indesejada em participante de pesquisa clínica que recebe ou utiliza um produto ou tratamento e que não apresenta, necessariamente, relação causal com estes. Assim, um evento adverso pode ser qualquer sinal desfavorável e não intencional (inclusive um achado laboratorial anormal, sintoma ou doença temporalmente associada ao uso de um produto em investigação, considerado relacionado ou não ao mesmo). Condições preexistentes que sofrerem agravamento durante um estudo devem ser relatadas como eventos adversos;

O evento torna-se grave quando:

- Resultar em óbito
- Ameaçar a vida
- Resultar em incapacidade ou deficiência permanente, significativa ou gerar incapacidade derealizar funções normais da vida.
- Causar anormalidade congênita
- É um evento clinicamente significante conforme avaliação do pesquisador
- Avaliação - aplicação do questionário de qualidade de vida (SF-36) (Anexo I);
- Aplicação da escala de dor (Escala Visual Analógica – EVA) (Anexo II);
- Aplicação da escala de percepção de saúde (Escala EQ-5D) (Anexo III);

- Avaliação dos parâmetros laboratoriais: glicemia, hemoglobina glicada, colesterol total, HDL, LDL, triglicérides, hemograma completo, ácido úrico, uréia, creatinina, PCR, sódio e potássio. Caso haja resultado fora do padrão de normalidade, avaliar se há relevância clínica (evento adverso) ou se não há relevância clínica (não considerado evento adverso);
- Avaliação do índice tornozelo-braço (ITB) do membro acometido: o participante deve estar em decúbito dorsal horizontal e em repouso por, pelo menos, cinco minutos. Os manguitos do esfignomanômetro devem ser posicionados de forma confortável, ajustados nos braços, na mesma altura, acima do maléolo cubital com o "*cuff*" direcionado para o trajeto da artéria braquial de cada lado. Após, elege-se o braço de pressão arterial sistólica (PAS) maior para confrontá-lo com os MMII. Quando os valores de PAS dos MMSS são idênticos elege-se o braço direito (BD). Se houverdiferença nos valores, eleger o braço com maior valor de PAS. A PAS braquial será determinada nopunho no local da artéria radial com ultrassom Doppler portátil de 10 mHz. Com o mesmo equipamento a PAS do tornozelo será determinada na artéria pediosa dorsal. Para determinar o valor do ITB, divida a pressão sanguínea sistólica do tornozelo (maior valor) pela pressão sanguínea sistólica do braço (maior valor);
- Avaliação de arteriografia realizada previamente (caso o participante tenha feito este exame) descrevendo-se detalhadamente as artérias existentes e suas condições no exame arteriográfico (descrição a ser realizada por um cirurgião vascular). Será considerada válida para o estudo se realizada até 6 meses da data da triagem;
- Avaliação da ultrassonografia vascular com Doppler. Uma ultrassonografia vascular com Doppler será realizada com um equipamento de ultrassonografia Logic S7 XD Clear com transdutor linear 9MHz antes de qualquer intervenção no participante. Este deve comparecer sem nenhum preparo prévio. Ficará deitado horizontalmente sobre uma maca e um cirurgião vascular certificado realizaráum exame detalhado da circulação arterial do membro acometido, identificando a perviedade, oclusões de artérias, características das ondas de fluxo, velocidades de fluxo e estenoses;
- Avaliação da pele da perna e do pé por Termografia. Uma câmera termográfica profissional TermoCam T530-SC a uma distância de 40cm do participante, fazendo pelo menos 4 aquisições de imagem (anterior, posterior, lateral e medial). O participante deve comparecer sem nenhum preparo prévio. Ficará deitado horizontalmente sobre uma maca e um profissional da saúde treinado com o aparelho fará as fotografias;
- Avaliação da perfusão da pele com Capnografia. Um capnógrafo IntelliVue TcG10® PHILLIPS seráinstalado no membro inferior do participante, aplicando-se o sensor em um ponto determinado na base do hálux e um ponto no tornozelo atrás do maléolo medial. O participante deve comparecersem nenhum preparo prévio. Ficará deitado horizontalmente sobre uma maca e um profissional da saúde treinado com o aparelho fará as leituras de tensão de O_2 e CO_2;

4.13.2 Avaliação da úlcera:

A avaliação da úlcera será realizada pelo instrumento, escala *Pressure Ulcer Scale* for Healing, conforme abaixo:

- Avaliação de largura e comprimento (medidas em cm);
- Avaliação da quantidade de exsudato (ausente, pequena, moderada, grande);
- Avaliação quanto ao tipo de tecido (ferida fechada, tecido epitelial, tecido de granulação, esfacelo, tecido

necrótico);

Além desses, outros parâmetros serão avaliados:

- Mensuração da área (cm^2) pela análise fotográfica: a medida do tamanho da área da úlcera ocorrerá por fotografia que será obtida por câmera digital a uma distância de 30 cm da lesão identificada por etiqueta com iniciais, número do participante, número da úlcera, visita e data. A medição da área na fotografia será realizada pelo software ImageJ™;
- Caracterização das bordas (planas, pouco profundas, muito profundas, hiperceratóticas);
- Sinais de infecção (ausentes, secreção purulenta clara, secreção purulenta amarelada, secreção purulenta esverdeada) e hiperemia das bordas (ausente, pequena, moderada, grande).

4.13.3 Avaliações Laboratoriais

Os resultados das análises laboratoriais devem ser avaliados quanto à significância clínica. Serão realizadas durante o estudo as seguintes avaliações laboratoriais:

- Hemograma completo: incluirá hemoglobina, hematócrito, glóbulos vermelhos, volume corpuscular médio (VCM), hemoglobina corpuscular média (HCM), concentração de hemoglobina corpuscular (CHCM), amplitude de distribuição eritrocitária (RDW), contagem de leucócitos (WBC) com diferencial (neutrófilos, linfócitos, monócitos, eosinófilos e basófilos) e plaquetas;
- Glicemia: glicose e hemoglobina glicada;
- Perfil lipídico: serão avaliados os níveis de colesterol total, lipoproteína de baixa densidade (LDL), lipoproteína de alta densidade (HDL) e triglicerídeos;
- Função hepática: Bilirrubina total e frações, aminotransferases (TGO e TGP), fosfatase alcalina (FA), Gama glutamil transferase (Gama-GT), Albumina, Proteínas totais e frações e Coagulograma completo (TTPA e TP com INR);
- Sorologias: para HIV, Hepatites B e C e HTLV;
- Função renal: uréia, creatinina e clearance de creatinina;
- Perfil inflamatório para resposta imune: dosagem de citocinas séricas que atendam minimamente osperfis Th1, Th2 e Th17.

4.13.4 Curativos

Os participantes pertencentes ao grupo 1 que serão submetidos ao tratamento com terapia celular serão orientados a realizar a troca da cobertura secundária sempre que necessário, no mínimo de 1x/dia, mantendo uma fina camada de gaze úmida sobre a camada contendo e gaze de rayon (curativo primário). Após sete dias retornará ao centro de pesquisa, onde será retirado o curativo primário e secundário, lavado a ferida com soro fisiológico 0,9%, aplicado Hidrogel com AGE Curatec®, gaze seca e por último faixa de crepe. Será orientado a trocar o curativo no mínimo uma vez ao dia, mantendo o uso dos produtos conforme a orientação dada na consulta, durante todo o tempo do tratamento ou até que a ferida esteja cicatrizada.

Para os participantes que estiverem no Grupo 2 – controle, a ferida deve ser limpa com soro fisiológico 0,9%, aplicar a cobertura com Hidrogel contendo AGE, gaze seca e por último faixa crepe. Será orientado a trocar o curativo no mínimo uma vez ao dia, mantendo o uso dos produtos conforme a orientação dada na consulta, durante todo o tempo do tratamento ou até que a ferida esteja cicatrizada.

A partir de então, todos os pacientes seguirão a mesma escala de visitas.

4.13.5 Avaliação de Acompanhamento

Os participantes serão acompanhados e avaliados nos dias 7, 30, 60 e 90 dias após a randomização. A avaliação de encerramento será realizada na visita 6 com 120 dias. Após sua participação no estudo será encaminhado e acompanhado no ambulatório de cirurgia vascular do Hospital das Clínicas da FMB - UNESP (HC/FMB-UNESP). Neste período visitas extras podem ocorrer, caso haja necessidade (Tabela 5).

Tabela 5 Cronograma de Avaliações e Visitas

Cronograma de Atividades – ESTUDO STEM-CELL I							
**PROCEDIMEN-TOS**	**TRIA-GEM**	**VISITA1**	**VISITA 2**	**VISITA 3**	**VISITA 4**	**VISITA 5**	**VISITA 6 – Final**
	-1 até -30dias	Dia 0	7 dias da V1 (+/- 3 dias)	30 dias da V1 (+/- 3 dias)	60 dias da V1 (+/- 3 dias)	90 dias V1 (+/- 3 dias)	120 dias V1 (+/- 3 dias)
Termo de consen-ento livre e es-clarecido	X						
Consulta médica: história, examefísico	X	X	X	X	X	X	X
Sinais vitais e an-tropometria	X	X	X	X	X	X	X
Medicações con-comitantes	X	X	X	X	X	X	X
Critérios de inclu-são e exclusão	X	X					
Escala de dor (EVA)	X	X	X	X	X	X	
SF-36 e EQ-5D	X	X	X	X	X	X	
Avaliação da úlcera / imagem fotográfi-ca / curativo	X	X	X	X	X	X	
Índice Tornozelo Braço (ITB)	X			X	X	X	
Termografia	X		X	X	X	X	
Ultrassonografia scular com Dop-pler	X			X		X	
Monitorização transcutânea	X		X	X	X	X	
Coleta de sangue	X			X		X	
Sorologias: HIV, Hepatite B e C,HTLV	X						
Hemograma Com-pleto	X			X		X	
Glicose	X			X		X	
Hemoglobina Gli-cada	X			X		X	

Colesterol total, LDL, HDL, triglicérides	X			X		X	
Bilirrubina total e frações	X			X		X	
TGO	X			X		X	
TGP	X			X		X	
Fosfatase alcalina	X			X		X	
Gama-GT	X			X		X	
Proteínas Totais, albumina e globuli-na	X			X		X	
Coagulograma: TP e TTPA	X			X		X	
Citocinas imunomoduladoras: Th1, Th2 e Th17	X			X		X	
Coleta de amostra de tecido adiposo	X						
Randomização	X						
Aplicação de CTM (G1) / Aplicação de Cura-tivo(G2)		X					
Eventos adversos		X	X	X	X	X	X
Critérios de descon-tinuação		X	X	X	X	X	
OBS: os participantes do grupo controle podem fazer a triagem e visita 1 no mesmo dia desde que os resul-tados dos exames de triagem já tenham sido checados pelo investigador.							

4.13.6 Critérios de descontinuação no estudo

As descontinuações podem ocorrer pelos seguintes critérios:

- Os participantes podem se retirar do estudo, se assim o quiserem, a qualquer momento e independentemente do motivo, retirada do consentimento pelo participante do estudo;
- Por perda de seguimento;
- Evoluir com amputação maior no membro tratado;
  Indicação clínica imediata de amputação, piora clínica a critério do investigador, infecções ativas da úlcera
- Não apresentar condições clínicas necessárias para a realização do procedimento de aplicação daterapia celular no período de um mês após a data estipulada para aplicação.

Os motivos para a descontinuação do tratamento devem ser devidamente documentados pelo pesquisador. No caso de encerramento do estudo o pesquisador deve assegurar a continuidade da assistência médico/hospitalar do participante da pesquisa.

4.14 Desfechos

4.14.1 Desfecho primário

O desfecho primário a ser avaliado estará atrelado a ocorrência de eventos adversos:

Segurança:

- Maiores: amputações maiores (amputações em nível de perna ou coxa) ou também denominado taxade salvamento de membro (não ocorrência de uma amputação em relação ao grupo comparador.

Eficácia:

- O desfecho primário de eficácia deste estudo será a cicatrização total da úlcera: avaliada pela epitelização total da úlcera; curativos e imagens fotográficas e não ocorrência de amputação de membro inferior (salvamento de membro)

4.14.2 Desfecho secundário

Segurança:

- Óbito, infarto agudo do miocárdio (IAM), acidente vascular cerebral (AVC), eventos tromboembólicos como trombose venosa profunda (TVP) e embolia pulmonar (EP), quadrosinfecciosos sistêmicos e neoplasias;
- Infecções locais, sangramentos, piora da dor e aumento da área da ferida

Eficácia:

- Melhora da perfusão do membro: avaliado pela termografia (aumento significativo da temperatura do membro > 0,3ºC), ultrassonofia vascular com Doppler (perviedade, característica da onda de fluxo e velocidade de fluxo), ITB (aumento do valor do ITB >/= 0,15 para os participantes com ITB<0,9), Capnografia Transcutânea (aumento superior a 10% nos níveis de O_2 aferidos e/ou diminuição superior a 10% nos níveis de CO_2 aferidos em um ponto na base do pé – base do hálux eum ponto no tornozelo atrás do maléolo medial;
- Melhora da qualidade de vida: avaliada pela escala SF-36 (Medical Outcomes Study 36 - Item Short - Form Health Survey);
- Diminuição da dor: avaliada pela Escala Visual Analógica de Dor (EVA) e redução do uso de analgésicos (quantitativamente e qualitativamente);
- Cicatrização parcial da úlcera, avaliada pela epitelização parcial da úlcera; curativos e imagens fotográficas (obtida pela análise de fotografias com a utilização da ferramenta de medida de área do software de uso livre ImageJ™);
- Melhora do nível de percepção de saúde: avaliada pela escala EQ-5D by EuroQol Group.
- Melhora clínica: observada pela melhora dos parâmetros fisiológicos (pressão arterial) e de parâmetros laboratoriais (glicemia, hemoglobina glicada, HDL, LDL, colesterol total, triglicérides, leucograma, hematócrito, ácido úrico, uréia, creatinina, PCR, sódio, potássio, aminotransferases – TGO e TGP, bilirrubina total e frações, fosfatase alcalina – FA, Gama glutamil transferase – Gama- GT, Albumina, Proteínas totais e frações e Coagulograma completo – TTPA e TP com INR);
- Formação de tecido de granulação na úlcera a critério do pesquisador;
- Mudança no perfil inflamatório sistêmico com uma resposta imune predominantemente celular em detrimento da humoral, por meio de análises das citocinas imunomoduladoras (Th1, Th2 e Th17).

Segurança / Eficácia:

- Outros eventos adversos: qualquer ocorrência não citada nos itens anteriores e alterações negativas e clinicamente significantes de parâmetros laboratoriais (glicemia, hemoglobina glicada, HDL, LDL, colesterol total, triglicérides, leucograma, hematócrito, ácido úrico, ureia, creatinina, PCR, sódio, potássio, TGO, TGP, bilirrubinas, PTF e coagulograma) e análise histológica tecidual.

4.15 Coleta e monitoramento dos dados

4.15.1 Coleta e monitoramento dos dados do estudo clínico

A verificação e a autenticidade da qualidade dos dados inseridos na ficha clínica eletrônica, dosregistros de dados clínicos no prontuário eletrônico (MV) e dos documentos impressos ser serão realizados por procedimentos de coleta e monitoramento dos dados da equipe de pesquisa da Unidade de Pesquisa Clínica da Faculdade de Medicina de Botucatu – UNESP (UPECLIN/ORPC), conforme plano de monitorias,durante a execução e no encerramento do estudo clínico.

O Pesquisador Principal deste estudo clínico é responsável por realizar todas as etapas razoáveis quegarantam a condução adequada do protocolo do estudo clínico em relação à ética, à aderência ao protocolo eà integridade e validade dos dados registrados nos e-CRFs. Assim, a principal função da equipe de monitoramento é ajudar o Pesquisador a manter a qualidade técnica, científica, ética e regulatória.

Este estudo clínico contará com um Arquivo do Investigador (de acordo com o Documento das Américas, 2005), cuja finalidade é criar um Plano de Gerenciamento de Documentos Essenciais. Este plano servirá para listar especificamente quais documentos essenciais devem ser coletados e como será a sua manutenção durante o Ensaio Clínico.

São considerados documentos essenciais antes do início do estudo:

A) Brochura do Investigador (já realizado);
B) Protocolo e emendas assinados;
C) Informações dadas ao sujeito da pesquisa, tais como, o Termo de Consentimento Livre e Esclarecido, documentos de orientação ao participante durante o estudo;
D) Documentação financeira do estudo (orçamentos, contratos e seguros);
E) Carta de Aprovação do CEP/CONEP (Parecer Consubstanciado);
F) Carta de Aprovação da ANVISA;
G) Curriculum vitae e/ou outros documentos relevantes que evidenciem as qualificações do (s)investigador (es) e subinvestigadores;
H) Valores normais/média (s) para procedimento (s) e/ou teste (s) médicos/laboratoriais/técnicos incluídosno protocolo;
I) Certificação dos laboratórios que realizarão os procedimentos deste estudo clínico;
J) Instruções para o manuseio do (s) produto (s) sob investigação e materiais relativos ao Ensaio Clínico;
K) Registros de envio de produto (s) sob investigação e materiais relativos ao Ensaio Clínico;
L) Certificado (s) de análise de produto (s) sob investigação enviado (s);
M) Procedimentos de decodificação para a análise estatística dos dados – ensaio clínico cego para a análisede dados;

N) Lista principal de randomização;

O) Relatório de monitoramento pré-estudo;

P) Relatório de monitoramento de início do Ensaio Clínico.

Serão considerados documentos essenciais durante a execução do estudo:

A) Atualizações da Brochura do Investigador;

B) Atualizações do Protocolo;

C) Atualizações de informações dadas ao participante da pesquisa (Termo de Consentimento Livre e Esclarecido e quaisquer outras informações escritas);

D) Aprovação do CEP/CONEP para as atualizações do protocolo e das informações dadas ao participante da pesquisa;

E) Curriculum vitae de novos investigadores e subinvestigadores;

F) Atualizações de valores normais/média(s) para procedimento(s) e/ou teste(s) médicos/laboratoriais/técnicos incluídos no protocolo;

G) Atualizações de valores normais para procedimentos/testes médicos/laboratoriais/técnicos;

H) Atualização da certificação do laboratório nos procedimentos que serão realizados para o protocolo;

I) Documentação de envio de produto (s) sob investigação e materiais relativos ao Ensaio Clínico;

J) Certificado de análise para o produto de terapia celular avançada sob investigação; K) Relatórios devisitas de monitoramento;

L) Comunicações relevantes além das visitas ao local (cartas, notas de reuniões, notas de telefonemas);

M) TCLEs assinados e datados;

N) Documentos fonte;

O) Cópia das fichas clínicas (CRFs) preenchidas eletronicamente;

P) Documentações de correções nas fichas clínicas (CRFs);

Q) Notificações ao CEP sobre eventos adversos graves;

R) Notificações ao CEP de informações de relatórios de segurança do produto de terapia celular avançadasob investigação;

S) Relatórios semestrais ao CEP e anuais à ANVISA;

T) Registro de seleção dos participantes;

U) Lista de código de identificação dos participantes;

V) Registro de inclusão dos participantes;

W) Registro de que o produto sob investigação está sendo utilizado conforme descrito no protocolo;

X) Formulário de delegação de atividades e registro de assinaturas;

Y) Registro de armazenamento de amostras de CTM criopreservadas em laboratório durante o período devigência do estudo.

Serão considerados documentos essenciais para o encerramento do estudo:

A) Todos os documentos listados anteriormente;

B) Documentação de que o produto de terapia celular avançada sob investigação foi utilizado de acordocom o protocolo;

C) Documentação sobre a contagem final do produto de terapia celular avançada sob investigação recebidono local

e aplicados aos participantes, além do que não foi aplicado aos participantes;

D) Documentação da destruição das amostras retidas durante a investigação;

E) Lista completa de códigos de identificação dos participantes;

F) Certificado das auditorias que eventualmente tenham sido realizadas;

G) Relatório final de fechamento do Ensaio Clínico para submissão ao CEP/CONEP;

H) Documentação de alocação de tratamento e decodificação; I) Relatório final do Ensaio Clínico para ANVISA.

Todo o arquivo do investigador deve ser mantido arquivado por um período de 5 (cinco) anos após a

última aprovação de uma solicitação de registro no Brasil (de acordo com a norma operacional nº 1/2013 da CONEP/CNS/MS e a RDC da ANVISA 09/2015), ou por pelo menos 2 anos após a descontinuação do desenvolvimento clínico ou conclusão formal deste desenvolvimento.

Todos os dados clínicos (avaliações clínicas, avaliação da úlcera, exames laboratoriais, características do curativo e eventos adversos) além de outros dados relevantes para este estudo serão coletados em ficha clínica padronizada especificamente para este estudo de forma eletrônica (sempre com a realização de *back up*), sendo também realizado na forma física e registrada no prontuário eletrônico do participante da pesquisa da instituição (do Sistema MV - MV Informática Nordeste Ltda.).

4.15.2 Plano de Monitoria do estudo clínico

Durante a condução do estudo, a equipe de profissionais da UPECLIN/ORPC - UNESP conduzirá visitas de monitoramento periódico para garantir que o protocolo e as diretrizes da BPC sejam seguidos. Em intervalos regulares durante o estudo clínico, um representante da equipe do centro acompanhará o progresso do estudo, a aderência do participante e do investigador às exigências do protocolo. Essa equipe, vinculada pelo sigilo profissional, deve manter a confidencialidade de todas as informações médicas pessoais e identidade pessoal (de acordo com a confidencialidade e as regras de proteção de dados pessoais).O centro de estudo pode estar sujeito à revisão pelo CEP e/ou a auditorias de garantia de qualidade e/ou à inspeção pelas respectivas autoridades regulatórias. A verificação e a autenticidade da qualidade dos dados inseridos na ficha clínica eletrônica, dos registros de dados clínicos no prontuário eletrônico (MV) e dos documentos impressos serão realizados por procedimentos de coleta e monitoramento dos dados pelamesma equipe da UPECLIN/ORPC - UNESP, conforme plano de monitorias, durante a execução e no encerramento do estudo clínico.

O Plano de Monitoria deste Ensaio Clínico consiste em:

A. Atividades Treinamento (treinamento para execução dos POPs da pesquisa em todos os seus níveis, treinamentos para o correto preenchimento dos documentos necessários; além do registro de todos os treinamentos oferecidos);
B. Propiciar a maneira de comunicação do centro de pesquisa com o monitor, estabelecendo que o ponto focal de contato será o coordenador da pesquisa;
C. Estabelecer o protocolo de agendamento de visitas;
D. Determinar quais documentos devem ser revistos e coletados, e checar se a terapia celular avançadaproposta neste Ensaio Clínico está sendo corretamente processada, armazenada e distribuída;
E. Estabelecer como e quando devem ser agendadas as visitas de monitoria, quais documentos devemser revistos e coletados;
F. Verificação dos formulários de armazenamento e comprovações de qualidade da terapia celularavançada;
G. Verificação dos documentos fonte e fichas clínicas (CRFs) (Figura 1).

No fechamento do estudo, a visita de monitoria será realizada para verificar o término do estudo, a

destruição de amostras, conferência de documentos, checagem do arquivo morto dos documentos,elaboração do relatório final.

Após as devidas correções, o banco de dados será encerrado e encaminhado para análise estatística.

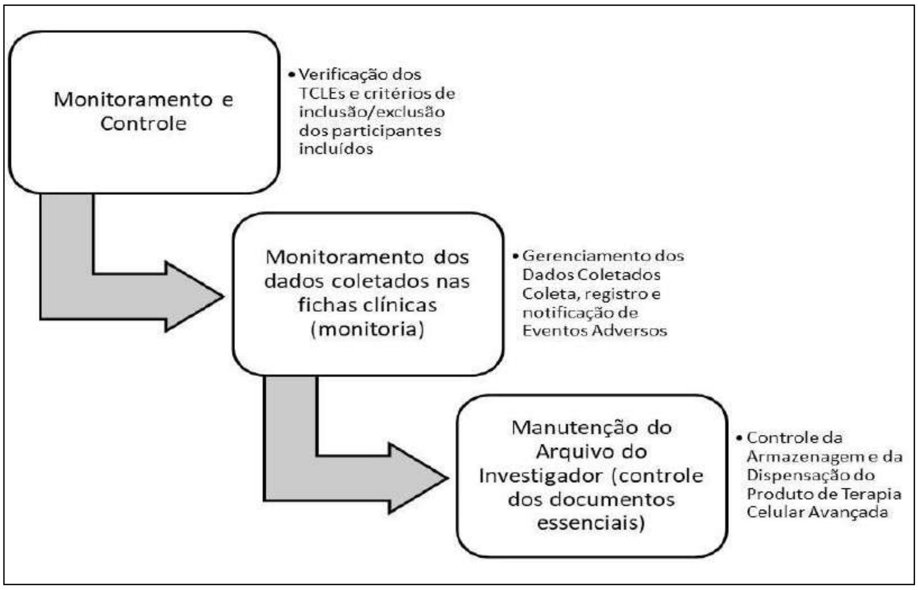

Figura 1 . Fluxo Monitoramento dos dados da pesquisa clínica com terapia celular avançadaproposta neste estudo.

4.15.3Relato e Gerenciamento de Eventos Adversos

Eventos adversos serão coletados durante todas as visitas de acompanhamento do participante do estudo clínico com terapia celular avançada (7 dias, 30 dias, 60 dias, 90 dias e 120 dias) e registrados tanto no prontuário clínico do participante como na ficha eletrônica de avaliação. Espera-se uma quantidade mínima de eventos adversos correlacionados a injeção ou aplicação tópica e na forma de biocurativo estável, não sendo bem estabelecidos, até o momento, quais as principais manifestações desses eventos, mas serão considerados eventos adversos locais (úlcera e pele peri úlcera) e sistêmicos (exames laboratoriais e sintoma clínicos) com data de início e término dos sintomas, intensidade, se é grave ou não, causalidade em relação tratamento do estudo (sim ou não). De acordo com a Conferência Internacional para Harmonização (ICH), evento adverso (grave ou não) é qualquer ocorrência clínica indesejada em participante de pesquisa clínica que recebe ou utiliza um produto ou tratamento e que não apresenta, necessariamente, relação causal com estes. Assim, um evento adverso pode ser qualquer sinal desfavorável e não intencional (inclusive um achado laboratorial anormal, sintoma ou doença temporalmente associada ao uso de um produto em investigação, considerado relacionado ou não ao mesmo). Condições preexistentes que sofrerem agravamento durante um estudo devem ser relatadas como eventos adversos. O evento torna-se grave quando: resultar em óbito; ameaçar a vida; resultar em incapacidade ou deficiência permanente, significativa ou gerar incapacidade de realizar funções normais da vida; causar anormalidade congênita; seja clinicamente significante pela avaliação do pesquisador. Medidas clínicas serão tomadas baseadas em tratamentos previamente estabelecidos para o tratamento de eventos adversos conforme eles se apresentarem.

Eventos adversos graves serão comunicados a Plataforma Brasil em até 24 horas de sua ocorrência.

4.16 Comitê Independente de Monitoramento de Segurança

Este estudo contará com um comitê independente de monitoramento de segurança, composto portrês membros da instituição sede, que avaliarão os dados de eventos adversos graves dessa pesquisa. Na confirmação de evento adverso grave, o coordenador desse ensaio clínico se compromete a declará-los na Plataforma Brasil, em até 24 horas.

O comitê será formado pelos seguintes integrantes:

- Dr. Marcone Lima Sobreira, Cirurgião Vascular;
- Dra. Paula Angeleli Bueno de Camargo, Cirurgiã Vascular;
- Dra. Mariana Thais da Silva Secondo, Cirurgiã Vascular.

Os dados de eventos adversos ou reações adversas serão coletados dos registros de saúde dainstituição de recrutamento e, quando necessário, de outros profissionais de saúde e do paciente ou representante.

As seguintes informações são apuradas: o tipo de evento ou reação, inclusive no que diz respeito a eventos pré-definidos (alergia, infecção local e sistêmica, perda do membro e óbito); data e hora de início; data e hora da administração da aplicação do biocurativo; a extensão de qualquer ligação causal ao estudo; a significância / gravidade do evento; medidas tomadas em relação ao uso posterior do biocurativo; e um resumo de forma livre.

- Eventos adversos e reações que, no julgamento do investigador principal do centro, não fazem parte do curso clínico esperado e podem estar relacionados (pelo menos possivelmente) ao estudo e são clinicamente significativos ou tiveram sequelas graves.

A alteração simples na colonização bacteriana da úlcera (cor, odor e viscosidade) são típicas dessas lesões, além de ocorrência de necrose de bordas da úlcera, perda mínima de tecidos, sangramentos sem a ocorrência de choque hipovolêmico e sem alterações significativas da hemoglobina sérica (queda menor que1g/mL) não serão relatadas como eventos adversos relacionados aos procedimentos do estudo.

Período de vigilância para reações e eventos adversos, e permitindo uma margem de segurança, chamando a atenção dos pesquisadores, deve incluir o período desde a inscrição até 4 meses após aadministração do biocurativo, exceto por reações alérgicas que serão limitadas a 7 dias.

4.17 Processo de relatório de segurança

Os eventos e reações adversas relatáveis serão comunicados pelos investigadores do centro ao investigador principal. Em geral, isso ocorrerá por escrito dentro de 24h após o investigador do centro tomarconhecimento do evento. O comitê de gestão da UPECLIN avaliará todos os relatórios de segurança recebidos dos investigadores. O investigador chefe incluirá esses dados na Plataforma Brasil e comunicaráàs autoridades regulatórias atualizações de segurança de acordo com os relatórios.

4.18 Plano de análise interinas

Este estudo conta com plano de análise interina, que será realizada por um estatístico independente

quando o estudo já tiver incluído metade dos participantes, neste momento serão considerados indícios de benefícios ou maleficio do tratamento e caso encontre-se evidência de prejuízo aos participantes o estudo será interrompido por razões éticas.

4.19 Medicações concomitantes

Não haverá nenhum tipo restrição ao uso de medicamento concomitantes, exceto medicamentos tópicos para úlceras que interfiram na pesquisa.

4.20 Critérios de descontinuidade dos participantes

As descontinuações podem ocorrer pelos seguintes critérios:

- Os participantes podem se retirar do estudo, se assim o quiserem, a qualquer momento e independentemente do motivo, retirada do consentimento pelo participante do estudo;
- Por perda de seguimento;
- Ocorrência de qualquer tipo de eventos adversos não esperados.

Os motivos para a descontinuação do tratamento devem ser devidamente documentados pelo pesquisador. No caso de encerramento do estudo o pesquisador deve assegurar a continuidade da assistênciamédico/hospitalar do participante da pesquisa.

4.21 Métodos estatísticos

Os resultados obtidos através dos desfechos primários e secundários serão tabulados semanalmente em planilha eletrônica pela equipe de seguimento do estudo. É estimado uma amostra de conveniência de 40participantes. Os dados serão inicialmente avaliados utilizando: 1- estatística descritiva, com análises de mediana/variância e/ou média/desvio padrão para variáveis quantitativas e; 2- análises de tabela de contingência para as variáveis categóricas/qualitativas. Todos os dados monitorados no estudo para os grupos G1 (Aplicação de CTM) e G2 (Aplicação de Curativo) serão comparados estatisticamente. Os dados quantitativos serão avaliados quanto sua distribuição (utilizando o teste de normalidade de Shapiro-Wilk) e comparados utilizando o teste T ou o teste de Wilcoxon, dependendo do tipo de distribuição das variáveis. Para as variáveis categóricas, as diferenças serão avaliadas através do teste do Qui-quadrado de Pearson ou teste exato de Fisher. Coeficiente de Bonferroni e análises multivariadas serão também utilizadas. Em todos os testes estatísticos, o nível de significância da variável independente será estabelecido em 5% ($p < 0{,}05$). Por final, é proposta a avaliação de todo o conjunto de dados por análise de correspondência múltipla (popularmente conhecida como MCA, sigla do termo em inglês *Multiple Correspondence Analysis*). A MCAé uma técnica exploratória e sua aplicação resulta em representações gráficas desse conjunto de dados emum mesmo plano fatorial, possibilitando a inferência de correspondência entre as informações a partir de distâncias euclidianas entre esses dados no plano, formando agrupamentos (ou *clusters*) de informações correspondentes. Para sua aplicação, cada variável quantitativa será categorizada de acordo com os quartisde distribuição dos seus respectivos dados.

Dados ausentes são comuns em estudos clínicos longitudinais e podem estar relacionados a: perda do participante; doenças não relacionadas ao tratamento em estudo; erros de transcrição ou digitação;

ineficiência do tratamento proposto; falta de cooperação do participante; questões mal formuladas em um questionário. Para lidarmos com esse problema, prevemos a realização de estratégias estatísticas bem estabelecidas como: análise dos casos completos; análise dos casos disponíveis e; procedimentos estatísticosbaseados em imputação. A escolha do método mais apropriado será realizada posteriormente à coleta dos dados e de forma personalizada à cada tipo diferente de possível variável/dado ausente.

5. RESPONSABILIDADES

5.1 Boas Práticas Clínicas

Este estudo clínico será conduzido em conformidade com todas as leis nacionais e diretrizesinternacionais aplicáveis; de acordo com os princípios éticos definidos na 18th World Medical Assembly, Helsinque, 1964, e com todas as emendas aplicáveis, e com as diretrizes da ICH para as Boas Práticas Clínicas (BPC).

5.2 Princípios Éticos

Este estudo foi desenhado, planejado e será conduzido de acordo com os princípios éticos para pesquisa clínica definidos na Declaração de Helsinque, nas orientações das Boas Práticas Clínicas (BPC) e exigências regulatórias locais para pesquisa clínica. O estudo iniciará assim que for obtida a aprovação por escrito do Comitê de Ética em Pesquisa (CEP) e os participantes serão incluídos somente quando o processodo consentimento livre e esclarecido tenha sido realizado e o termo de consentimento livre e esclarecido (TCLE) tenha sido assinado.

5.2.1 Leis e Regulamentações

Este estudo clínico será conduzido em conformidade com todas as diretrizes internacionais e leis nacionais aplicáveis, bem como com todas as diretrizes aplicáveis.

5.2.2 Consentimento Livre e Esclarecido

O investigador, ou a pessoa designada pelo Investigador e sob sua responsabilidade, deve informar, esclarecer e orientar o participante sobre todos os aspectos pertinentes ao estudo clínico, incluindo as informações por escrito como a aprovação pelo CEP. Os participantes devem receber as informações disponíveis sobre o estudo, na linguagem e nos termos para que possam compreender.

Antes que qualquer procedimento do estudo clínico seja realizado no participante, os TCLEs devem ser assinadas, o nome preenchido e datado pelos participantes ou por seu representante legal e pela pessoa que conduziu a discussão do consentimento livre e esclarecido. Uma cópia do TCLE assinado e datado será fornecida ao participante.

5.2.3 Comitê de Ética em Pesquisa (CEP)/CONEP

Este protocolo de estudo clínico será submetido ao CEP pelo investigador e após sua aprovação poderá iniciar os procedimentos do estudo.

5.2.4 Emenda ao protocolo de estudo clínico

Durante a execução do estudo clínico, qualquer desvio, emenda ou modificação ao protocolo será submetida ao CEP antes da implantação, salvo se a alteração for necessária para eliminar um risco imediato aos participantes, caso em que o CEP deve será informado o quanto antes; assim como qualquer evento que possa afetar a segurança dos participantes ou quando a(s) alteração(ões) envolverem somente aspectos logísticos e administrativos do estudo. As atualizações da Brochura do Investigador serão enviadas ao CEP.

Um relatório de progresso será enviado ao CEP, semestralmente, e um resumo do desfecho do estudo clínico no final do estudo clínico.

Caso a emenda exija alteração no TCLE, o investigador deve receber a aprovação do CEP em relação ao TCLE revisado, antes da sua implantação. A assinatura do participante deve ser obtida novamente,se necessário.

5.2.5 Desvios ao Protocolo

Neste estudo, um desvio ao protocolo é considerado qualquer falta de cumprimento dos procedimentos ou requisitos definidos na versão do protocolo aprovada pelo Comitê de Ética e ANVISA.

Um desvio é considerado menor se não tiver implicações relevantes na integridade do estudo, na qualidade dos dados ou nos direitos e segurança dos participantes da pesquisa.

Um desvio é considerado maior se compromete a qualidade dos dados ou a integridade do estudo, ou que possa afetar a segurança ou os direitos dos participantes da pesquisa. Desvios maiores ao protocolo podem incluir, entre outros: erros na alocação do produto de investigação, a utilização de tratamentos não permitidos pelo protocolo, baixa adesão ao tratamento, dados não coletados ou perda de seguimento.

Todo e qualquer desvio (maior ou menor) e a razão para o mesmo devem ser documentados no prontuário médico.

5.3 Definição dos Dados-fonte

Documentos-fonte são definidos como documentos, dados e registros originais. Isso inclui, dentre outros: registros hospitalares, históricos clínicos, planilhas de documentos-fonte específicos do estudo, laudos e requisições laboratoriais, exames por imagem, ECGs, registros de dispensação de medicação, impressões de computador, fontes de informações/dados eletrônicos e qualquer outra documentação relacionada ao participante. O investigador pode nomear outros médicos, conforme considerar apropriado, como subinvestigadores para auxiliar na condução do estudo clínico de acordo com o protocolo. Os subinvestigadores serão supervisionados pelo e trabalharão sob a responsabilidade do investigador. Oinvestigador fornecerá aos subinvestigadores uma cópia do protocolo do estudo clínico e todas as informações necessárias. Os dados coletados neste estudo serão utilizados somente para a(s) finalidade(s) doestudo.

5.3.1 Retenção de registro no centro de estudo – Arquivo de documentos

O pesquisador e sua equipe manterá confidencial toda a documentação do estudo e tomarão medidaspara evitar a destruição precoce ou acidental dos documentos.

Os documentos do estudo serão arquivados no centro de pesquisa por 5 anos após a conclusão ou descontinuação deste estudo clínico para permitir que os dados sejam averiguados, monitorados e ou inspecionados.

O pesquisador disponibilizará todos os documentos-fonte e outros registros do estudo aos membros do CEP e ou autoridades regulatórias nacionais, se necessário.

5.4 Compensação do Seguro

O Pesquisador atesta que providenciará uma apólice de seguro de responsabilidade civil para este estudo. Esta apólice de seguro está em conformidade com as leis e exigências locais.

5.5 Publicações e Comunicações

O pesquisador compromete-se a publicar e divulgar seus resultados. Este estudo representa um esforço conjunto do pesquisador, dos investigadores, da equipe do estudo, Instituição e M.S. e, como tal, estes concordam que a revisão deve ser realizada por todas as partes no preparo de documentos científicos para publicação ou apresentação. Os principais achados do estudo serão submetidos à publicação em revista indexada. O pesquisador se reserva o direito de escolher o primeiro e último autor da publicação. Os demais membros da equipe do estudo serão nomeados coautores do artigo principal, considerando sua participação efetiva no desenvolvimento e execução do estudo, respeitando ainda o número de autores máximo estabelecido pela revista onde será publicado. Os membros da equipe do estudo podem apresentar propostas de publicações e apresentações paralelas dos resultados deste estudo clínico. No entanto, todas as publicações e apresentações propostas pelos investigadores/membros da equipe serão analisadas pelo pesquisador.

5.6 Controle de Qualidade e Garantia de Qualidade

Durante a condução do estudo, a UPECLIN conduzirá visitas de monitoramento periódico para garantir que o protocolo e as diretrizes da BPC sejam seguidos. Em intervalos regulares durante o estudo clínico, um representante da equipe do centro acompanhará o progresso do estudo, a aderência do participante e do investigador às exigências do protocolo. Estas visitas de monitoramento incluirão, dentre outros, a revisão dos seguintes aspectos: TCLE, recrutamento e acompanhamento do participante, relato e documentação de EAG, documentação de EA, aderência do participante ao tratamento, uso de medicamento concomitante e qualidade dos dados.

De acordo com as diretrizes da ICH para as Boas Práticas Clínicas, a equipe de monitoramento deveverificar a exatidão das entradas no e-CRF em relação aos documentos-fonte. O TCLE incluirá uma declaração de que o participante permite que a equipe devidamente autorizada do pesquisador, o Comitê de Ética em Pesquisa (CEP) e as autoridades regulatórias tenham acesso direto aos registros médicos originais que corroboram os dados dos e-CRFs (por exemplo, arquivo médico do participante, diários, registros laboratoriais originais, etc.). Essa equipe, vinculada pelo sigilo profissional, deve manter a confidencialidadede todas as informações médicas pessoais e identidade pessoal (de acordo com a confidencialidade e as regras de proteção de dados pessoais).

O centro de estudo pode estar sujeito à revisão pelo CEP e/ou a auditorias de garantia de qualidade e/ou à inspeção pelas respectivas autoridades regulatórias.

O Pesquisador deste estudo clínico é responsável por realizar todas as etapas razoáveis que garantam a condução adequada do protocolo do estudo clínico em relação à ética, à aderência ao protocolo e à integridade e validade dos dados registrados nos e-CRFs. Assim, a principal função da equipe de monitoramento é ajudar o Pesquisador a manter a qualidade técnica, científica, ética e regulatória.

6. RESULTADOS ESPERADOS E SUA RELEVÂNCIA PARA OS DOENTES E O SUS

6.1 Resultados esperados

Os resultados e as contribuições têm dois aspectos a serem considerados:

Dentre os resultados esperados, estão:

- Efetivação do Centro de Processamento Celular do HC da FMB – UNESP. Espera-se que a possibilidadede efetivação do CPC do HC UNESP traga um enorme impacto científico e tecnológico nas abordagens terapêuticas com células-tronco, além de permitir que o CPC possa atingir nível de produção em escala das células-tronco expandidas *in vitro*. O prazo de implantação e de execução deste primeiro projeto servirão de base para capitanear novos estudos com terapia celular avançada. Para este objetivo, apenas a execução deste projeto já fornece a mensuração dessa meta.
- Divulgação da tecnologia de terapia celular em nível de ensino médio, ensino superior, pós-graduação e entre profissionais da saúde e afins. O impacto da divulgação da informação deve se refletir tanto na formação de profissionais com senso crítico e adequados ao tempo da evolução tecnológica que a ciênciaproporciona. A forma de mensuração será obtida pela avaliação do cumprimento das metas de extensão e das metas de divulgação sendo cumpridas conforme cronograma anteriormente apresentado.
- Para os participantes do estudo, o projeto visa disponibilizar uma terapia inovadora e de vanguarda cien- tífica, com a oferta de uma alternativa que possa melhorar as chances de não amputação do membro acometido, além de proporcionar uma possível melhora na qualidade de vida e na saúde no geral. Os resultados deste estudo quanto ao salvamento dos membros afetados, a taxa de cicatrização das feridas, os questionários de qualidade de vida, exames de sangue e outros exames embasarão os resultados efetivos para os participantes quanto a efetividade do tratamento proposto (terapia celular avançada) dentro dos prazos previstos para este estudo.
- Para o SUS e as políticas públicas de saúde, a terapia celular avançada urge de comprovação científica através de estudos realizados em território nacional, para o embasamento da quebra de alguns paradig- mas que em países da Europa, Ásia e EUA já se consolidaram como alternativa. Isso não pode ser men-surável por números simples de um único estudo, mas, se ao final deste estudo e desta linha de financia- mento proposto pelo CNPq em parceria com o DECIT, for possível observar a proposição de novos pro- jetos de pesquisa com terapia celular avançada, já representará um avanço nesse sentido de popularizaçãoda tecnologia em solo brasileiro.

- Para a precificação da tecnologia, a realização deste estudo pode contribuir com o fornecimento de valo- res médios necessários para um tratamento dessa natureza e o estabelecimento de regras para investimen-tos dessa natureza.
- Para os pesquisadores envolvidos a participação em congressos científicos, publicação de artigos e o contato com pesquisadores de outras instituições nacionais e internacionais proporcionarão o crescimentodo grupo científico, estabelecimento do grupo científico, promoção da pesquisa brasileira de ponta e in-ternacionalização.
- Propiciará o treinamento técnico avançado dos membros dessa pesquisa, que serão mensuráveis pelas bolsas DT que serão fornecidas e o incremento curricular e prático dos técnicos desta pesquisa.
- Para os familiares e cuidadores desses pacientes, o tratamento dos participantes poderá representar um alívio do sofrimento em ver paciente sofrendo sem nenhuma alternativa de tratamento. Isso não poderá ser mensurado neste estudo, mas refletirá em divulgação da terapia celular avançada como alternativa terapêutica real na sociedade, aumentará a visibilidade e trará esperança associada a visão de uma Universidade e do SUS empenhados em promover a melhoria da saúde da população.
- Possível obtenção de patente e licença de produto de terapia celular avançada na ANVISA para futura comercialização. Essa meta pode ser atingida a depender dos resultados que forem encontrados no decorrer do estudo. A mensuração se faz através do número de processos de depósito de patente e do número de protocolo de registro e licença de produto de terapia celular avançada na ANVISA.
- Espera-se, por fim, que um protocolo de tratamento com terapia celular avançada para DAP em ICM com risco iminente de perda de membro seja estabelecido ao final do projeto. Esse dado também será mensurável através das publicações científicas relacionadas a este estudo.

6.2 Alinhamento com políticas públicas de saúde

Este projeto encontra-se alinhado com a política pública de saúde em doenças crônicas não transmissíveis (DCNT), no sentido de promover ações de contenção dos potenciais fatores de risco para doença arterial periférica e demais doenças cardiocirculatórias (instrução aos participantes, familiares,cuidadores, pacientes do ambulatório de doenças arteriais periféricas do HC da FMB UNESP, promovendo então a redução dos índices de mortalidade, diminuição nos índices de internações devido a complicações dadoença arterial periférica e das úlceras arteriais, promovendo tratamento e internação efetiva para o tratamento DCNT (no caso, uso da terapia celular avançada para pacientes com úlceras arteriais e alto risco de perda de membro). Ainda alinha-se com a premissa de tratar pacientes de alto custo e complexidade com uma alternativa de alta tecnologia, promovendo ações que serão executadas imediatamente para que forneçam resultados eficazes em termos de qualidade de vida. Dessa forma, pretende-se evitar o agravamento das condições de saúde do indivíduo em portadores de doença arterial periférica e o plano do estudo está articulado com ações que promovem a melhoria do sistema de informações, capacitação de profissionais de saúde, financiamento adequado, obtenção de medicamentos e tecnologia essencial.

6.3 Impactos para o sus

Trata-se de uma alternativa como solução de tratamento clínico para pacientes em estágio avançado da DAP, onde apresentam isquemia crítica de membros e úlcera arterial sem possibilidade de revascularização, o que aumenta muito a probabilidade de amputação de membro. Pouquíssimos estudos clínicos com terapia celular avançada têm sido realizado no Brasil e somente recentemente tivemos um marco regulador mais claro a esse respeito, com a divulgação de RDCs que permitem um manejo adequado dessa tecnologia. Este estudo pode ajudar a fortalecer a terapia celular no país, sendo provavelmente mais uma alternativa que poderá ser disponibilizada pelo SUS e que depende de comprovação científica. Os pacientes que participarão deste estudo, geram, naturalmente, um grande custo para o SUS, uma vez que sãoportadores de uma doença grave, associada a outras comorbidades que elvam ao consumo contínuo de analgésicos, sequencias prolongadas de tratamentos com antibióticos muito caros, custos com curativos, custos com transporte, internações seriadas e por fim culminam com a amputação do membro, tornando-se em sua maioria dependentes do sistema previdenciário brasileiro, perdendo anos de produtividade laboral, comprometendo famílias com cuidados demasiados, além de causar frustração e problemas psicológicos graves nesses pacientes, mais uma vez, onerando o SUS.

No sentido terapêutico, a terapia celular avançada pode proporcionar uma evolução em direção a fronteira do conhecimento e das possibilidades de terapêuticas oferecidas pelo SUS, reforçando seu valor frente a população usuária.

Dessa forma, apesar da parca literatura científica disponível a esse respeito e da inexistência de um estudo clínico com semelhança ao estudo proposto neste protocolo, existe embasamento literário científico suficiente que garante que este estudo não se trata de um experimentalismo desproposital.

7. Custo-Efetividade

A elaboração desses cálculos de custo benefício (custo/efetividade) será feita por meio de modelos de decisão clínica e avaliação econômica pela elaboração de uma árvore de decisão e se possível a realização de modelos de Markov. Serão levadas em consideração:

a. Identificação de Custos - serão listados todos os custos associados ao novo procedimento, incluindoequipamentos médicos, treinamento da equipe, instalações, materiais consumíveis e custos operacionais.
b. Benefícios Clínicos - serão descritos os benefícios clínicos esperados do procedimento, como melhoria na saúde do paciente, redução de complicações e melhor qualidade de vida
c. Análise de Custos Diretos - serão calculados os custos diretos, como compra de equipamentos, manutenção, treinamento de pessoal e custos de procedimento em si.
d. Análise de Custos Indiretos - serão considerados todos os custos indiretos, como espaço físico,energia, seguro e custos administrativos.

e. Benefícios Econômicos - serão avaliados os benefícios econômicos, como redução de internações hospitalares, diminuição de visitas ao médico ou ganhos econômicos devido à recuperação de produtividade do paciente.
f. Análise de custo-efetividade - será calculada a relação entre os custos totais e os benefícios clínicos e econômicos esperados. Isso pode ser expresso em termos de custo por unidade de resultado, comocusto por vida salva, por ano de vida ganho ou por qualidade de vida aprimorada a depender dos da-dos que forem encontrados. Lembrando que os pacientes serão acompanhados inicialmente por 4 meses, mas para esses dados serão consultados por um ano.
g. Comparação com alternativas - o novo procedimento será comparado com a terapia existente, paradeterminar se é mais custo-efetivo.
h. Avaliação de Riscos - serão avaliados os riscos potenciais, como incertezas nos benefícios ou custos inesperados, agindo para diminuí-los.
i. Comunicação e tomada de decisão - as descobertas serão informadas de forma clara e concisa para partes interessadas, como médicos, gestores de saúde e tomadores de decisão para que possam apoiar a adoção do novo procedimento.
j. Monitoramento e avaliação contínua - monitoramento para acompanhar os custos e benefícios aolongo do tempo

8. Impactos e contribuições para o complexo econômico-industrial da saúde

A introdução da terapia celular avançada pode trazer vários benefícios ao sistema de saúde nacional:

- Melhoria da Qualidade de Atendimento – o novo procedimento pode melhorar a qualidade do atendimento médico, oferecendo um tratamento mais eficaz e menos invasivos para pacientes em risco de amputação de membros inferiores;
- Redução de Custos – a nova tecnologia de terapia celular pode reduzir os custos a longo prazo, especialmente se for comprovadamente mais eficaz na prevenção de complicações ou na redução dotempo de internação;
- Avanços Tecnológicos - A pesquisa e o desenvolvimento de novos procedimentos em terapia celularpodem impulsionar a indústria médica e tecnológica no país, criando empregos e oportunidades de negócios, além de ser mais uma opção que pode ser oferecida para os pacientes atendidos pelo SUS;
- Reconhecimento Internacional – os procedimentos inovadores em terapia celular podem atrair a atenção internacional, gerando prestígio para a comunidade médica e científica do Brasil;
- Atração de Investimentos – inovações em saúde podem atrair investimentos de instituições de pesquisa, empresas farmacêuticas e investidores estrangeiros, impulsionando a economia em nível local, regional e nacional. Possivelmente teremos o surgimento de pelo menos uma startup local nessa área de desenvolvimento tecnológico em saúde;
- Fortalecimento da Pesquisa - o desenvolvimento de novos procedimentos em terapia celular fortalece a colaboração entre instituições acadêmicas e médicas nacionais e internacionais, fortalecendo a pesquisa em saúde no país;

- Redução da Dependência de Tecnologias Estrangeiras - ao se desenvolver procedimentos próprios em terapia celular, o Brasil pode reduzir sua dependência de tecnologias e produtos importados, economizando divisas para o país;
- Acesso Universal - se o procedimento for eficaz e acessível, pode contribuir para a promoção do acesso universal à saúde no país.

Em resumo, a inovação em procedimentos médicos no Brasil pode trazer uma série de benefícios para o sistema de saúde, desde que seja acompanhada por uma avaliação rigorosa e uma estratégia adequada de implementação.

9. CARÁTER INOVADOR DO PROJETO

O projeto de pesquisa clínica com terapia celular avançada proposto no presente estudo pretende realizar o tratamento de pacientes com de doença arterial periférica (DAP) com isquemia crítica de membro (ICM), com úlcera arterial em membro inferior por tempo maior que 3 semanas, sem possibilidade de revascularização do membro, revascularização incompleta ou revascularização com reestenose ou oclusão (Fontaine IV e Rutherford 5).

O produto deste estudo consiste em uma terapia celular avançada com protocolo inovador que foi desenvolvido por esta equipe de estudo, o qual utilizará células-tronco mesenquimais (CTM) adultas autólogas obtidas de tecido adiposo abdominal e diluídas em plasma autólogo em associação com a aplicação das mesmas CTM na forma de um biocurativo estável, em uma única sessão de tratamento. Tanto as CTM que serão injetadas de forma perilesional como o biocurativo estável contendo as mesmas células devem fornecer um grande suplemento de CTM tanto nas bordas quanto na superfície da lesão, o que deve desempenhar um papel modulador no processo inflamatório e reparatório da lesão.

9.1 Estudo de viabilidade patentária

A análise prospectiva do processo patentário do processo envolvido neste projeto, incluindo o biocurativo estável utilizando plasma autólogo e a utilização de membrana de colágeno Fibracol para viabilização de CTM de forma tópica sobre a úlcera arterial, em detrimento ao número de artigos científicos encontrados, o que mostra alta maturidade da rota tecnológica. O estudo da análise prospectiva da patente mostrou que a tecnologia utilizada, não obstante seu baixo grau de maturidade tecnológica, possui caráter inovador perante o Instituto Nacional de Propriedade Intelectual, INPI.

Adicionalmente, foi realizada uma busca em bases de dados de patentes e foram identificados os seguintes documentos:

[1]https://patents.google.com/patent/KR20090086066A/en?q=stem+cell+AND+wound+heal&oq=stem+cell+AND+wound+heal

[2]https://patents.google.com/patent/US20030021850A1/en?q=stem+cell+AND+wound+heal&oq=stem+cell+AND+wound+heal

Dessa forma, os documentos identificados antecedem a tecnologia e com certa similaridade com atecnologia proposta neste estudo, porém a tecnologia proposta avança na parte técnica de manejo da terapiacelular em pacientes com DAP e isquemia crítica por incrementar os meios de aplicação das células, tantopor injeções quanto através de uso tópico com curativo estável, sendo uma tecnologia inovadora e inventiva.Considerando os benefícios da tecnologia apresentada, recomenda-se avançar no desenvolvimento da tecnologia para se atingir escalas maiores de maturidade para ter apelo comercial em aplicações,
principalmente, no setor de terapia celular avançada.

10. PLANEJAMENTO DA GESTÃO DE RISCOS:

Quadro 1 – Gestão de riscos relativos a execução do presente estudo.

Evento de Risco	Natureza do Risco	Causa	Consequência	Identificação de Nível	Prevenção /Monitora-mento	Procedimentos de minimização
Não atingir o número de participantes previstos no estudo	Operacional e Financeiro	Agravamento decorrente da condição clínica do doente, amputação e ocorrência de morte	Não cumprimento dos prazos previstos no cronograma	Baixo	Reavaliação dos processos empregados no recrutamento	Atuar para promover um melhor controle clínico dos participantes
Contaminação da cultura de células deum parti- cipante ouContaminação do LPC	Técnico e Financeiro	Presença de microrganismos nas culturas celulares<br>Presença de microrganismos em vários seguimentos de cultura e produtos utilizados	Atraso no início do tratamento e novo procedimento de expansão celular<br>Atraso no início do tratamento e descontaminação do LPC	Baixo	Seguir adequadamente o manual de Boas Práticas Laboratoriais para cultura celular e atenção aos protocolos operacionais padrão	Revisão de protocolos operacionais padrão e de todas as fases de manipulação de tecidos e culturas celulares

Vale ressaltar que a prevenção de eventos relacionados a perda de cultura por contaminação, apesar de promover algum aumento nos custos, já está previsto e minimizado nos protocolos que serão empregadosneste ensaio clínico. Quanto aos possíveis atrasos de inclusão ou tratamento dos participantes, pode-se afirmar que dificilmente gerarão atrasos de cronograma geral, uma vez que o protocolo já prevê uma razoável margem temporal para a conclusão deste estudo. Quanto ao estabelecimento do número de participantes que concluirão o estudo, acredita-se que o impacto do tratamento seja visualizado até mesmo com 50% da amostra proposta, mas se esse número mínimo de participantes (n=20) não for atingido, novos participantes serão incluídos até que este número mínimo seja atingido para que não haja comprometimento das análises de resultados.

11. PROPRIEDADE INTELECTUAL

A propriedade intelectual é do pesquisador coordenador do estudo.

Este estudo tem potencial para gerar uma patente (curativo) que deve ser depositada em nome do pesquisador e da instituição sede do estudo (UNESP).

O custo referente ao depósito inicial da patente será da instituição sede (UNESP/FMB).

Para o licenciamento e transferência da tecnologia gerada por esse estudo e possivelmentepatenteada, pretendemos realizar uma parceria público-privada.

A política para eventuais ganhos econômicos auferidos com a exploração da invenção gerada peloestudo será compartilhada entre o pesquisador, a instituição (UNESP/FMB) e o Ministério da Saúde (MS).

O pesquisador não deve estar sob obrigação de patentear, desenvolver, comercializar ou utilizar deoutra forma os resultados do estudo clínico.

Caso seja necessário, o investigador e/ou os subinvestigadores e equipes do estudo e do centro de pesquisa devem fornecer toda assistência solicitada pela Instituição e Ministério da Saúde, às custas destes, para obtenção e defesa de qualquer patente, incluindo a assinatura de documentos legais.

12. PLANO DE TRABALHO

12.1 Identificação do objeto a ser executado - Plano de trabalho e cronograma:

Tabela 6. Cronograma

META	ATIVIDADES	DATA
Início da execução do ensaio clínico	Os pacientes que são atendidos no ambulatório de cirurgia vascular do HC/FMB-UNESP/UNESP e que atendam os critérios de elegibilidade serão convidados a participar da pesquisa que será executada na Unidade de Pesquisa Clínica da Faculdade de Medicina de Botucatu (UPECLIN – FMB/UNESP).	01/06/2025
Fim do período de inclusãode participantes no estudo	Nesta etapa espera-se que o tamanho amostral proposto tenha sido atingido, pois novos participantes não poderão mais ser incluídos. Os pacientes anteriormente incluídos continuarãosendo atendidos conforme cronograma do estudo	01/07/2027
Encerramento do estudo clínico	O encerramento do estudo clínico ocorrerá quando o último participante incluído realiza a última visita do estudo, conforme cronograma. Após a participação no estudo, os participantes serão orientados e acompanhados no ambulatório de cirurgia vascular do HC/FMB-UNESP/UNESP, com a mesma equipe médica que conduziu o estudo.	20/12/2027

Elaboração e envio de relatórios finais	Os relatórios serão elaborados e enviados conforme normas dos órgãos regulatórios competentes.	20/12/2027

12.2 Elaboração, preparação e envio de dossiês regulatórios

Elaborar protocolo do ensaio clínico, a brochura do pesquisador, o dossiê de Desenvolvimento Clínico de Produto de Terapia Avançada Institucional (DDCTA) (nova tecnologia com CTM), termo de consentimento livre e esclarecido, declarações institucionais; registrar protocolo de ensaio clínico em plataforma virtual de acesso livre (Clinical Trials; REBEC); submissões éticas e regulatórias, elaborar POP, formulário de Relato de caso (FRC), termos de acordo/contratos, preparar dossiês conforme normas da ANVISA e CEP/CONEP e enviar.

12.3 Início da execução do estudo clínico

Os pacientes que são atendidos no ambulatório de cirurgia vascular do HC/FMB-UNESP/UNESP e que atendam os critérios de elegibilidade serão convidados a participar da pesquisa que será executada na Unidade de Pesquisa Clínica da Faculdade de Medicina de Botucatu (UPECLIN – FMB/UNESP).

12.4. Execução do estudo clínico

Continuidade na inclusão e atendimento dos participantes já randomizados, realização de monitorias,conforme Manual de Boas Práticas Clínicas (GCP/ICH), para garantir a qualidade dos dados coletados, preenchimento do banco de dados, preparo e envio de relatórios semestrais ao CEP, atualização de documentação na ANVISA (se necessário), entre outros.

12.4 Fim do período de inclusão de participantes no estudo

Nesta etapa espera-se que o tamanho amostral proposto tenha sido atingido, pois novos participantes não poderão mais ser incluídos. Os pacientes anteriormente incluídos continuarão sendo atendidos conforme cronograma do estudo.

12.5 Encerramento da execução do estudo clínico

O encerramento do estudo clínico ocorrerá quando o último participante incluído realiza a última visita do estudo, conforme cronograma. Após a participação no estudo, os participantes serão orientados e acompanhados no ambulatório de cirurgia vascular do HC/FMB-UNESP/UNESP, com a mesma equipe médica que conduziu o estudo.

12.6 Gerenciamento dos dados e estatística

A verificação e a autenticidade da qualidade dos dados inseridos na ficha clínica serão realizados pela UPECLIN/ORPC, conforme plano de monitorias, durante a execução e no encerramento do estudo clínico. Após as devidas correções, o banco de dados será encerrado e encaminhado para análise estatística.

12.7 Elaboração e envio de relatórios

Os relatórios serão elaborados e enviados conforme normas dos órgãos regulatórios competentes (ANVISA, CEP/CONEP). As prestações de conta e apresentação dos resultados serão feitas conforme diretrizes dos financiadores do projeto.

12.8 Participação em eventos científicos divulgação dos resultados

O coordenador do projeto e membros da equipe participarão durante este período de reuniões com órgãos governamentais com o objetivo de apresentar a nova tecnologia/ terapêutica e sua possibilidade de inserção no Sistema Único de Saúde. Acreditamos que o estudo permitirá que seja disponibilizado mais umaalternativa de tratamento para estes doentes/pacientes, contribuindo para diminuição de óbitos, cirurgias de amputações de membros inferiores, tempo de internação e melhora na qualidade e expectativa de vida de pessoas com doença arterial periférica.

Seminários de acompanhamento e avaliação do projeto, apresentação em reuniões científicas como congressos nacionais e internacionais, elaboração de artigos para publicação em revistas científicas conceituadas deve ocorrem durante e ao término do projeto.

13. RESUMO DOS INVESTIMENTOS

CUSTEIO	CAPITAL	BOLSAS	TOTAL
R$ 2.566.20,00(58%)	R$1.159.000.00 (26%)	R$ 6888000,00 (16%)	R$ 4.414.000,00 (100%)

14. CONTRAPARTIDA DA INSTITUIÇÃO

A contrapartida para a condução deste estudo compreende os recursos humanos e infraestrutura institucional (HC-FMB-UPECLIN/UNESP).

15. REFERÊNCIAS

1. Mayerl C, Lukasser M, Sedivy R, et al. Atherosclerosis research from past to present—on the track of two pathologists with opposing views, Carl von Rokitansky and Rudolf Virchow. Virchows Archiv 2006;449:96-103.
2. Yusuf S, Reddy S, Ôunpuu S, et al. Global burden of cardiovascular diseases: part I: general considerations, the epidemiologic transition, risk factors, and impact of urbanization. Circulation 2001;104:2746-2753.
3. Yusuf S, Reddy S, Ôunpuu S, et al. Global burden of cardiovascular diseases: Part II: variations in cardiovascular disease by specific ethnic groups and geographic regions and prevention strategies. Circulation 2001;104:2855-2864.
4. Sanderson JE, Mayosi B, Yusuf S, et al. Global burden of cardiovascular disease. Heart 2007;93:1175.
5. Rose G. Epidemiology of Atherosclerosis. BMJ: British Medical Journal: International Edition 1991;303:1537-1539.
6. Herrington W, Lacey B, Sherliker P, et al. Epidemiology of atherosclerosis and the potential to reduce theglobal burden of atherothrombotic disease. Circulation research 2016;118:535-546.
7. Norgren L, Hiatt WR, Dormandy JA, et al. Inter-society consensus for the management of peripheral arterial disease (TASC II). Journal of vascular surgery 2007;45:S5-S67.
8. Criqui MH, Aboyans V. Epidemiology of peripheral artery disease. Circulation research 2015;116:1509-1526.
9. Smith GD, Shipley MJ, Rose G. Intermittent claudication, heart disease risk factors, and mortality. The Whitehall Study. Circulation 1990;82:1925-1931.

10. Lane R, Harwood A, Watson L, et al. Exercise for intermittent claudication. The Cochrane Library 2017.
11. Kannel W, McGee D. Update on some epidemiologic features of intermittent claudication: theFramingham Study. Journal of the American Geriatrics Society 1985;33:13-18.
12. Virag R, Zwang G, Dermange H, et al. Vasculogenic impotence: a review of 92 cases with 54 surgical operations. Vascular Surgery 1981;15:9-17.
13. Seol SH. Leriche Syndrome. Journal of Medicine 2017;18:128.
14. Halperin JL. Evaluation of patients with peripheral vascular disease. Thrombosis research2002;106:V303-V311.
15. Lambert M, Belch J. Medical management of critical limb ischaemia: where do we stand today? Journalof internal medicine 2013;274:295-307.
16. Ouriel K. Peripheral arterial disease. The lancet 2001;358:1257-1264.
17. Rutherford RB, Baker JD, Ernst C, et al. Recommended standards for reports dealing with lowerextremity ischemia: revised version. Journal of vascular surgery 1997;26:517-538.
18. Fontaine R, Kim M, Kieny R. Surgical treatment of peripheral circulation disorders. Helvetica chirurgicaacta 1954;21:499-533.
19. Jaff MR, White CJ, Hiatt WR, et al. An update on methods for revascularization and expansion of the TASC lesion classification to include below-the-knee arteries: a supplement to the Inter-Society Consensus for the Management of Peripheral Arterial Disease (TASC II). Annals of vascular diseases 2015;8:343-357.
20. Treiman GS, Oderich GS, Ashrafi A, et al. Management of ischemic heel ulceration and gangrene: an evaluation of factors associated with successful healing. Journal of vascular surgery 2000;31:1110-1118.
21. Federman DG, Ladiiznski B, Dardik A, et al. Wound healing society 2014 update on guidelines for arterial ulcers. Wound Repair and Regeneration 2016;24:127-135.
22. Silva LR, Fernandes GM, Morales NU, et al. Results of One-Stage or Staged Amputations of LowerLimbs Consequent to Critical Limb Ischemia and Infection. Annals of vascular surgery 2018;46:218-225. 23.Alavi A, Sibbald RG, Phillips TJ, et al. What's new: Management of venous leg ulcers: Treating venous leg ulcers. Journal of the American Academy of Dermatology 2016;74:643-664.
24. Bergqvist D, Lindholm C, Nelzén O. Chronic leg ulcers: the impact of venous disease. Journal of vascular surgery 1999;29:752-755.
25. Singer AJ, Tassiopoulos A, Kirsner RS. Evaluation and management of lower-extremity ulcers. New England Journal of Medicine 2017;377:1559-1567.
26. Paquette D, Falanga V. Leg ulcers. Clinics in geriatric medicine 2002;18:77-88.
27. Phillips TJ, Dover JS. Leg ulcers. Journal of the American Academy of Dermatology 1991;25:965-987.
28.Abbade LP, Lastoria S, de Almeida Rollo H. Venous ulcer: clinical characteristics and risk factors. International journal of dermatology 2011;50:405-411.
29. Ferreira AM, Bogamil DD, Tormena PC. O enfermeiro e o tratamento de feridas: em busca da autonomiado cuidado. Arq Ciênc Saúde 2008;15:105-9.
30. Grey JE, Harding KG, Enoch S. Venous and arterial leg ulcers. BmJ 2006;332:347-350.
31. Sprengers RW, Lips DJ, Moll FL, et al. Progenitor cell therapy in patients with critical limb ischemia without surgical options. Annals of surgery 2008;247:411-420.

32. Isaac C, de Ladeira PRS, do Rêgo FMP, et al. Processo de cura das feridas: cicatrização fisiológica. Revista de Medicina 2010;89:125-131.
33. Diegelmann RF, Evans MC. Wound healing: an overview of acute, fibrotic and delayed healing. FrontBiosci 2004;9:283-289.
34. Barrientos S, Stojadinovic O, Golinko MS, et al. Growth factors and cytokines in wound healing. Woundrepair and regeneration 2008;16:585-601.
35. Velnar T, Bailey T, Smrkolj V. The wound healing process: an overview of the cellular and molecular mechanisms. Journal of International Medical Research 2009;37:1528-1542.
36. Schreml S, Szeimies R, Prantl L, et al. Oxygen in acute and chronic wound healing. British Journal of Dermatology 2010;163:257-268.
37. Tokuda Y, Crane S, Yamaguchi Y, et al. The levels and kinetics of oxygen tension detectable at the surface of human dermal fibroblast cultures. Journal of cellular physiology 2000;182:414-420.
38. RAZIYEVA, Kamila et al. Immunology of acute and chronic wound healing. Biomolecules, v. 11, n. 5, p. 700, 2021
39. Grazul-Bilska AT, Johnson ML, Bilski JJ, et al. Wound healing: the role of growth factors. Drugs Today (Barc) 2003;39:787-800.
40. Singer AJ, Clark RA. Cutaneous wound healing. New England journal of medicine 1999;341:738-746.41.Dhivya S, Padma VV, Santhini E. Wound dressings–a review. BioMedicine 2015;5.
42. Fan K, Tang J, Escandon J, et al. State of the art in topical wound-healing products. Plastic andreconstructive surgery 2011;127:44S-59S.
43. George Broughton I, Janis JE, Attinger CE. A brief history of wound care. Plastic and reconstructive surgery 2006;117:6S-11S.
44. Fonder MA, Lazarus GS, Cowan DA, et al. Treating the chronic wound: a practical approach to the care of nonhealing wounds and wound care dressings. Journal of the American Academy of Dermatology 2008;58:185-206.
45. Goossens A, Cleenewerck M. New wound dressings: classification, tolerance. European journal of dermatology: EJD 2010;20:24.
46. Bakker K, Apelqvist J, Schaper NC, et al. Practical guidelines on the management and prevention of the diabetic foot 2011. Diabetes/metabolism research and reviews 2012;28:225-231.
47. Futrega K, King M, Lott WB, et al. Treating the whole not the hole: necessary coupling of technologiesfor diabetic foot ulcer treatment. Trends in molecular medicine 2014;20:137-142.
48. Naruse K, Hamada Y, Nakashima E, et al. Therapeutic Neovascularization Using Cord Blood–Derived Endothelial Progenitor Cells for Diabetic Neuropathy. Diabetes 2005;54:1823-1828.
49. Strem BM, Hicok KC, Zhu M, et al. Multipotential differentiation of adipose tissue-derived stem cells. The Keio journal of medicine 2005;54:132-141.
50. Lee RH, Kim B, Choi I, et al. Characterization and expression analysis of mesenchymal stem cells from human bone marrow and adipose tissue. Cellular physiology and biochemistry 2004;14:311-324.
51. Winkler IG, Wiercinska E, Barbier V, et al. Mobilization of hematopoietic stem cells with highest self- renewal

by G-CSF precedes clonogenic cell mobilization peak. Experimental hematology 2016;44:303-314. e1.

52. Baksh D, Song L, Tuan R. Adult mesenchymal stem cells: characterization, differentiation, and application in cell and gene therapy. Journal of cellular and molecular medicine 2004;8:301-316.
53. Dominici M, Le Blanc K, Mueller I, et al. Minimal criteria for defining multipotent mesenchymal stromal cells. The International Society for Cellular Therapy position statement. Cytotherapy 2006;8:315-317.
54. Zuk PA, Zhu M, Ashjian P, et al. Human adipose tissue is a source of multipotent stem cells. Molecular biology of the cell 2002;13:4279-4295.
55. Bourin P, Bunnell BA, Casteilla L, et al. Stromal cells from the adipose tissue-derived stromal vascular fraction and culture expanded adipose tissue-derived stromal/stem cells: a joint statement of the International Federation for Adipose Therapeutics and Science (IFATS) and the International Society for Cellular Therapy (ISCT). Cytotherapy 2013;15:641-648.
56. Rodbell M. The removal and metabolism of chylomicrons by adipose tissue in vitro. Journal of Biological Chemistry 1960;235:1613-1620.
57. Dai R, Wang Z, Samanipour R, et al. Adipose-derived stem cells for tissue engineering and regenerative medicine applications. Stem cells international 2016;2016.
58. Gao F, Chiu S, Motan D, et al. Mesenchymal stem cells and immunomodulation: current status and future prospects. Cell death & disease 2017;7:e2062.
59. Wang Y, Chen X, Cao W, et al. Plasticity of mesenchymal stem cells in immunomodulation: pathologicaland therapeutic implications. Nature immunology 2014;15:1009.
60. Glenn JD, Whartenby KA. Mesenchymal stem cells: emerging mechanisms of immunomodulation and therapy. World journal of stem cells 2014;6:526.
61. Tateishi-Yuyama E, Matsubara H, Murohara T, et al. Therapeutic angiogenesis for patients with limb ischaemia by autologous transplantation of bone-marrow cells: a pilot study and a randomised controlled trial. The Lancet 2002;360:427-435.
62. Kawamura A, Horie T, Tsuda I, et al. Clinical study of therapeutic angiogenesis by autologous peripheralblood stem cell (PBSC) transplantation in 92 patients with critically ischemic limbs. Journal of Artificial Organs 2006;9:226-233.
63. Horie T, Onodera R, Akamastu M, et al. Long-term clinical outcomes for patients with lower limb ischemia implanted with G-CSF-mobilized autologous peripheral blood mononuclear cells. Atherosclerosis 2010;208:461-466.
64. Jiang X, Zhang H, Teng M. Effectiveness of autologous stem cell therapy for the treatment of lower extremity ulcers: a systematic review and meta-analysis. Medicine 2016;95.
65. Tsuji W, Rubin J, Marra K. Adipose derived stem cells: implications in tissue regeneration. World J StemCells 2014;26:312-321.
66. Dainesi SM. A metodologia probe pode ser considerada uma alternativa aos estudos randomizados duplo-cegos? Revista da Associação Médica Brasileira 2010; 56.2:132.
67. Agência Nacional de Vigilância Sanitária (Brasil). Resolução da Diretoria Colegiada - RDC Nº 214, de 7de

fevereiro de 2018. Dispõe sobre as Boas Práticas em Células Humanas para Uso Terapêutico e pesquisa clínica, e dá outras providências. Diário Oficial da União nº 36, de 22 de fevereiro de 2018.

68. Agência Nacional de Vigilância Sanitária (Brasil). Resolução da Diretoria Colegiada - RDC Nº 260, de 21 de dezembro de 2018. Dispõe sobre as regras para a realização de ensaios clínicos com produto de terapia avançada investigacional no Brasil, e dá outras providências. Diário Oficial da União nº249 de 28dez 2018.

69. Bustin, S. a, Benes, V., Garson, J. a, Hellemans, J., Huggett, J., Kubista, M., … Wittwer, C. T. (2009). The MIQE guidelines: minimum information for publication of quantitative real-time PCR experiments. *Clinical Chemistry*, *55*(4), 611–622. https://doi.org/10.1373/clinchem.2008.112797

70. Fleige, S., & Pfaffl, M. W. (2006). RNA integrity and the effect on the real-time qRT-PCR performance. *Molecular Aspects of Medicine*, *27*(2–3), 126–139. https://doi.org/10.1016/j.mam.2005.12.003

71. Ye, J., Coulouris, G., Zaretskaya, I., Cutcutache, I., Rozen, S., & Madden, T. L. (2012). Primer-BLAST:a tool to design target-specific primers for polymerase chain reaction. *BMC Bioinformatics*, *13*, 134. https://doi.org/10.1186/1471-2105-13-134

72. Livak, K. J., & Schmittgen, T. D. (2001). Analysis of relative gene expression data using real-time quantitative PCR and the 2(-Delta Delta C(T)) Method. *Methods (San Diego, Calif.)*, *25*(4), 402–408. https://doi.org/10.1006/meth.2001.1262

18. Anexos e Apêndice

Anexo I – Questionário de estado de saúde SF-36V2

QUESTIONÁRIO DE ESTADO DE SAÚDE (SF-36V2)

INSTRUÇÕES: As questões que se seguem pedem-lhe opinião sobre a sua saúde, a forma como se sente e sobre a sua capacidade de desempenhar as actividades habituais.

Pedimos que leia com atenção cada pergunta e responda o mais honestamente possível. se não tiver a certeza sobre a resposta a dar, dê-nos a que achar mais apropriada e, se quiser, escreva um comentário a seguir à pergunta.

Para as perguntas 1 e 2, por favor coloque um círculo no número que melhor descreve a sua saúde.

1. Em geral, diria que a sua saúde é:

Óptima	Muito boa	Boa	Razoável	Fraca
1	2	3	4	5

2. Comparando com o que acontecia há um ano, como descreve o seu estado geral actual:

Muito melhor	Com algumas melhoras	Aproximadamente igual	Um pouco pior	Muito pior
1	2	3	4	5

3. As perguntas que se seguem são sobre actividades que executa no seu dia-a-dia.
Será que a sua saúde o/a limita nestas actividades? Se sim, quanto?

(Por favor assinale com um círculo um número em cada linha)

	Sim, muito limitado/a	Sim, um pouco limitado/a	Não, nada limitado/a
a. **Actividades violentas**, tais como correr, levantar pesos, participar em desportos extenuantes.......	1	2	3
b. **Actividades moderadas**, tais como deslocar uma mesa ou aspirar a casa..	1	2	3
c. Levantar ou pegar nas compras da mercearia.....	1	2	3
d. Subir **vários** lanços de escadas.............................	1	2	3
e. Subir **um** lanço de escadas......................................	1	2	3
f. Inclinar-se, ajoelhar-se ou baixar-se....................	1	2	3
g. Andar **mais de 1 Km**..	1	2	3
h. Andas **várias** centenas de metros.........................	1	2	3
i. Andar **uma** centena de metros............................	1	2	3
j. Tomar banho ou vestir-se sozinho/a..................	1	2	3

**4. Durante as últimas 4 semanas teve, no seu trabalho ou actividades diárias, algum dos problemas apresentados a seguir como consequência do seu estado de saúde físico?**					
Quanto tempo, nas **últimas quatro semanas...**	**Sempre**	**A maior parte do tempo**	**Algum tempo**	**Pouco tempo**	**Nunca**
a. Diminuiu o **tempo gasto** a trabalhar ou outras actividades	1	2	3	4	5
b. Fez **menos** do que queria?..	1	2	3	4	5
c. Sentiu-se limitado/a no tipo de trabalho ou outras actividades	1	2	3	4	5
d. Teve **dificuldade** em executar o seu trabalho ou outras actividades (por exemplo, foi preciso mais esforço).......	1	2	3	4	5

**5. Durante as últimas 4 semanas, teve com o seu trabalho ou com as suas actividades diárias, algum dos problemas apresentados a seguir devido a quaisquer problemas emocionais (tal como sentir-se deprimido/a ou ansioso/a)?**					
Quanto tempo, nas **últimas quatro semanas...**	**Sempre**	**A maior parte do tempo**	**Algum tempo**	**Pouco tempo**	**Nunca**
a. Diminuiu o **tempo gasto** a trabalhar ou outras actividades	1	2	3	4	5
b. Fez **menos** do que queria?..	1	2	3	4	5
c. Executou o seu trabalho ou outras actividades **menos** cuidadosamente do que era costume......................................	1	2	3	4	5

Para cada uma das perguntas 6, 7 e 8, por favor ponha um círculo no número que melhor descreve a sua saúde.

6. Durante as últimas 4 semanas, em que medida é que a sua saúde física ou problemas emocionais interferiram no seu relacionamento social normal com a família, amigos, vizinhos ou outras pessoas?

**Absolutamente nada**	**Pouco**	**Moderadamente**	**Bastante**	**Imenso**
1	2	3	4	5

7. Durante as últimas 4 semanas teve dores?					
Nenhumas	Muito fracas	Ligeiras	Moderadas	Fortes	Muito fortes
1	2	3	4	5	6

8. Durante as últimas 4 semanas, de que forma é que a dor interferiu com o seu trabalho normal (tanto o trabalho fora de casa como o trabalho doméstico)?				
Absolutamente nada	Pouco	Moderadamente	Bastante	Imenso
1	2	3	4	5

9. As perguntas que se seguem pretendem avaliar a forma como se sentiu e como lhe correram as coisas nas últimas quatro semanas.
Para cada pergunta, coloque por favor um círculo à volta do número que melhor descreve a forma como se sentiu.
Certifique-se que coloca um círculo em cada linha.

Quanto tempo, nas últimas quatro semanas…	Sempre	A maior parte do tempo	Algum tempo	Pouco tempo	Nunca
a. Se sentiu cheio/a de vitalidade?.............	1	2	3	4	5
b. Se sentiu muito nervoso/a?....................	1	2	3	4	5
c. Se sentiu tão deprimido/a que nada o/a animava?..	1	2	3	4	5
d. Se sentiu calmo/a e tranquilo/a?...........	1	2	3	4	5
e. Se sentiu com muita energia?...............	1	2	3	4	5
f. Se sentiu deprimido/a?.........................	1	2	3	4	5
g. Se sentiu estafado/a?...........................	1	2	3	4	5
h. Se sentiu feliz?......................................	1	2	3	4	5
i. Se sentiu cansado/a?............................	1	2	3	4	5

7. Durante as últimas 4 semanas teve dores?

Nenhumas	Muito fracas	Ligeiras	Moderadas	Fortes	Muito fortes
1	2	3	4	5	6

8. Durante as últimas 4 semanas, de que forma é que a dor interferiu com o seu trabalho normal (tanto o trabalho fora de casa como o trabalho doméstico)?

Absolutamente nada	Pouco	Moderadamente	Bastante	Imenso
1	2	3	4	5

9. As perguntas que se seguem pretendem avaliar a forma como se sentiu e como lhe correram as coisas nas últimas quatro semanas.
Para cada pergunta, coloque por favor um círculo à volta do número que melhor descreve a forma como se sentiu.
Certifique-se que coloca um círculo em cada linha.

Quanto tempo, **nas últimas quatro semanas...**	**Sempre**	**A maior parte do tempo**	**Algum tempo**	**Pouco tempo**	**Nunca**
a. Se sentiu cheio/a de vitalidade?.............	1	2	3	4	5
b. Se sentiu muito nervoso/a?....................	1	2	3	4	5
c. Se sentiu tão deprimido/a que nada o/a animava?..	1	2	3	4	5
d. Se sentiu calmo/a e tranquilo/a?..........	1	2	3	4	5
e. Se sentiu com muita energia?................	1	2	3	4	5
f. Se sentiu deprimido/a?.........................	1	2	3	4	5
g. Se sentiu estafado/a?...........................	1	2	3	4	5
h. Se sentiu feliz?......................................	1	2	3	4	5
i. Se sentiu cansado/a?............................	1	2	3	4	5

10. Durante as últimas quatro semanas, até que ponto é que a sua saúde física ou problemas emocionais limitaram a sua actividade social (tal como visitar amigos ou familiares próximos)?

Sempre	A maior parte do tempo	Algum tempo	Pouco tempo	Nunca
1	2	3	4	5

11. Por favor, diga em que medida são verdadeiras ou falsas as seguintes afirmações.
Ponha um círculo para cada linha.

	Absolutamente verdade	Verdade	Não sei	Falso	Absolutamente falso
a. Parece que adoeço mais facilmente do que os outros.................................	1	2	3	4	5
b. Sou tão saudável como qualquer outra pessoa.......................................	1	2	3	4	5
c. Estou convencido/a que a minha saúde vai piorar.................................	1	2	3	4	5
d. A minha saúde é óptima....................	1	2	3	4	5

Anexo II - Escala Visual Analógica (EVA) para avaliação do nível de dor

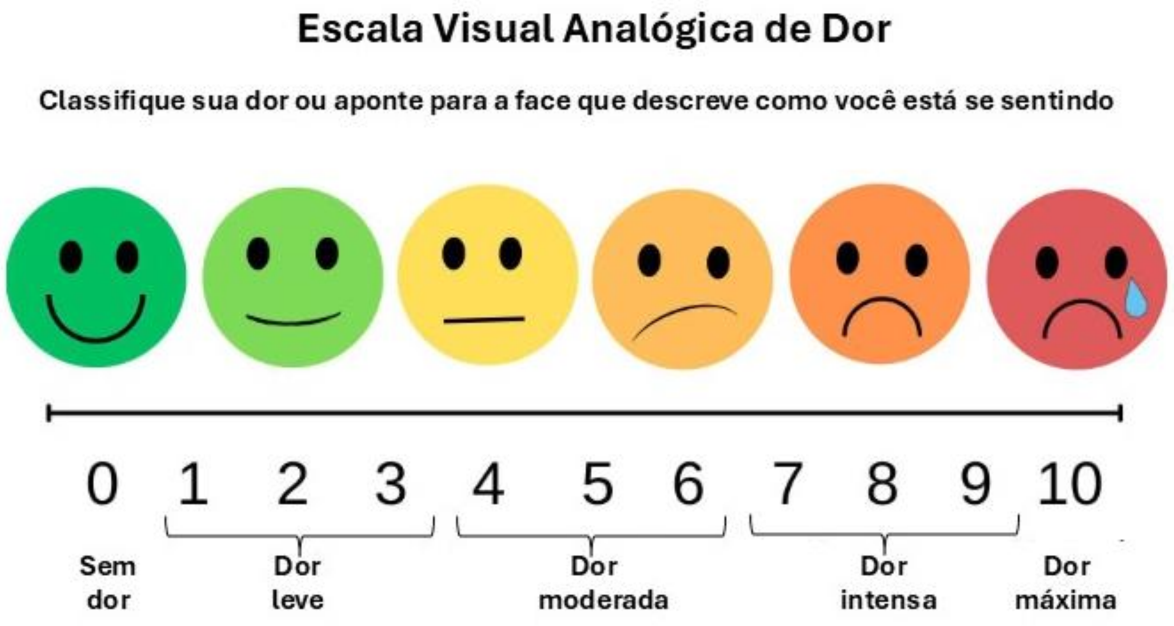

Anexo III – Questionário EQ-5D

Para cada um dos tópicos abaixo, marque apenas UMA alternativa que melhor descreve sua saúde HOJE

MOBILIDADE

- ☐ Não tenho problemas para caminhar
- ☐ Tenho algum problema para caminhar
- ☐ Tenho problemas moderados para caminhar
- ☐ Tenho problemas graves para caminhar
- ☐ Tenho problemas extremos para caminhar

CUIDADOS PESSOAIS

- ☐ Não tenho problemas para me vestir ou tomar banho
- ☐ Tenho algum problema para me vestir ou tomar banho
- ☐ Tenho problemas moderados para me vestir ou tomar banho
- ☐ Tenho problemas graves para me vestir ou tomar banho
- ☐ Tenho problemas extremos para me vestir ou tomar banho

ATIVIDADES USUAIS (trabalho, estudo, atividades domiciliares, familiares, lazer)

- ☐ Não tenho problemas para realizar minhas atividades usuais
- ☐ Tenho algum problema para realizar minhas atividades usuais
- ☐ Tenho problemas moderados para realizar minhas atividades usuais
- ☐ Tenho problemas graves para realizar minhas atividades usuais
- ☐ Tenho problemas extremos para realizar minhas atividades usuais

DOR/DESCONFORTO

- ☐ Não tenho dor ou desconforto
- ☐ Tenho algumas dores ou desconforto
- ☐ Tenho dores ou desconforto moderados
- ☐ Tenho dores ou desconforto graves
- ☐ Tenho dores ou desconforto extremos

ANSIEDADE/DEPRESSÃO

- ☐ Não sou ansioso/deprimido
- ☐ Sou um pouco ansioso/deprimido
- ☐ Sou moderadamente ansioso/deprimido
- ☐ Sou muito ansioso/deprimido
- ☐ Sou extremamente ansioso/deprimido

1. Nós gostaríamos de saber como está sua saúde HOJE.

2. Esta escala está marcada de 0 a 100.

3. 100 significa a melhor saúde que você pode imaginar.

0 significa a pior saúde que você pode imaginar.

4. Marque um X na escala para indicar como está sua saúde HOJE.

5. Agora, por favor, anote o número que você marcou na escala na caixa abaixo.

SUA SAÚDE HOJE = ☐

A melhor saúde que você pode imaginar

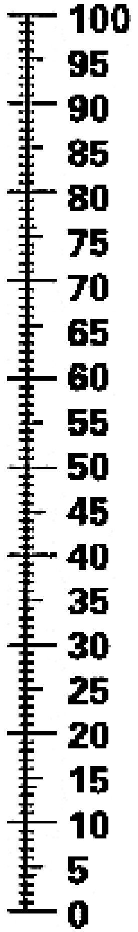

A pior saúde que você pode imaginar

Apêndice I - TCLE

CONSENTIMENTO LIVRE E ESCLARECIDO (TCLE)RESOLUÇÃO 466/2012

CONVIDO, o Senhor(a) para participar do Projeto de Pesquisa intitulado "**ESTUDO CLÍNICO RANDOMIZADO, PROSPECTIVO, CONTROLADO, UNICÊNTRICO E ABERTO DE FASE II PARA AVALIAR A SEGURANÇA E A EFICÁCIA DA TERAPIA COM CÉLULAS-TRONCO MESENQUIMAIS AUTÓLOGAS PARA ÚLCERAS EM PORTADORES DE ISQUEMIA CRÍTICA DE MEMBROS INFERIORES – ESTUDO STEM-CELL I**", que será desenvolvido por mim Prof. Dr. Matheus Bertanha, Cirurgião Vascular, com a colaboração dos Profs. Drs. Marcone Lima Sobreira, Cirurgião Vascular.

Estou estudando a doença arterial periférica (DAP), responsável pela diminuição da circulação sanguínea dos membros inferiores e causadora das suas feridas que não cicatrizam. Para que eu possa ter um resultado nesse momento preciso coletar 20ml do seu sangue que será utilizado para realização dos seguintes exames laboratoriais (hemograma completo, ureia, creatinina, sódio, potássio, glicose em jejum, hemoglobina glicada, CPK, TGO, TGP, PCR, bilirrubinas, PTF, coagulograma, Ácido Úrico, Colesterol total,HDL, LDL, Triglicérides, HIV tipo I e II, HTLV tipo I e II e Hepatites B e C). Isso será realizado em três momentos durante o estudo ou mais vezes se considerarmos necessário, sendo a primeira coleta antes de qualquer procedimento do estudo, para averiguarmos as condições gerais de sua saúde. O risco com a coleta de sangue será a picadinha da agulha e uma manchinha roxa que desaparecerá bem rapidamente.

Você será incluído no estudo para participar de forma aleatória de dois possíveis grupos de tratamento e não será possível fazer a mudança de um grupo para o outro. Um dos grupos será o grupo de pacientes que receberão tratamento apenas com curativos com Hidrogel, sendo que se você participar neste grupo, você terá uma rotina de consultas para comparecer e deverá seguir corretamente as instruções que serão dadas mais à frente. Da mesma forma, você poderá participar do grupo que será submetido ao tratamento com células-tronco mesenquimais (CTM) que serão retiradas de você mesmo(a). Dois procedimentos terão que ser realizados. No primeiro, será agendada uma pequena cirurgia para coleta de um pequeno fragmento de pele e gordura da sua barriga com 2cm de diâmetro, que será realizada com anestesia local e sem necessidade de internação hospitalar. Esse material será levado para o nosso laboratório e dele vamos retirar as CTM e alimenta-las para que aumentem em número. Se você participar deste grupo, os riscos desse primeiro procedimento são pequenos, tais como: infecção, não cicatrização da ferida, alergia ao medicamento anestésico, entre outros. O segundo procedimento será realizado três ou quatro semanas após a coleta da gordura da barriga. Mais uma vez, precisaremos de um pouco do seu sangue, em torno de 50ml, já na hora que você chegar para ser atendido(a), para fazermos os preparos das suas células. Assim que as células estiverem preparadas, em torno de 3 horas depois do atendimento, você será submetido(a) a um procedimento cirúrgico com anestesia por bloqueio local ou por raquianestesia e alguma sedação para você não sentir dor, que será escolhido pelo anestesiologista de forma que seja o mais seguro para você. Depoisdas preparações cirúrgicas, será feita uma boa limpeza da ferida, a injeção das suas CTM por pequenas punções nas bordas da ferida e um curativo contendo CTM também será colocado na ferida. Você receberá orientações de cuidados com esse curativo. Neste momento, os riscos relacionados serão: os riscos anestésicos; alergias aos medicamentos, além da possibilidade de não funcionamento da nova técnica. Você deve receber alta ainda neste mesmo dia.

Informo que o material biológico colhido do Senhor(a), CTM obtidas do tecido gorduroso da barriga,não será usado em sua totalidade, sendo que parte desse material será utilizado para controles rigorosos da sua qualidade e parte será armazenado na Faculdade de Medicina. Esse material será utilizado somente no tempo de vigência desta pesquisa (2 anos a partir da coleta), estritamente como foi explicado e após os dois anos será descartado.

Solicito também seu consentimento para consultar seu prontuário médico para coletar outras informações contidas em consultas feitas pelo (a) Senhor (a) e dados que possam ser relevantes a essa pesquisa

Além disso, o(a) Senhor(a) deverá comparecer em consultas que serão agendadas (7 dias, 30 dias, 60dias, 90 dias e 120 dias) para avaliação dos curativos. Nestas consultas você responderá alguns questionários para o acompanhamento da sua saúde, o que levará cerca de 15 minutos de duração, em cada consulta.

Informo também que, participando em qualquer dos dois grupos, alguns outros exames serão realizados, como ultrassonografia vascular com Doppler, Termografia da pele com uso de uma câmera fotográfica especial, aferição dos níveis de oxigênio e gás carbônico da pele por um eletrodo simples, o que vai demorar por volta de uma hora e será realizado em 3 consultas. A sua úlcera será fotografada apenas parauso na pesquisa ou para demonstração de resultado científico sem que o(a) senhor(a) seja identificado(a). Todos esses procedimentos não precisam de nenhum preparo e não devem expor você a nenhum risco adicional.

Seu benefício em participar da pesquisa será o de ter a chance de receber um novo tratamento com CTM retiradas de você mesmo(a) que pode vir a ajudar na melhora da sua circulação e aumentar as chances de cura da sua úlcera. Caso não houver benefício neste momento e de forma direta para o Senhor(a), esta pesquisa trará benefícios para futuros pacientes, após a coleta e conhecimento dos resultados dessa pesquisa.

Fique ciente de que sua participação neste estudo é voluntária e que mesmo após ter dado seu consentimento para participar da pesquisa, você poderá retirá-lo a qualquer momento, sem qualquer prejuízo na continuidade do seu tratamento.

Este Termo de Consentimento Livre e Esclarecido foi elaborado em 2 vias de igual teor, o qual 01via será entregue ao Senhor(a) devidamente rubricada, e a outra via será arquivada e mantida pelos pesquisadores por um período de 5 anos após o término da pesquisa.

Qualquer dúvida adicional você poderá entrar em contrato com o Comitê de Ética em Pesquisa através dos telefones (14) 3880-1608 ou 3880-1609. Ele funciona de 2ª a 6ª feira das 8.00 às 11.30 e das 14.00 às 17horas, na Chácara Butignolli s/nº em Rubião Júnior – Botucatu - São Paulo. Os dados de localização dos pesquisadores estão abaixo descritos.

Após terem sido sanadas todas minhas dúvidas a respeito deste estudo, CONCORDO EM PARTICIPAR de forma voluntária deste estudo, estando ciente que todos os meus dados estarão resguardados através do sigilo que os pesquisadores se comprometeram. Estou ciente que os resultados desseestudo poderão ser publicados em revistas científicas, sem, no entanto, que minha identidade seja revelada.

Botucatu,_______/_____/20_____.

____ _______________________________________

Pesquisador Participante da Pesquisa

Nome: Matheus Bertanha
Endereço: Av. Prof. Mário Rubens Guimarães Montenegro, s/n. Bairro: UNESP - Campus de Botucatu CEP 18618687 - Botucatu, SPPABX: (14) 3880-1001
Telefone: (14) 38801444
Email: matheusbertanha@fmb.unesp.br

CLINICAL STUDY TO EVALUATE THE SAFETY AND EFFICACY OF AUTOLOGOUS MESENCHYMAL STEM CELL THERAPY IN ULCERS OF PATIENTS WITH CRITICAL LIMB ISCHEMIA - STEM CELL STUDY I

STUDY STEM CELL I

Prof Dr. Matheus Bertanha

Botucatu

2023

IDENTIFICATION FROM THE PROPOSAL, PROPONENT AND INSTITUTION EXECUTOR	
**TITLE DO PROJECT**	CLINICAL STUDY TO EVALUATE THE SAFETY AND EFFICACY OF AUTOLOGOUS MESENCHYMAL STEM CELL THERAPY IN ULCERS OF PATIENTS WITH CRITICAL LIMB ISCHEMIA - STEM CELL STUDY I
**NAME DO PROPONENT**<br>**Lattes CNPq CVWork**<br>**Regime Position**<br><br>**Contacts**	MATHEUS BERTANHA<br>http://lattes.cnpq.br/4513014379461383RDIDP<br>Regime<br>Prof. Assist. Dr. from the Discipline of Surgery Vascular ofDept. Surgery and Orthopedics<br>matheusbertanha@gmail.com<br>matheus.bertanha@unesp.br<br>Telephone: 14 3880-1444<br>Cell phone: 14 997351711
**CPF**	259.258.048-48
**INSTITUTION EXECUTOR**	FACULTY OF MEDICINE FROM THE UNIVERSITY<br>STATE PAULISTA – CAMPUS OF BOTUCATU UNESP<br>CNPJ – 48.031.918/0019-53<br>Avenue Mario Rubens Guimaraes Montenegro s/n Rubiao Junior<br>CEP - 18618-687
**INSTITUTIONS/COMPANIES PARTNERS**	
**AREA**	AREA: SCIENCES OF HEALTH<br>Subarea: Medicine – Clinic Doctor – Angiology<br>Area of Knowledge CNPq – 4.01.01.01-0

DEADLINES OF EXECUTION DO PROJECT

Total duration: 48 months
Date of Start: 12/01/2023
Date of closing of study: 12/12/2027

Requested secrecy patent to the to analyze the content of this project.

RANDOMIZED, PROSPECTIVE, CONTROLLED, SINGLE-CENTER, PHASE I/II CLINICAL STUDY TO TO ASSESS THE SECURITY AND THE EFFECTIVENESS FROM THE THERAPY WITH STEM CELLS AUTOLOGOUS MESENCHYMAL IN ULCERS OF PATIENTS WITH CRITICAL LETHOLOGICAL ISCHEMIA MEMBERS LOWER – STEM STUDY CELL I

Abstract

Background: Peripheral arterial disease (PAD) can progress to critical limb ischemia (CLI) of the affected lower limb, characterized by pain at rest, ulcerations or gangrene, with a high risk of amputation. In this phase, the best treatment is arterial limb revascularization, but this is not always possible or even effective for promoting pain relief, healing of ulcers or preventing amputations, in addition to the high socioeconomiccost caused by the disease. Recent advances in cell therapy represent a promising supporting alternative for the treatment of PAD in cases where conventional alternatives have run out. **Objective** : To evaluate the safety and efficacy of cell therapy with expanded autologous mesenchymal stem cells in the treatment of patients with PAD with CLI and chronic arterial ulcers. **Methods** : An open randomized clinical study will be performed with 2 groups of 20 patients with CLI: in group 1, the fragment of abdominal fat tissue (10g) will be collected to obtain mesenchymal stem cells, which will be expanded and applied using subcutaneous perilesional injection in the affected lower limb, in addition to the application in the form of a personalized biological curative on the wounds. Group 2 will receive conventional treatment with a Hydrogel dressing with essential fatty acids. Periodic clinical evaluations, complementary exams and photographic record will be carried out. The main outcome of effectiveness will be partial or total wound healing. Safety outcomes will be monitored for infections, gangrene, amputations and deaths. Participants will be monitored for 120 days. Major amputation cases will not be included. An independent external evaluator and blind to the groups will evaluate the results. It is an innovative procedure with high impact and financial return for SUS, in view of the high prevalence of the disease and the high socioeconomic impact of the disease when it progress to limb amputation.

Keywords: Cell and Tissue-Based Therapy, Wound Healing, Peripheral Arterial Disease, Stem Cells, Clinical Trial Phase II, Amputation.

INDEX

Summary 4
Abstract 5
LIST OF TABLES 9
LIST OF FIGURES 9
LIST OF TABLES 10
1. BASIS AND CONTEXTUALIZATION 13
1.1 Ulcers 15
1.2 Ulcers in Illness Arterial Peripheral and Ischemia review 15
1.3 Mechanisms of healing and chronification of the wounds 16
1.4 Dressings or covers 18
1.5 Stem cells mesenchymal 18
1.6 State current from the therapy cell phone to Illness Arterial Peripheral and Ischemia Review 19
2. JUSTIFICATION 20
3. OBJECTIVES 21
3.1 Objectives Primary 21
3.2 Objectives Secondary 22
4. DESIGN DO STUDY 22
4.1. Description of design of study 22
4.2. Justification to the design of study 22
4.3 Location of Study and ethics 23
4.4 Calculation sample and treatment statistical of the results 23
4.4.1 Size from the Sample and Recruitment 23
4.4.2 Proposed groups node rehearsal clinical 23
4.5 Criteria of Eligibility 24
4.5.1 Criteria of Inclusion 24
4.5.2 Criteria of Exclusion 25
4.6 Randomization 26
4.6.1 Methodology and guarantee of quality of results node study open 27
4.7 Procedures of study clinical us groups proposed 27
4.7.1 Collection of Tissue Adipose to obtain stem cells mesenchymal autologous to use in Group 1 ..27
4.7.2 Processing from the sample of Tissue Adipose 28
4.7.3 Procedures of Expansion Cell 28
4.7.4 Control of quality cell phone 29
4.7.5 Microbiological control from the culture cell phone 29
4.7.6 Control of quality of the stem cell 29
4.7.7 Assessment of expression genetic put RT-qPCR 30
4.8 Justification to amount of cells (Dose) 31
4.9 Preparation, storage and procedures to dispensation of product 31
4.10 Procedure of application of product 32
4.11 Control quality of the study 33
4.11.1 Procedures of sampling 33
4.11.2 Requirements to acceptance of batch of materials, reagents and products to diagnosis *in vitro* used in manufacturing of product and us control processes of quality 35
4.11.2 Safety and quality requirements for advanced therapy product release investigational to administration 35
4.11.3 Characterization of the active component, including, where applicable, its identity, quantity, purity, viability, potency, genetic stability and sterility, and should describe the methodologies analytics maids 38
4.12 Reactions or risks relative to the treatment 38
4.13 Follow-up, Assessments, Procedures and Schedule of Visits 38
4.13.1 Reviews Clinics 38
4.13.2 Assessment from the ulcer: 40
4.13.3 Reviews Laboratory 40
4.13.4 Dressings 41

4.13.5 Evaluation of Follow-up 41

4.13.6 Criteria of discontinuation node study 43
4.14 Outcomes 43
4.14.1 Outcome primary 43
4.14.2 Outcome secondary 43
4.15 Collect and monitoring of the data 44
4.15.1 Collect and monitoring of the data of study clinical 44
4.15.2 Flat of Monitoring of study clinical 47
4.15.3 Report and Management of Events Adverses 48
4.16 Committee Independent of Monitoring of Security 49
4.17 Process of report of security 49
4.18 Flat of analysis interim 49
4.19 Medications concomitant 50
4.20 Criteria of discontinuity of the participants 50
4.21 Methods statisticians 50
5. RESPONSIBILITIES 51
5.1 Good Practices Clinics 51
5.2 Principles Ethical 51
5.2.1 Laws and Regulations 51
5.2.2 Consent Free and Enlightened 51
5.2.3 Committee of Ethics in Search (CEP)/CONEP 51
5.2.4 Amendment to the protocol of study clinical 52
5.2.5 Deviations to the Protocol 52
5.3 Definition of the Source data 52
5.3.1 Retention of record node center of study - File of documents 52
5.4 Compensation of Insurance 53
5.5 Publications and Communications 53
5.6 Control of Quality and Guarantee of Quality 53
6. RESULTS EXPECTED AND YOUR RELEVANCE TO YOU SICK AND THE SUS 54
6.1 Results expected 54
6.2 Alignment with policies public of health 55
6.3 Impacts to the sus 56
7. Cost-Effectiveness 56
8. Impacts and contributions to the complex economic-industrial from the health 57
9. CHARACTER INNOVATIVE DO PROJECT 58
9.1 Study of feasibility patent 58
10. PLANNING FROM THE MANAGEMENT OF RISKS: 59
11. PROPERTY INTELLECTUAL 59
12. INSTITUTIONAL INFRASTRUCTURE AND OPERATIONAL TECHNICAL CAPACITY OF TEAM DO STUDY 60
12.1. Infrastructure institutional 60
12.1.1 Unit of Search Clinic from the FMB/UNESP - UPECLIN 60
12.1.2 Laboratories of Search of the Blood center of Botucatu 61
12.2 Capacity intellectual and technique operational from the team of study 62
12.2.1 Brief compilation of the research activities carried out, considered by the applicant as more relevant, indicating the production generated put they by 2019 62
12.2.2 Brief compilation of research activities carried out, considered by the collaborator main to the more relevant, indicating the production generated put they until 2019 64
13. FLAT OF WORK 66
13.1 Identification of object to be executed - Flat of work 66
13.2 Preparation, preparation and shipping of dossiers regulatory 68
13.3 Start from the execution of study clinical 68
13.4. Execution of study clinical 68
13.5 End of period of inclusion of participants in the study 68
13.6 Closing from the execution of study clinical 68
13.7 Management of the data and statistic 69
13.8 Preparation and shipping of reports 69

13.9 Participation in events scientific disclosure of the results 69
14. BUDGET DETAILED 69

15. SUMMARY OF INVESTMENTS 74
16. COUNTERPART FROM THE INSTITUTION 74
17. REFERENCES BIBLIOGRAPHICAL 74
18. Annexes and Appendix 79

LIST OF TABLES

Table 1. Classification of Rutherford and Fontaine to DAP 14
Table 2. Criteria of inclusion 24
Table 3. Criteria of exclusion 25
Table 4 . Standard of accordance to stem cells mesenchymal 30
Table 5. Timeline of Reviews and Visits 41
Table 6. Schedule 66

LIST OF FIGURES

Figure 1. Flow Monitoring of the data from the search clinic with therapy cell phone advanced proposal in thisstudy. 47

LIST OF PICTURES

Frame 1: Management of risks relativ the execution of present study 57

1. BASIS AND CONTEXTUALIZATION

The atherosclerosis and one illness cardiovascular chronicle and progressive what if manifests more often, from adulthood onwards, being characterized by chronic inflammation of the tunica intima of the large and medium-sized arteries (atherosclerosis), occurring due to the accumulation and oxidation of lipoproteins in the arterial wall, causing a series of multifocal lesions, the most common, the atheroma plaque, that grows into the lumen, which may restrict flow to the distal segments by partial narrowing (stenoses) or occlusion total from the light. [1] AND considered the main cause of mortality of the countries industrialized. [1-4] With the reduction of blood flow to the affected limb, there is a decrease in contribution of nutrients and oxygen to the fabrics and, consequently, ischemia. [5, 6]

Peripheral arterial disease (PAD) is the manifestation of atherosclerotic disease that affects the circulation arterial peripheral, mainly of the members inferior, with frequency estimated in studies international10% to 12% in the adult population and 20% of the population over 75 years of age, with a predominance of the male sex male. [7] Overall, the estimated incidence of critical limb ischemia (CLI) is 500 to 1,000 members inferior affected put million of inhabitants put year [7] Between those sick, the index of amputationprimary varies from 10% to 40%, characterizing a major public health problem. As for the factors of risk, are considered largest: diabetes *mellitus* type 2 (DMT2), smoking, the hypertension arterial systemic (HAS) and the hyperlipidemia, responsible put 80 the 90% of the illnesses cardiovascular. [7] Others factors of risk he has been involved as active in the genesis of process atherosclerotic, but due to theits possibly secondary role or the need for more controlled studies that indicate its real paper in the atherogenesis they are considered factors of risk minors; between they: hyperhomocysteinemia, hyperfibrinogenia, inflammatory factors, factors genetic, infectious agents, alterations in metabolism calcium, hypercoagulable states, metalloproteinase imbalance, renal failure and stress oxidative. [8, 9]

The signs and symptoms of PAD result from limited blood flow in the arteries of the limbs. inferior, imposed put one stenosis criticism or occlusion arterial, secondary the training from the plate atherosclerotic, clinically manifesting as intermittent claudication, a symptom pathognomonic initial of DAP, characterized by the patient as the sensation of muscular fatigue, numbness of the limb, tightness in the calf, cramp or paralysis in certain groupings muscular of member lower, triggered for the exercise physical and improving with the your interruption, normally limiting to the walks the specific distances. [10, 11] Symptoms may also appear in the thigh or buttocks, and be associated with sexual impotence, depending on the artery and the extent of the injury. [12, 13] In the clinical evaluation, they manifest decreased or absent pulses and external signs of ischemia, such as cyanosis, pallor, skin fragility, brittle nails, lack of hair and ulcers, characterized as trophic signs of ischemic disease peripheral. [14] With the evolution of the disease the sick go limiting your walk the distances each time minors,

with intervals of time to recovery each time bigger.

At the end of the disease stability cycle, severe tissue ischemia begins to compromise the functionality of the fabrics, causing limitation extreme from the movement, pain at rest, ulcers and gangrene, the what features the ICM. [15] The patient raisin the adopt positions antalgic (flexion knees orhanging limb, massaging the area or forcing walking), progressing with progressive worsening of the flow sanguineous. [16] THE illness arterial peripheral he can to be classified through of parameters clinical proposed put Rutherford [17] and also by Fontaine, [18] as presented node Table 1.

Table 1. Classification of Rutherford and Fontaine to DAP

**Fontaine**		**Rutherford**		
**Internship**	**Frame Clinical**	**Category**	**Frame Clinical**	**Objective Criteria**
**I**	Asymptomatic	0	Asymptomatic	Normal treadmill or reactive hyperemia test
**IIa**	Mild claudication	1	Mild claudication	Completes treadmill exercise; ankle pressure after exercise >50mmHg but at least 20mmHg lower than resting value
**IIb**	Moderate claudication	2	Moderate claudication	Between categories 1 to 3
		3	Severe claudication	Cannot complete standard treadmill exercise, ankle pressure <50mmHg
**III**	Rest pain	4	Rest pain	Resting ankle pressure <40mmHg; ankle or metatarsal pulse volume recording flat or barely pulsatile, tool pressure <30mmHg
		5	Smail tissue loss	Resting ankle pressure <60mmHg; ankle or metatarsal pulse volume recording flat or barely pulsatile, tool pressure <40mmHg
**IV**	Ulcer or gangrene	6	Tissue loss up to the forefoot	Same as category 5

Of that point on, when we observe to the classifications clinics of Fontaine IIb the IV and Rutherford category 3 to 6, clinical treatment no longer promotes an increase in quality of life for the patient, needing of one intervention with revascularization surgical, being this considered the standard treatment for PAD. [7] The surgical revascularization options currently available are: bypasses arterial or bypass (with use from autologous vein or synthetic prostheses), treatments endovascular or percutaneous transluminal arterial angioplasties (with balloons, stents, endoprostheses and atherotomes), in addition to treatments palliatives as sympathectomies and neurotripsis, being what to the end remain just to the amputations minors or greater. [7, 19] Many patients, same in the best conditions of treatment surgical persist with ulcers of difficult healing (Fontaine IV, Rutherford category 5 and 6),

mainly those resulting from minor amputations, which often require healing for second intention and end put chronicize, with high probability of to evolve abruptly to amputation. [20-22]

1.1 Ulcers

The term ulcer refers to the destruction of the epidermis layer, with extension across the skin planes. which can be variable, from limited to the dermis or eventually reaching subcutaneous tissues or even fabrics more deep. [23] It is defined as chronicle one ulcer what no heals after 6 weeks of treatment adequate (terminology more applicable to ulcers of etiology venous). [24] To the ulcers of the lower extremities are very common, with an estimated prevalence of 1 to 2% among adults in USA [25] and its occurrence have an important effect on public health, that consumes resources,

causing frustration for health professionals and patients, leading to a degradation in their quality of life life and days of work lost. [23]

The etiology of the ulcer can be due to several causes: peripheral arterial disease, insufficiencychronic venous, neuropathic diseases (including diabetic), these three being responsible for more than 90% of cases in the USA [25] in addition to those of traumatic origin (including those caused by burns), chronic osteomyelitis, sickle cell anemia, vasculitis, skin tumors (basal cell and squamous cell), chronic infectious diseases (leprosy, tuberculosis, leishmaniasis), lymphedema, pressure and radiotherapy. [26] In approximately 3.5% of the sick, the cause from the ulcer no and identified. [27] It is highlighted yet, the importance of correct diagnosis, considering that treatments differ and that the introduction of wrong therapy will entail damages to the patient. [28]

Treatment of ulcers generally involves systemic and local aspects, focusing on cleaning and covering the wound, until the body can restore the integrity of the skin and control the basic conditions to provide a better quality of life. [29] The advancement of knowledge in this area has enabled the development of a wide variety of products that can be used in therapy of ulcers. It is worth noting that the treatment of these patients must be multidisciplinary, aimed not only at ulcer, but Yes individual as one all, then the success and directly connected the changes in habits oflife that exposes him the risks. [29]

1.2 Ulcers in Illness Peripheral Arterial and Ischemia criticism

Arterial ulcers result from inadequate tissue perfusion, due to complete or partial blockage. partial arterial supply. They are found mainly in areas of bony protuberance such as malleoli and phalanges because in these more distal tissues of the lower limbs the arteries can be unique and, therefore, angiopathy can lead to permanent arterial insufficiency, not presenting sufficient circulation collateral to the your supply. [27, 30]

To the ulcers arterial present dimensions and depth variable (generally deep affecting muscles and tendons), surrounded by pale skin, with a small amount of exudate, put times secretion seropurulent, discreet edema local, necrosis tissue, skin cold and atrophic, odor foul-smelling, difficult to heal, extremely painful, with atherosclerosis being the underlying disease in most of the cases. [25]

To validate the diagnosis, it is noted: history of intermittent claudication, trophic lesions of the skin and change from the tissue perfusion peripheral, corroborated for the absence of wrists, index ankle brachial (ABI) less than 0.9, in addition to other complementary imaging tests such as arteriography, ultrasound vascular with Doppler, angiotomography between others, demonstrating changes from the circulation arterial.[7]

It should be to take in consideration what, the ulcer in one patient carrier of DAP indicates one much greater tissue involvement than the visible wound, as it generally occurs in more advanced stageslate onset of the disease as a result of the severe ischemic condition of the limb. [14] In addition, there is the fact that what there is one chance reduced of resolution of these pictures without some intervention surgical what restore blood supply to these tissues, which is not always possible, and with that, they occur high rates of limb amputation. [14] Arterial revascularization of the affected limb is a condition mandatory in that internship from the illness, being what us cases us which that no and possible, it is put clinical impossibility of the patient, either due to the absence of an arterial drainage tree or due to failure therapeutic, these may progress to amputation of the affected limb. [14] At this stage where nothing can be done do in favor of revascularization and the patient does not accept amputation, his quality of life is similar with that of terminal cancer patients. [31] Amputation entails a productive decline for patients, worsening quality of life and high costs for

health systems. [7] Added to this is the fact that amputation bigger of the extremities inferior, in these patients, incident one risk of mortality surgicalwhich can reach up to 50% in some series, corroborating the need to try to heal these ulcers due to methods unorthodox. [7]

1.3 Mechanisms of healing and chronification of the wounds

After a superficial wound breaks out, a sequence of events takes place to eliminate the debris from devitalized tissue. Therefore, for the subsequent tissue repair process, it will be necessary to have activation of various inflammatory cells, which are induced by cytokines and exposure to molecules of the headquarters extracellular. [32] One demand extra of nutrients it will be necessary node local from the wound with the purposeof supply the metabolism accelerated provoked for the tissue in repair. Those processes occur simultaneously and they are generally divided in three phases main to the healing of wounds: inflammatory, proliferative and remodeler [32]

The inflammatory phase of wound healing begins soon after hemostasis is achieved, and the The primary objective of this phase is to cleanse pathogens as well as foreign material from the wound and restrict the damage to a localized area. [33] Vascular permeability increases through the induction of vasodilation, allowing neutrophils and monocytes to move to the wound site. A complex sequence of interaction between cytokines will regulate this phase, culminating in the conversion of monocytes into macrophages, often considered to be the main regulator of this inflammatory phase of wound healing. [33]Macrophages not only phagocytose and digest tissue debris and remaining neutrophils, but also secrete factors of growth and cytokines what promote the proliferation tissue and migration cell phone. [34]

After about 3 days of the initial wound, the proliferative phase is based on the production of fibroblasts and collagen, a fundamental substance that will form the basic support of scar tissue. [34] Meanwhile, the endothelial cells enter a phase of rapid growth and angiogenesis occurs within the tissue granulation, creating a rich vascular network that supplies this very active healing area. [34] After about 2 to 3 weeks, the wound goes into a remodeling or maturation phase in which the type of collagen is the usual (type I, rather than the type III seen in a new wound) and the wound tissue matures, resulting in full *cross-linking* and restoration of a structure close to the normal, the that can to be one long process. [35]

An important consideration in physiological wound healing is oxygen supply and voltage of oxygen node bed from the wound. [36] The healing of wounds requires oxygen to interact with numerous cytokines, supply the actively proliferating cells as well as the neutrophils. [36] It is estimated what one wound requires at least 20 mmHg of oxygen tension to be able to heal, being that tensions minors what 5 mmHg has low probability of healing. [37] Those effects seem to be closely related to each other in low oxygen tension situations, not only will there be more necrotic debris that can facilitate bacterial growth, as well as compromising the effectiveness of the immune system in fighting pathogens. [37] Thus, PAD carriers as well as T2DM carriers,with low tensions of oxygen tissue, he has one factor negative to the evolution of your ulcers.

Node however, when others factors pathological enter in game, as one state of illness underlying, a chronic ulcer may form. This refers to a wound that has somehow deviated of course physiological natural of events previously described and parked in some moment. THE underlying mechanism varies widely, but includes factors that influence the blood supply (disease peripheral vascular disease), immune function (such as immunosuppression or acquired immunodeficiency), diseases metabolic disorders (such as T2DM), medications, or previous local tissue injury (such as radiation therapy). Factors external factors such as sustained pressure, temperature and humidity also play an important role in allow or no what one wound scar. [38]

In conditions that do not heal normally, coordination of the physiological repair process tissue exerted by cellular signaling (TGF-β, PDGF, IGF-1, VEGF, FGF) does not occur in a adequate and the biochemical mechanisms mediated by cytokines are not effective (TNF-α, IL-1, IL16, INF- γ). [39] There is one extensive relationship between factors vascular, immunological and infectious what predispose to appearance of one ulcer chronicle. [35, 40]

Lots of times, the chronicity of the injuries occurs as one response immunological exacerbated and harmful front to the tissue damaged, no eliminating, suppressing or controlling the problem. Duringan inflammatory response that makes an ulcer chronic, the predominant cytokine profile is characteristic of one response type Th2 (increase of IL4, IL5, IL9, IL10, IL13), this and, one response inflammatory unbalanced that combines the innate and cellular immune responses, with the interaction of several factors growth. [38] Furthermore, the derangement in the control of metalloproteases (MMP) and regenerative cells. All the pathophysiological processes involved in each clinical situation are still unknown. justifies greater investigations. [38]

1.4 Dressings or covers

The dressing or covering can be defined as a therapeutic means that consists of cleaning and application of material over a wound for its protection, absorption and drainage, with the aim of improving the conditions of the ulcer bed and assist in its resolution. [41] They may, on some occasions, be the definitive treatment; in others, only an intermediate step towards surgical treatment. [41] There is, in market worldwide, various materials for use in dressings that can be used in different stages of treatment of the ulcers, the to know: sanitation, debridement, decrease from the population bacterial, exudate control, stimulation of granulation and protection for epithelialization. Fan et al, [42] suggest that dressings are classified into: passive dressings; dressings with active ingredients; smart dressings; biological dressings; and composites. Another characteristic of the dressings to be used for the treatment ofchronic ulcers is that they must maintain a moist surface to prevent dehydration and death cell repair tissue. In addition, a moist surface can promote angiogenesis, stimulate formation of granulation tissue and epithelialization, facilitate the removal of necrotic tissue and fibrin; serveas a protective barrier against microorganisms, promote pain reduction, prevent excessive loss of liquids and avoid trauma of fabric of healing. [43]

The main types of dressings include: classic simple dressings (impregnated or not with other components); poorly adherent or non-adherent dressings (such as silicone film, polyamide mesh with silicone, transparent polyurethane film); semipermeable films; hydrogels; hydrocolloids; alginates; collagens; polyurethane foams; polysaccharides; sugar; hydrofibers (carboxymethyl cellulose 100%); acid hyaluronic; enzymes; protease inhibitors; activated carbon; silver; fabric screens (impregnated or not), biological dressings, among others. [44,45] In addition to treating ulcers with dressings, it is still necessary debridements chemicals or mechanics, care places with the ulcer, prophylaxis of infections, antibiotic therapy, therapy antifungal, therapies of compression, decrease of the points of friction or of pressure, between others. [46,47]

Although there is a wide variety of dressings, one type of dressing alone does not meet the requirements. to be applied to all types of ulcers, depending on the progress of healing, these must to be constantly readapted.

1.5 Stem cells mesenchymal

Mesenchymal stem cells (MSCs) are present in greater or lesser quantities in alltissues of the organism, even after the embryonic phase, being more easily obtained in some tissues thatpreserve them in greater quantity and with a lower degree of differentiation, such as those found in umbilical cord blood, [48] bone marrow49 [and] adipose tissue50 [or] even peripheral blood after induction of proliferation medullary with G-CSF. [51]

The International Society for Cellular Therapy has established definition criteria for MSCs, beingthat these must adhere to the plastic of the culture bottle during several passes, must preserve plasticity, be able to differentiate into at least three different tissues (cartilage, tissue bone and tissue adipose), he must express some markers of surface cell phone (CD73, CD90 and

CD105, CD71, CD44, CD106) and no he must express others (c-kit, CD11b, CD14, CD19, CD34, CD45, CD79α, CD31), in addition of to be negatives to the antigens leukocytes humans (HLA-DR). [52, 53]

THE tissue adipose represents one abundant source of CTM, and of easy obtaining surgical and practically does not present any contraindication for its acquisition. The fraction of the vascular stroma of the tissue adipose became the focus of investigations after its multipotent characteristics were proven, with advantages potential of applicability in the engineering of fabrics. [54, 55]

However, the preparation of the cells in laboratory to the culture no and identical. THE majority of the researchers isolate the MSCs obtained from adipose tissue using the methodology described by Rodbel and col, [56] long before the discovery of its potential use in tissue engineering, in which tissues are digested with collagenase and cell fractionation is done by centrifugation and the cells can be expanded in bottles of culture.

Many possible applications for the use of CTM in tissue engineering have been studied, [57] but one discovery fundamental he was the of what to the CTM put yes only he has one important paper immunomodulatorand therefore can act by orchestrating the action of other cells, both inflammatory and mature cells of the surrounding tissues. [58-60] Thus, it is possible to hypothesize that the treatment of arterial ulcers with cells can have a dual benefit, with the differentiation of these cells into new blood vessels such as also in the adjustment of immunological mechanisms to optimize tissue repair, but the mechanisms physiological involved in that process yet are not completely elucidated.

1.6 State current from the therapy cell phone to Illness Arterial Peripheral and Ischemia Criticism

Tateishi-Yuyama et al, [61] in a pioneering study carried out with participants with ischemia lower limb critical illness (CI), infused autologous bone marrow mononuclear cells into the limbs affected, including patients with pain at rest, and obtained significant improvement in the ABI index (>0.09), increase in the arterial tree observed by comparing pre and post procedure angiograms, increased transcutaneous tissue oxygenation (increase of 13mmHg), and improvement of ischemic ulcers extremities in 60% of the participants of treated group.

Kawamura et al, [62] in a study with a series of 92 participants with ICM, injected cells autologous mononuclear cells obtained from peripheral blood by medullary stimulation with G-CSF in the limbs, promoting an increase in temperature and improvement of symptoms. They compared, in the study population, the presence of T2DM, need for hemodialysis and the Fontaine classification for PAD. As a result, observed good evolution of the ulcers of the participants in almost all experimental groups, and no amputation was observed in almost all experimental groups that underwent cell therapy, except in the group of ischemic patients classified as Fontaine IV with T2DM who were on hemodialysis.This same author carried out a broader study with 162 patients in the same conditions and with a period oflonger average observation (26.4 months) and concluded that there is a difference when treating carriers of diseases with different etiologies, in addition to the fact that a worse prognosis was observed for participants where low CD34 positive cell count and patients with coronary ischemia, suggesting the need formore studies in this line of research. [63] (HORIE, 2010, p.461)

In a systematic review study carried out by Jiang et al, [64] it was possible to observe that only 14 controlled clinical and randomized studies could be compiled, with the majority being carried out implant of CTM derivatives of marrow bone or of blood peripheral after stimulation medullary and nonewas performed with MSCs obtained and expanded from adipose tissue. The review can observe that the therapy based in stem cells autologous he was associated to a better healing of ulcers of members inferior (12 comparisons, 290 patients, RR = 3.07) with little heterogeneity between the data. They concluded that autologous stem cell-based therapy is effective and safe for improving wound healingof chronic ulcers of the lower limbs, without any major adverse effects related to this type of treatment.

Recently, many plastic surgeons have studied the potential clinical application of stem cells. stem derivatives of the fabric adipose tissue (ASCs), for the treatment of ulcers, already what these stem cells haveeasy access and has a high potential for cell differentiation, in addition to secreting growth factors that canimprove wound healing processes, promote angiogenesis and increase the supply of local blood. This tendency has been studied *in vitro* and *in vivo* in animal models, but there are few randomized clinical trials without humans, there being no consensus on a clinically viable protocol andthat ensures results reproducible. [65]

Thus, the use of CTM for the treatment of arterial ulcers may be a viable alternative, decreasing lower limb amputation rates with low relative risk to treatment. However,therapies based on bone marrow stimulation to obtain MSCs from bone marrow or peripheral blood yet no represent the scenario ideal to the therapy cell phone, by the low, although existing, risks relative the need for mobilization and expansion of these cells by granulocyte colony-stimulating factor (G-CSF). [66] The collection and use of MSCs derived from adipose tissue is a viable alternative and is considered, by some researchers, such as the of better choice, mainly by your good performance in culture, lows risks related to the procedure surgical involved, ease of obtaining and plasticity cell phone. [67]

2. JUSTIFICATION

Of mode general, the index of amputation primary between you carriers of DAP and ICM varies between 10%the 40%, characterizing one big problem of health public. Of that point in ahead from the illness, the clinical treatment does not promote an improvement in the patient's quality of life, making it necessary to perform of an intervention with surgical revascularization for the affected limb, this being considered the standard treatment for PAD and ICM. Despite the various techniques available for revascularization of a member, this will not always be possible. Many patients do not have clinical conditions for any surgical procedure, some have already exhausted all forms of treatment and others, despite having performed the better surgical treatment, they still persist in a situation of ICM, with ulcers that are difficult to heal, usually traumatic or resulting from minor amputations left to heal for a second time intention. In these cases, the occurrence of amputations bigger increases sharply.

In addition, obviously, to the final degradation of quality of life and the pain caused at this stage of the illness, comparable just to the carriers of Cancer terminal, to the amputations end being the better

alternative clinic available to those sick. To the amputations generate one important impact socioeconomic, with loss from the capacity labor (removal of work), of socialization and, consequently, from the quality of life, constituting itself as one of the more devastating complicationsof chronic degenerative diseases, associated with significant morbidity, disability and mortality, not therefore being an easy option for the patient, their family and the healthcare team involved. This often leads to a commitment by family members or companions to the care of a patient who no longer has autonomy for his/her own care, which makes him/her anotherproblem for the maintenance of structure familiar.

In some situations, both due to non-acceptance by the patient and their family and due to the lack ofof an examination that can predict with certainty the best moment to decide on amputation, or vice versa, amputation is postponed or, on the contrary, amputations are performed at a time when a conduct more conservative. Generally, you sick amputees if make more dependents and there is one general deterioration of health, directly correlated with loss of mobility. As a result, there is a increase in health spending, especially by the SUS, in the case of Brazil, considering care outpatient care, emergency care, hospitalization, use of analgesics, antibiotics, dressings, among others.

Clinical studies with autologous MSCs are still uncommon and could not be found in the literature. scientific use of MSCs originating from adipose tissue, expanded in the laboratory, used in perilesional injections in association with application in the form of a customized stable bicurative, for the treatment of sick with DAP and ICM without possibility of revascularization/revascularization incompleteand carriers ulcers arterial and risk eminent of loss of member, when in the impossibility of revascularization surgical, in all over the world.

This clinical trial was carefully designed to observe the effects of the treatment of arterial ulcers with autologous MSCs from adipose tissue expanded in culture. The the same will be applied with perilesional injection and also applied using a dressing that can be kept in place for 7 days,the end of provide time enough for the migration cell helping in the healing of ulcers arterial in patients with PAD and ICM. Wound healing rates, salvage of members, your possible effect promoter of increase from the perfusion and, put end, improve from the quality of life to those sick. Like this, expected what to the CTM exercise a paper immunomodulator orchestrating the action of other cells, both inflammatory and mature cells from the surrounding tissues, as well as can act towards cell differentiation to promote angiogenesis as well as differentiate into other cell types helping in tissue repair (fibroblasts, keratinocytes and other cells of the dermis and epidermis).

3. OBJECTIVES

3.1 Objectives Primary

To evaluate the safety and efficacy of perilesional injections of mesenchymal stem cells (MSCs) adults autologous expanded *in vitro* obtained of tissue adipose abdominal and diluted in plasma autologous

in association with the application of the same CTM in the form of a stable biocurative, in a single session treatment for patients with peripheral arterial disease (PAD) with critical limb ischemia (CLI), with ulcer arterial in member lower put time bigger what 3 weeks, without possibility of limb revascularization, incomplete revascularization, or revascularization with restenosis or occlusion, classified as Fontaine IV and Rutherford 5 for DAP, comparatively evaluating the occurrence of limb amputations and partial or total healing of the ulcer, compared to the group that will receive conventional dressings with Hydrogel with acids fatty essential (AGE).

3.2 Objectives Secondary

- To verify the effects of treatment with perilesional injections and stable biodressing with adult MSCsautologous on:
    - Perfusion of member and tissue;
    - Quality of life;
    - Pain;
    - Bed from the ulcer;
    - Parameters clinical and physiological
    - Profile inflammatory systemic
    - Assessment histological from the injury
- To assess the level of perception of health of the participants;
- To assess the rate of rescue of member (no occurrence of amputation)

4. DESIGN DO STUDY

4.1. Description of design of study

Study clinical, prospective, comparative, randomized, controlled and open with assessment “blind” to you outcomes - *Prospective randomized open blinded endpoint* (PROBE). [68]

THE study consists of of screening/start (criteria of inclusion and exclusion, randomization) handling of the CTM, application, follow-up of the participant by 120 days (visits 2,3,4,5) and the visit end (6).

4.2. Justification to the design of study

4.3.

AND one rehearsal clinical phase I/II, prospective, randomized, controlled and open with assessment “monoblind” to you outcomes.

It was designed as a phase I/II clinical study because there is no possibility of carrying out a phase I study, since the procedures necessary for its performance in healthy volunteers (with intact skin) are not justifiable from an ethical-professional point of view according to the Declaration of Helsinki. Therefore, a minimum number of participants will be allocated to this study, justifying the results. previous data obtained from scientific literature and strictly following the Good Practices in Cells standards Humans for therapeutic use and clinical research, established by resolutions RDCs No. 214 of 2018 and No. 260 of 2018 - ANVISA. [69, 70]

THE study it will be open, but you outcomes will be analyzed put evaluators independent with judgment of the outcomes in a way “blind”. Unable to elaborate the essay clinical controlled by placebo and double blind, as we could violate ethical issues as we would have to expose the volunteers, very weak, to an unnecessary surgical procedure (removal of a sample of adipose tissue) tothe group placebo, in addition from the application of injections and dressings placebo, what could to harm yet morethis group of patients. We believe that the proposed clinical trial can present the results in a clear, without increasing exposure to the risks caused by a placebo group, which is “blind” to the outcomes - Prospective randomized open blinded endpoint (PROBE).

4.3 Location of Study and ethics

The study will be carried out at the Hospital das Clínicas of the Faculty of Medicine of Botucatu. The CTM will be prepared at the Cell Processing Center (CPC) of the Applied Biotechnology Laboratory of Botucatu Medical School – UNESP. The study is approved under the CEP/CONEP protocol CAAE39873320.3.0000.5411

4.4Calculation sample and treatment statistical of the results

4.4.1 Size of Sample and Recruitment

The sample was estimated considering the number of patients seen annually at the outpatient clinic vascular dressings at the Hospital das Clínicas of the Faculty of Medicine of Botucatu – UNESP, during the period of 20 months with ease considerable and that fit into the criteria of eligibility of the study. THE Recruitment will take place at the Hospital das Clínicas of the Faculty of Medicine of Botucatu - UNESP and in the Unit of Search Clinic from the Faculty of Medicine of Botucatu – UPECLIN- FMB/UNESP.

THE sample calculation took into consideration two independent samples with estimated proportion in the ulcer healing rate of 75% for group 1, which will be treated with the proposed cell therapy in this study and 30% to group 2, what it will be treated with Hydrogel with AGE.

Assuming a test power of 80% and a significance level of 5% for a two-sided test, the sample is defined as 20 participants for group 1 and 20 participants for group 2 (1:1 allocation). Predicting what 5 participants possibly no will receive the treatment proposed after the inclusion, thesewill be replaced, totaling a probable sample of 45 participants in total. This study was designed to be analyzed by intention to treat *(* ITT). Participants will be from both genres, with age between 18 the 90 years.

Categorical variables will be assessed by the mean with Fisher's exact test, continuous variables will be compared with non-parametric methods such as the Mann-Whitney test or U test, comparisons paired analyses will be evaluated with the Wilcoxon test, multivariate analyses will be evaluated with analysis of regression of Cox, analysis clinics of far away deadline will be compiled in curves Kaplan-Meir.

4.4.2 Groups proposed in the clinical trialGroups of study

The study will have two groups of participants that will be randomized (as will be better explained node item Randomization):

Group 1 – Treatment with Therapy Cell phone. It will be compound put 20 participants (n=20) what will receive treatment with expanded MSCs. The patient will attend for a procedure of debridement surgical from the ulcer to what this stay in your better condition (Visit 1). In sequence,the patient will receive the application of CTM by perilesional injections and a biodressing produced by the team of the study also containing the same cells (single session - the dressing containing CTM will be in contact with the ulcer for 7 days). After the biological dressing is removed, you will receive local ulcer care with topical hydrogel dressing (Curatec Hydrogel with AGE), dry gauze and crepe bandage with minimum change once a day, according to it will be better detailed in the items the follow.

Group 2 – Control Group. It will consist of 20 participants (n=20) who will receive care ulcer sites. The patient will attend for a surgical debridement procedure of theulcer so that it is in its best condition (Visit 1). Next, the patient will receive the application conventional dressing with Hydrogel (Curatec Hydrogel with AGE), dry gauze and crepe bandage. It will be oriented the carry out the replacement to the minimum one time to the day, according to it will be better detailed us items the follow.

4.5 Criteria of Eligibility

Eligibility criteria were defined to select participants for whom treatmentproposed for considered suitable

4.5.1 Criteria of Inclusion

Table 2. Criteria of inclusion

**Description of criterion of inclusion**	**Description of method of calibration of criterion of inclusion**
Term of consent free and enlightened signed and dated	Agree with all the terms of the research
Both you sexes, of any ethnic origin, with age between 18 and 90 years	To be greater than age
Absence of pulses distal in the leg (tibial previous, pedious and tibial later) and presence or absence of pulse popliteal	Assessment from the absence of pulses for theclinical evaluation
Peripheral Arterial Disease	Proven by arteriography or by ultrasound vascular with Doppler;
To have ulcer (s) node foot or in the leg (third distal) of nodeminimum 1cm ^{2} of area and maximum of 3 ulcers totaling until 20cm ^{2} of area in member lower.	Clinical evaluation of the lower limbaffected
To have received previously treatment with dressings conventional to ulcer characterizing what no there was improvement in a minimum period of 3 weeks (antibiotic therapy – if necessary – local care) as debridements mechanic, surgical or chemicals – case necessary, dressings);	Assessment clinic of member lower affected in for the less two queries clinics specialized

DAP with IC classified as Fontaine IV	Present clinically arterial ulcer
And Rutherford 5;	proven in member lower affected
To have index ankle arm (ITB) <0.9 in the arteries infragenicular (tibial previous, pediosa, tibial later and fibular) or ABI>1.3 in one or more arteries infragenicular when carrier of DMT2 chronic (disease duration > 5 years) not subjected to treatment of revascularization or submitted to the treatment with partial revascularization or failure of the treatment (stenosis or occlusion) us last 12 months.	Realization of ITB, assessment clinic and of medical record doctor.
Impossibility of revascularization arterial of affected limb (no visible arteries) infragenicular to approach surgical) and or incomplete revascularization of this limb (submitted endovascular and/or surgical treatment that was not able of restore pulses infragenicular) and defined for the doctor what the better treatment available from a vascular surgical point of view has already been accomplished and no if got success in promote the healing from the ulcer;	Exam radiological (ultrasound) with Doppler, Arteriography or Angiotomography)accomplished in until 12 months from the assessment clinical proving the impossibility of new surgical approaches to the limbaffected.
Drug treatment comorbidities;	Finding clinic of treatment standardwith antiplatelet agents platelets in use for the patient node moment from the inclusion andtreatment of the other comorbidities.
Availability to attend consultations medical;	To respond what it presents interest in attend medical appointments that will be scheduled during the search.

After the inclusion of sick node study, independently of group allocated, these will be referred for collection of laboratory tests: blood glucose, glycated hemoglobin, total cholesterol, HDL, LDL, triglycerides, complete blood count, uric acid, urea, creatinine, CRP, sodium and potassium, blood profile cytokines immunomodulatory.

4.5.2 Criteria of Exclusion

Table 3. Criteria of exclusion

Description of criterion of exclusion	Description of method of calibration of criterion of exclusion
Be pregnant or node puerperium	Confirmation diagnostic of pregnancy or be in puerperium.
Have an ulcer healed during the period of screening	Confirmation to the exam clinical what theparticipant he had to the ulcers healed during the screening.

Have signs of systemic infection or active infection in the ulcer arterial or infection of prosthetics surgical (bypass, pins, screws). They may to be again eligible if submitted the treatment with antibiotics with success and removal of focus infectious.	Change in level laboratory (leukogram or PCR) revealing signs of infection activity systemic or local. Assessment clinic of the features from the ulcer arterial presenting signs of infection active (blush, purulent secretion, heat local and crackling).
To have ulcer with tissue devitalized (necrosis). They may to be again eligible if submitted the successful surgical debridement or amputations minors (amputation of toes or limited to the forefoot).	Assessment clinic from the ulcer arterial what prove necrosis tissue.
To have neoplasia and/or be in treatment chemotherapy or radiotherapy or in remission there is less than 6 months;	Assessment of historical clinical confirming what if treats of patient oncological, in treatment or remission there is less of 6 months.
Be in use of colchicine or immunomodulators;	History clinical of use of colchicine or immunomodulators.
To have illnesses infectious-contagious as Virus from the Immunodeficiency Human (HIV/AIDS), Hepatitis viral type B and W, Virus Lymphotropic from the Cell T Human (HTLV); due to the risk of contagion during to the manipulations of material biological in laboratory.	Laboratory tests for confirmation diagnostic.
To have accomplished amputation in level of leg or of thigh node member what if intends to study.	Exam clinical proving amputation bigger of member what if intends treat.
To have COVID-19, diagnosed in time lower there is 4 weeks	Diagnosis laboratory by qRT-PCR. After the negation of exam, you patients they can become eligible for the study.

Participants excluded during the screening period, which corresponds to the time between the assessment initial and the time of return to check the screening exams and reevaluate the arterial ulcer, before receiving treatment with advanced cell therapy, they may be replaced by another in the same position randomization. Also at this stage, participants may be observed for one month to improve their clinical conditions and initiate the actual study procedures. After collecting adipose tissue for obtaining expanded autologous MSCs, reasons for worsening health and injury may be factors impediments for the participant to receive the treatment proposed in this study, and it is possible that the same wait up to one month to receive study treatment. If the participant does not receive treatment proposed in the study, this will be excluded and a new participant will be elected to occupy the position of that excluded participant, as the study will only be valid from the moment of effective treatment of the participant with advanced cell therapy, and only in these cases is it possible to evaluate by intention to treat (*Intention to treat* – ITT).

4.6 Randomization

You participants will be divided in 2 groups randomized 1:1, randomly. This form, will receive a sequential inclusion number from an electronically drawn number table and will receive the treatment proposed.

Participants allocated to Group 1 - Cell Therapy - will receive treatment with the therapy cell phone proposal in this rehearsal clinical (Injections of CTM perilesional in association with biocurative also containing CTM) in one single session of treatment.

Participants allocated to Group 2 - Control - will receive treatment with daily dressing changes and will follow the same agenda of visits of the first group.

Randomization will be carried out by a technician, using program of construction of tables random number tables available at http://stattrek.com/Tables/Random.aspx . 20 positions will be drawn, with number 1 being set for Group 1 – cell therapy and number 2 for Group 2 – form control random and consecutive. THE envelope containing the randomization will be of possession of one professional independent, so that study team members will not know the allocation order until the moment from the collect of tissue adipose.

4.6.1 Methodology and guarantee of quality of results node study open

It is about of one study clinical, prospective, unicentric, controlled, randomized, open with blinded evaluator for outcomes (PROBE – *prospective, randomized, open, blinded endpoint* – study prospective, randomized, open, with result blind). [66]

You evaluators will be members external, no belonging the team of execution of study and will electronically receive images of ulcers identified only with the participant number and visit and an electronic form with the variables already described for ulcer evaluation. After the end of the study, you data will be forwarded the one statistical independent to analysis.

4.7 Procedures of study clinical us groups proposed

4.7.1Collection of Adipose Tissue to obtain autologous mesenchymal stem cells for use inGroup 1

After inclusion and randomization of the participant to group 1, he/she will return within 7 daysfor checking laboratory tests (see more details in item 4.16). A surgery will be scheduled outpatient, with local anesthesia, for the collection of autologous abdominal fat (adipose tissue sample weighing 5g (10g) as soon as possible possible (estimate) of until 7 days).

Outpatient surgical collection will follow all surgical procedure technique standards: 1. asepsis; 2. placement of sterile fields; 3. local anesthesia with 2% Lidocaine without vasoconstrictor exceeding the maximum dose of 4.5 mg/kg, in the right or left lower lateral abdominal region; 4. will proceed one elliptical incision in spindle oriented to to the lines of strength from the skin abdominal with approximately 3cm long and 1.5cm wide, deepening to the limit with the fascia muscle, without the use of electrical cauterization. Hemostasis will be achieved with electrical cauterization if if necessary, the incision will be sutured with separate stitches with 4-0 Vicryl for the subcutaneous, the skin will be sutured with simple 4-0 NYLON stitches. A sterile dressing with cotton gauze and Micropore® will be applied on the local.

The tissue obtained will be placed in a sterile 50ml conical plastic tube containing 30ml of medium. transport HEPES and this it will be transported in condition refrigerated between 4ºC and 8ºC to the CPC of Blood center of Hospital of Clinics from the Faculty of Medicine of Botucatu (FMB) - UNESP.

4.7.2 Processing from the sample of Tissue Adipose

THE sample of tissue adipose it will be processed node CPC being coded with one sequence numerical for identification and storage of human material, with electronic registration, in order to provide the secure supply of CTM autologous to the participant.

The adipose tissue will be manipulated in a laminar flow cabin in an aseptic manner. Initially, it will bewashed with 0.9% saline and placed in a new 50ml sterile plastic conical vial of medium HEPES plus ciprofloxacin 10% (32 µg/mL) and amphotericin B 50µg/ml to prevent proliferation of bacteria and fungi, kept in this solution in a refrigerator at 4°C for 24 hours for quarantine purposes. decontamination.

After this period, the adipose tissue will undergo enzymatic dissociation, where the tissue will be fragmented in order to increase the surface area of action of the enzyme and 4mg collagenase will be added type I in 2ml of Xeno free additive (MesenCult®, Stem Cell ™) for the proportion of 1g of adipose tissue. This solution containing adipose tissue will be stored in an oven for 15 hours so that the enzyme dissolve the collagen matrix and release the cells. At this point, the same volume of Xeno free additive (MesenCult®, Stem Cell ™) will be added to inactivate the enzymatic action of collagenase type I. This solution will be centrifuged at 1200 rpm for 10 minutes. The supernatant will be discarded and the *The pellet* obtained will be subjected to 3 washes with resuspension in HEPES medium to eliminate debris cellular, liquefied fat, extracellular matrix and blood cells. The *pellet* obtained at the end of this washing process will be suspended in 1ml of Xeno free culture medium with additives (MesenCult Stem Cell®)for seeding. An aliquot of 30uL will be collected for cell counting in a Neubauer chamber to verification of cell viability and quantification of the number of total viable cells (control protocol initial quality). Subsequently, the viable cells obtained will be plated in culture flasks 25cm2 in the approximate proportion of 2,000 to 3,000 CTM/cm2 and then 4mL of medium will be added · Xeno culture free additive (MesenCult®, Stem Cell ™) per vial to follow up to culture. All to the information obtained during the processing cell phone will be registered electronically.

4.7.3 Procedures of Expansion Cell phone

Culture flasks 25cm^2 obtained after sample processing will be placed in an oven humid at 37ºC, with 5% CO_2 (Thermo Class 100®) until a confluence of more than 80% of thearea of the bottom of the plate, through direct monitoring by inverted microscopy. The culture medium will be changed every 48 hours. After reaching 80% confluence of the plate area, the procedure will be performed increasing the number of bottles, so as not to inhibit cell growth (competition for nutrients and space) this moment is called passage. The passage procedure will be done with the detachment of the cells in the culture flask by adding 0.1 mL of trypsin (Stem Cell™) for each cm2 of the culture flask area. bottle, maintained put 5 minutes in stove the 37ºC. After that time, it will be added equal volume of quite

of Xeno free culture additive (MesenCult®, Stem Cell ™) for trypsin neutralization. This volume containing the cells will be centrifuged and resuspended twice and the cells obtained will be counted and replated into 75cm2 flasks (methods described in detail in the previous section). The passages will be repeated until the number of cells required for the application procedures and for the quality controls (see more details below in item 4.11 cell quality control). It is expected thatthe number of cells it is reached in 3 or 4 passages (for the less $1X10^{7}$ CTM, with time estimated in 21 to 28 days from initial processing). All information obtained from cell processing will be electronically recorded.

4.7.4 Control of quality cell phone

The preparation of the product to be used will follow ANVISA standards for material safety. biological, specifically resolutions RDC 214/2018 and RDC 260/2018. [67, 68] All cell samples will be electronically catalogued in a secure database and will be used exclusively for participant of the study (autologous), using code of bars in the tubes for identification.

4.7.5 Control microbiological from the culture cell phone

For cellular quality control, with regard to fungal and bacterial infection, a sample of 1mL of culture medium from the flasks containing cells in each passage will be stored in a type flask Bactec® and sent to the microbiology laboratory for analysis. In case of contamination, the vial ofculture will be discarded. For analysis of the presence of mycoplasma, a sample of $1x10^{6}$ CTM of first ticket will be sent to the Molecular Biology laboratory. In case of confirmation of infection by mycoplasma the laboratory should suspend to the activities and all to the cultures will be discarded and the laboratory it will be submitted to the procedures of environmental decontamination.

All cultures will be monitored three times a week by direct inverted optical microscopy, to verification of cell confluence and prevention of eventual contaminations with fungi and bacteria.

4.7.6Control of quality of the stem cell

The analysis for immunophenotypic characterization of MSCs will be performed by flow cytometry, so that the cells obtained will be better characterized as CTM. For this, analyses will be carried out a minimum panel of 3 positive markers (among these: anti-CD44, anti-CD73, anti-CD90 or anti- CD105) and 3 negative markers (among these: anti-CD11b, anti-CD14, anti-CD19, anti-CD31, anti-CD34,anti-CD41, anti-CD45 or anti-CD79α). A sample of $1x10^{6}$ third-pass MSCs, in the form of *pellet*, will be sent to the flow cytometry laboratory, which must be processed within a maximum periodof 2 hours.

Cell viability analysis will be performed by flow cytometry by determining the index of apoptosis/necrosis using annexin V, propidium iodide and Caspase-3 kit. For this, a sample of $1x10^{6}$ third-pass CTM, in *pellet form,* will be sent to the flow cytometry laboratory, that should be processed in one term maximum of 2 hours.

Cell differentiation analysis will be performed as an additional test to ensure that MSCs they are capable of if differentiate in others types cell phones. One sample of $3x10^{6}$ cells of third ticket

^{25cm2} culture flasks and will be subjected to the differentiation protocol in tri- lineage (3 different cell lines - adipocytes, chondrocytes and osteocytes) through the use of the kit appropriate to differentiation cell phone *StemPro adipogenesis, chondrogenesis, and osteogenesis kits* , Invitrogen®, according to guidelines of the *International Society for Cellular Therapy* (ISCT).

4.7.7Assessment of expression genetic put RT-qPCR

One sample of 1x106 CTM of third ticket it will be sent to the Laboratory of Biology Molecular to carry out genetic testing through of the reaction technique in chain from the polymerase in timereal after transcription reverse (RT-qPCR) to one panel of to the less 20 genes, including genes relatedcell undifferentiation (Nanog, c-Myc, Oct4, Sox2 and Klf4), gene related to lineage characteristics adipogenic (PPARG), genes suppressors tumoral (APC, WT-1, RB, p53, NF-1 and DCC), genes relatedgrowth factor receptors (ERBa, ERBb and p28 ^{sis}), genes related to regulatory proteins transcription (p110myc, p55fos, p48myc) and identification of some oncogenes that may be present in disarray, to prevention of neoplasms (C-myc, N-myc, L-myc, C-erb, Neu/erb-B2).

The guidelines of *Minimum* information for Publication of Quantitative Real-Time will be followed. PCR Experiment (MIQE). [71] Initially the acid ribonucleic (RNA) total of CTM will be extracted using Trizol (Life Technologies, USA), according to the manufacturer's instructions. RNA samples will betreated with TURBO DNase (Ambion, USA) to remove genomic DNA contamination. In then, the RNA it will be quantified put spectrophotometry using the equipment NanoDrop (ThermoFisher, USA). The quality of the extracted RNA will be determined by the RNA integrity number (RNA Integrity Number, RIN) from the analysis of ribosomal RNAs using the 2100 system Bioanalyzer (Agilent, USA). [72] After assessing the quality of the RNA, reverse transcription will be performed. for mRNA. For this purpose, the High Capacity RNA-to-cDNA Master Mix kit (Life Technologies, USA), following the manufacturer's instructions for a total reaction volume of 20μL. The reaction conditions thermocycling will be the same as those indicated by the manufacturer. Quantitative PCR will then be performed onTime Real (qPCR). Will be made using SYBRgree Universal PCR Master Mix, Node AmpErase® UNG twice (Life Technologies, USA) for a total reaction volume of 20μL, according to recommendations of manufacturer. Will be used primers specific to you transcripts (mRNA)of the target genes. Primer Blast will be used to construct the primers, [72] recommending flanking regions between exons. The quantification relative from the expression genetic it will be carried out using the method of Cq comparative. [73]

Table 4. Compliance standard for mesenchymal stem cells

Test	Result	Accordance
Contamination by inverted microscopy.	Absent	According
Microbiology	Aerobic: Negative<br>Anaerobic: Negative<br>Fungi: Negative<br>Mycoplasma: Negative	According
Cell Differentiation	Adipogenic: Positive for Oil Red<br>Chondrogenic: Positive for Toluidine Blue<br>Osteogenic: Positive for Alizarin Red	According
Imunofenotipagem	CD44, CD73, CD90, CD105: Positive >90%<br>CD11b, CD14, CD19, CD31, CD 34, CD45, CD79α: Negative <5%	According

Annexin V / Propidium Iodide / Caspase-3	Apoptosis/Necrosis Index < 5%	According
Molecular Biology	Identification of expression of genes of undifferentiation Functionality: positive<br><br>Conforms to Identification of expression of tumor genes: negative	According

4.8 Justification for amount of cells (Dose)

Based on the few studies found in the scientific literature in which cell therapy was used for the treatment of PAD with IC, with no prospects for treatment with revascularization, was observed great variability methodological in the form of application and "dose". To the main variables found were in relation to the amount of CTM that should be applied. It is generally used as calculation basis the surface area of the ulcer. In studies that used intraarterial injections or periarterial, the stones were based on the extension of the vessels. In the studies where the injection was made intramuscular took into consideration the volume muscular. Put other side, the number of cells cultivatedand the quality assurance of CTM was dependent on several factors such as: culture time, number of passages in culture, the age of the participant, the site of collection (peripheral blood or bone marrow) between others.

So, in order to standardize the method that will be used, enabling its repetition,This study will use a number of cells proportional to the area of the ulcer (dose). In this way, each puncture will apply 0.1ml of solution (autologous plasma) containing 10,000 CTM ($1X10^{4}$ CTM/0.1ml of solution). THE injection containing the solution (plasma + CTM) it will be applied node tissue subcutaneous from the edge from the injury, the each one cm, of form the surround the ulcer.

To increase the chances of epithelialization, MSCs will also be applied topically. To this, a biological and bioabsorbable dressing, which will be produced for this study, will add 10,000 CTM for each cm2 ^{of} ulcer (1X104 ^{CTM} /cm2 $^{)}$ which will be secondarily covered with gauze and crepe bandage (see more details the follow in Dressing with CTM).

4.9 Preparation, storage and procedures to dispensation of product

Once the cell count is obtained in culture so that clinical use can be made in participant, the CPC team will contact the study team to summon the participant and carry out thetreatment proposed (Group 1 – Therapy cell phone) in one period maximum of 7 days.

If it is impossible to attend or there is an active infection in the ulcer on the day of attendance, the CTM will be cryopreserved in liquid nitrogen at 196 ^{o}C on the CPC premises. The participant of the study may still receive therapy with their cells if they are eligible within a maximum period of 1 month.

One sample of $1X10^{6}$ CTM of each participant of Group 1 will be cryopreserved in freezer the80 ^{o}C in the dependencies of CPC with record cell phone to possible analysis future, if necessary and as guarantee stock in case of contamination of the culture in the amplification stage. These cells will be stored during the period of study and then will be discarded in trash appropriate for incineration.

For group 1, which will receive treatment with advanced cell therapy, with 20 participants, the The test product will be presented in two forms that will be applied together: in the form of a biocurative stable, this will consist of a thin sheet (2mm thick) of Fibracol™ Plus (90% collagen + Calcium Alginate 10%) that will be soaked in a solution

containing fresh autologous plasma and MSCs from the participant, in a personalized way, where the dressing will be produced in a way that promotes coverage of the ulcer area, with a proportion of $1x10^{4}$ CTM being made available for each cm^{2} of dressing. During dressing production, the plasma solution containing MSCs will be induced to "gel" (gain consistency so as not to run) with the addition of Thrombin 800–1200UI/mL in a proportion of 1/10mL of the plasma solution to be applied + Calcium Gluconate 10% in the same proportion as Thrombin. The The finished dressing will be packaged in Petri dishes and sealed. A sterile secondary packaging of plastic will insulate the product. The product must be transported in a refrigerated Styrofoam box (between 2 and 8°C), sealed and with the identification of participant from the search; in the form of solution to injection perilesional, fresh autologous plasma will be used for dilution of $1X10^{5}$ CTM for each 1mL of the solution and will be available in 1mL syringes with a 27G hypodermic needle. The application will be 0.1mL for each centimeter of edge from the injury. To the syringes containing the solution of plasma fresh and CTM will be produced in the proportion that is estimated to be applied to that participant. The prepared syringes will be packaged in sterile plastic packaging, which will isolate the product. This product must be transported in a refrigerated Styrofoam box (between 2°C and 8°C), sealed and with the identification of the participant of the search, being the same styrofoam what will transport the dressing.

After the product is properly packaged, it must be applied to the participant within a maximum period of 4 hours. The packaging will be transported by the person responsible for distributing this product to the location where it will be applied (center surgical).

Participants in group 2, control, with 20 participants, will receive local ulcer care, and the participant will attend to undergo an ulcer revitalization procedure, to that it is on an equal footing with group 1, which will be carried out on Visit 1. Then, the participant will receive the dressing application conventional with Hydrogel (Curatec Hydrogel with AGE), dry gauze and crepe bandage. You will be instructed to change it at least once a day and follow the same schedule of queries what you participants of group 1.

4.10 Procedure of application of product

The dosage planned for this clinical trial will be a single dose of the product, whichit will be available to use through of two ways of administration: biocurative stable to applicationtopical on the ulcer arterial and solution injectable to application perilesional hypodermic.

The procedure for applying the advanced cell therapy product proposed in this clinical trial will be performed after locoregional (preferred) or general anesthesia, at the discretion of the anesthesiologist who will perform the procedure, taking in consideration to the conditions clinics of participant, one time what the procedure no he can to be accomplished without the due analgesia. THE application of the injections and of bandage will follow to the standards of procedures surgical, taking place the antisepsis, placing of fields sterile, materials surgical of form sterile and opening of the envelopes containing you products the to be applied of form sterile.One cleaning and a debridement initial surgical procedures will be carried out in the wound in a way to make it suitable forto receive the treatment. They will be, then, proceeded to the applications subcutaneous of 0.1mL of product what consists of in the solution of plasma and CTM autologous expanded the each 1 cm from the edge from the ulcer (node subcutaneous from the skin full node perimeter from the injury), of form what approximately $1x10^{4}$ CTM be injected put puncture. After the injections, the topical application of the biocurative will be carried out stable containing to the "Gelified" CTM in the membrane of Fibracol™ on the area from the ulcer arterial. In then, the bandage it will be covered with one

gauze of rayon (identifying the end of bandage primary), after it will be placed the bandage secondary with one thin layer of gauze humidified with serum physiological 0.9% and other layer with gauze dry and, put end, the range crepe. Only the bandage secondary it will be exchanged daily for the participant or your caregivers, according to guidelines what will be given for the team of study. THE participant will receive high like this what you are clinically stable. After 7 days, will return to the revaluation from the ulcer, being carried out the replacement of bandage primary and secondary for the team of study, passing by then, to the phase where will perform just dressings with Hydrogel with AGE (the do of absorption of the CTM already he was completed).

4.11 Control of quality of study

4.11.1 Procedures of sampling

- **Obtaining adipose tissue:** the adipose tissue sample will be kept sterile both by the process surgical or by packaging in a sterile conical tube containing HEPES. Transportation will be made in a refrigerated Styrofoam box with controlled temperature between 2 and 8°C, sealed and with the identification of the research participant. The adipose tissue will be handled at the CPC within a period maximum of 2 hours, in a laminar flow hood. It will be washed 3 times with 0.9% saline solution andtube changed, identified to the study participant, now containing HEPES (xenofree - STEMCELL™ Technologies) added of ciprofloxacin 10% (32 µg/mL) and amphotericin B 50µg/ml. It will be kept in this solution in a cold chamber, specific for this purpose, at 4°C for 24 hours to decontamination quarantine.
- **Obtaining CTM for expansion:** After quarantine, the adipose tissue sample will be submitted enzymatic dissociation for 15h. The dissociated solution will be subjected to centrifugation at 600xg for 10 minutes. The supernatant will be discarded and the *pellet* obtained will be subjected to 3 washes with resuspension in HEPES medium (xenofree - STEMCELL™ Technologies) to eliminate the cellular debris, liquefied fat, extracellular matrix and blood cells. The *pellet* obtained at the endof this washing process will be suspended in 1ml of culture medium (MensenCult-S - xenofree - STEMCELL™ Technologies). One aliquot of 30uL will be collected to cell count in Neubauer chamber for checking cell viability and quantifying cell number total viable cells (initial quality control protocol). Cells will be plated in flasksculture of 25cm ^{2} to give follow-up to the culture.

- **Cell expansion control: The** 25cm2 culture flasks obtained after processing the sample will be placed in a humid oven at 37°C, with 5% CO2 (Thermo Class 100®) until observe one confluence superior the 80% from the area of bottom from the plate, through of monitoringdirectly by inverted microscopy. The culture medium will be changed every 48 hours, always in a manner sterile in laminar flow. If changes are observed in the cell expansion flask that suggest slight contamination, the supernatant will be sent for microbiological analysis and the culture vial it will be discarded. In observation of Contamination external of bottle of culture, these will be decontaminated following laboratory protocol and all the bottles of culture of stove will be observed under inverted microscopy to identify internal contamination, with the positives discarded. THE stove it will be off and decontaminated second protocol of laboratory.THE procedure of ticket cell phone it will be monitored according to described below. All to the information obtained of processing cell phone will be registered electronically, including count cell phone and feasibility cell phone. All you bottles always will be identified to the participant.
- **Microbiological control of cell culture (fungi and bacteria):** a 1mL sample of medium of culture of the flasks containing cells in each passage will be stored in a Bac-type flask. tec® and sent to the microbiology laboratory for analysis. In case of contamination, the vialwith culture it will be discarded.
- **Microbiological control of cell culture (mycoplasma):** a sample of $1x10^{6}$ CTM of pri- The first passage will be sent to the Molecular Biology laboratory. In case of confirmation of in- infection by mycoplasma the laboratory must suspend activities and all cultures will be discarded. cards and the laboratory it will be submitted to decontamination procedures environment
- **Quality control of MSCs (immunophenotypic characterization**): A sample of $1x10^{6}$ MSCsof third passage, in form of pellet, will be forwarded to the laboratory of cytometry flow.
- **Quality control of CTM (cell viability):** a sample of $1x10^{6}$ CTM of third ticket, in form of pellet, it will be forwarded to the laboratory of cytometry flow to carry outapoptosis/necrosis index determination test using annexin V kit, propidium iodide and Caspase-3.
- **Quality control of CTM (cell differentiation):** a sample of $3x10^{6}$ cells third passage will be plated in 3 bottles of culture of $25cm^{2}$ and will be submitted to the protocol of differentiation in tri-lineage (3 lineages cell phones different - adipocytes, chondrocytes and osteocytes).
- **Quality control of CTM (Gene expression assessment by RT-qPCR):** a sample of $1x10^{6}$ CTM of third ticket will be sent to the Laboratory of Biology Molecular to perform genetic testing using the real-time polymerase chain reaction technique after reverse transcription (RT-qPCR).

- **Freezing of sample:** a sample of $1x10^{6}$ CTM of each participant in the group that will receive treatment with advanced cell therapy will be cryopreserved in a freezer at -80°C for possible future analyses during the period of study.
- **Cell therapy dose:** will be characterized by automatic counting of 30uL sample of the solution of 1mL containing the stem cells.

_4.11.2_ Requirements to acceptance of batch of materials, reagents and products to diagnosis _in vitro_ used in the manufacturing of product and us processes of control of quality

All culture media, reagents and supplements that will be used in cell culture must have xenofree quality control and sterility guaranteed by the manufacturer, in addition to batch identification manufacturing and origin on the packaging for product traceability; shelf life of more than 6 months, identification of concentration of the your components chemicals and packaging no raped. All you materials used in cell culture will be single-use, discarded after use, acquired in a manner sterile direct of manufacturer, with identification of batch of manufacturing and origin in the packaging to product traceability; shelf life of more than 6 months, identification of its chemical components andunbroken packaging. Diagnostic kits must present identification of manufacturing batch and origin in packaging for product traceability; shelf life of more than 6 months, identification of its components chemicals and packaging no raped. All you procedures will be carried out put operational procedures standard and will be accordingly with the DRC 214/2018 and DRC 260/2018.

For internal quality control, after opening the culture medium and additive bottles, and/or other products, they will be preserved as described in the manufacturers' user manual (in cold rooms from 2°C to 8°C and/or freezer -20°C and/or freezer -80°C). They will be subjected to tests microbiological whenever changes in turbidity are observed and/or the formation of granules is observed or color changes or other changes suggested by the manufacturer and discarded. All products will be aliquoted per participant and the bottles will be identified, so that no product that may presented environmental contact with cells of one participant can be placed in contact with another participant.

4.11.2 Requirements of security and quality to liberation of product of therapy advancedinvestigational to administration

- **Control of quality**

  The preparation of the advanced cell therapy product of this study will follow ANVISA standards for the safety of biological material, specifically resolutions RDC 214/2018 and RDC 260/2018. Allcell samples will be electronically catalogued in a secure database and will be destined exclusively to the participant of study (autologous), using code of bars us tubes to identification.
- **Control microbiological from the culture cell phone**

  For cellular quality control, with regard to fungal and bacterial infection, a sample of 1mL of quite of culture of the bottles containing cells in each ticket it will be conditioned in bottle type

Bactec® and sent to the microbiology laboratory for analysis. In case of contamination, the vial of culture will be discarded. For analysis of the presence of mycoplasma, a sample of $1x10^{6}$ CTM of first ticket will be sent to the Molecular Biology laboratory. In case of confirmation of infection by mycoplasma the laboratory should suspend to the activities and all to the cultures will be discarded and the laboratory it will be submitted to the procedures of decontamination environmental.All to the cultures will be monitored three times a week by direct inverted optical microscopy, to check confluence cell phone and prevention of eventual contaminations with fungi and bacteria.

- **Control of quality of the stem cell**

The analysis for immunophenotypic characterization of stem cells will be performed by cytometry. flow, so that the cells obtained will be better characterized as stem cells. For this, they will be analyses were performed on a minimum panel of 3 positive markers (among these: anti-CD44, anti-CD73, anti-CD90 or anti-CD105) and 3 negative markers (among these: anti-CD11b, anti-CD14, anti-CD19, anti-CD31, anti-CD34, anti-CD41, anti-CD45 or anti-CD79α). A sample of $1x10^{6}$ third-cell MSCs ticket, in form of pellet, it will be forwarded to the laboratory of cytometry flow, what should to be processed within a maximum period of 2 hours. Cell viability analysis will be performed by cytometry flow through determination of apoptosis/necrosis index using annexin V kit, propidium iodide and Caspase-3. To that, one sample of $1x10^{6}$ CTM of third ticket, in form of pellet, it will be forwarded to the flow cytometry laboratory, which must be processed within a maximum period of 2 hours.Cell differentiation analysis will be performed as an additional test to ensure that the stem cells are capable of differentiating into other cell types A sample of $1x10^{6}$ third passage cells will be plated onto each of the 3 25cm2 culture flasks that will be subjected to the protocol differentiation in tri-lineage (3 lineages cell phones different - adipocytes, chondrocytes and osteocytes)through the use of the appropriate kit for cell differentiation StemPro adipogenesis, chondrogenesis, and osteogenesis kits, Invitrogen. You results positives to the differentiation will serve as control of cell quality. A cellular quality control test will also be carried out using Molecular Biology. A third- pass $1x10^{6}$ CTM sample will be sent to the Molecular Biology Laboratory for carry out analysis using a panel of at least 20 genes in order to guarantee their functionality cellular and its non-tumor quality. Such analyses are important to ensure the minimum standard of quality ity for stem cells, thus being suitable for therapeutic use in humans, according to the guidelines of the International Society for Cellular Therapy (ISCT).

- **Assessment of expression genetic put RT-qPCR:**

One sample of 1x106 CTM of third ticket it will be sent to the Laboratory of Biology Molecular of the same Institution, so that genetic tests can be carried out using the reaction technique in real-time polymerase chain reaction after reverse transcription (RT-qPCR) for a panel of at least 20 genes, including genes related to cell dedifferentiation (Nanog, c-Myc, Oct4, Sox2 and Klf4), gene related to the adipogenic lineage characteristic (PPARG), tumor suppressor genes (APC, WT-1, RB, p53, NF-1 and DCC), genes related to growth factor receptors (ERBa, ERBb and p28sis), gnes related the proteins regulatory of transcription (p110myc, p55fos, p48myc) and of identification of

some oncogenes that may be disarranged, to prevent neoplasia (C-myc, N-myc, L-myc, C-erb, Neu/erb-B2). The guidelines of the *Minimum Information for Publication of Quantitative Real-Time PCR Experiment* (MIQE). Initially the total ribonucleic acid (RNA) of MSCs will be extracted using Trizol (Life Technologies, USA), according to the manufacturer's instructions. The samples RNA samples will be treated with TURBO DNase (Ambion, USA) to remove DNA contamination genomic. In then, the RNA it will be quantified put spectrophotometry using the equipment NanoDrop (ThermoFisher, USA). THE quality of RNA extracted it will be determined for the number from the RNA integrity (RNA Integrity Number, RIN) from the analysis of ribosomal RNAs using thesystem 2100 Bioanalyzer (Agilent, USA). After the assessment from the quality of RNA it will be carried out the reverse transcription to capture mRNAs. For this, the High Capacity RNA-to-cDNA kit will be used Master Mix (Life Technologies, USA), following the manufacturer's instructions for a total volume of 20μL reaction. The thermocycling conditions will be the same as those indicated by the manufacturer. The following will be Real-Time Quantitative PCR (qPCR) was performed. They will be made using SYBRgree Universal PCR Master Mix, No AmpErase® UNG twice (Life Technologies, USA) to a total volume of reaction of 20μL, according to recommendations of manufacturer. Will be used primers specific to you transcripts (mRNA) of the target genes. Primer Blast will be used to construct the primers (YE, 2012, p.134), advocating flanking regions between exons. The relative quantification of gene expression will be carried out using the method of Comparative Cq.

4.11.3 Characterization of the active component, including, where applicable, its identity, quantity, purity, feasibility, power, stability genetics and sterility, should describe the methodologiesanalytics maids

The active component of this project's advanced cell therapy product is stem cells. autologous mesenchymal stem cells (MSCs) originating from atypical tissue and which will be expanded in the laboratory. CTM will be made available to the research participant in two presentations for use in a single session of treatment: solution injectable and biocurative stable. According to presented node control of product quality, the CTM will be characterized as to their purity, being necessary that they present thecharacteristics determined by the International Society for Cellular Therapy (ISCT) guidelines. All identity, purity, viability and potency characteristics are described in Table 1 and in item I - SAMPLING PROCEDURES. Regarding the number of cells (dose), samples will be obtained from abdominal adipose tissue (5 to 10 grams) which should provide an initial sample of approximately 2x103 to 3 x103 ^{cells} and after expansion, it is intended to reach at least 2x106 $^{CTM\ for}$ use for the patient (with quality control and back-up samples already reserved). These will be counted automatically in *cell counter* and confirmed by manual counting in Neubauer chamber. It will be considered a viable sample when the number of non-viable cells does not exceed 10% of the total number of cells. The CTM will be aliquoted in a personalized way, so that $1x10^{5}$ CTM are delivered per 1mL of plasma solution to be applied to the edges of the ulcer in a volume of 0.1mL for each centimeter ofedge. For a stable dressing, it is intended to soak the Fibracol with the same plasma solution containing cells in the proportion of $1x10^{4}$ CTM per cm^{2} of ulcer area to be covered. Thus, it is believed that the supply of CTM must to be enough to promote the process of intended tissue repair.

4.12 Reactions or risks relative to the treatment

Risks relative to the use of CTM autologous yet no they were described and to this study they areconsidered non-existent.

Risks relative to the treatment they are considered you related to the procedure anesthetic surgical: surgical site infections, surgical wound dehiscence, worsening of pain in the treatment area, bleeding, gangrene, cellulite.

4.13 Follow-up, Reviews, Procedures and Schedule of Visits

4.13.1 Reviews Clinics:

- Data demographics: includes sex, date of birth, ethnicity, activity physical and level of education;
- History doctor: illnesses of basis with date of diagnosis;
- Exam physical: device vascular peripheral – inspection (features from the skin and injuries trophic) palpation of wrists, auscultation of blows;
- Signs vital: include frequency cardiac, pressure arterial and temperature;
- Anthropometry: includes height (only in the screening) and weight (all to the visits);
- Registration of medications concomitant: must to be registered all you medicines, vitamins,herbal medicines, of use oral, topics, inhalers, intranasal and injectables, with dosage, dose, date of start of use and indication therapy. In each visit, the participant he must to be questioned if startedor discontinued some treatment. THE date of discontinuation also it must be registered;
- Assessment of events adverse: includes assessment of events local adverse effects (ulcer and skin periulcer)and systemic (exams laboratory and symptom clinical) with date of start and end of the symptoms, intensity, if and serious or not, causality in treatment relationship of the study (Yes or no);
- According to the International Conference on Harmonization (ICH), an adverse event (serious or no) is any undesirable clinical occurrence in a clinical research participant who receives or uses a product or treatment and does not necessarily have a causal relationship with them. Thus, an adverse event can be any unfavorable and unintended signal (including a abnormal laboratory finding, symptom or disease temporally associated with the use of a product in investigation, considered related or not to it). Preexisting conditions that suffer worsening during one study must to be reported as events adverse;
  THE event becomes serious when:
- Result in death
- Threaten the life
- Result in incapacity or deficiency permanent, significant or to generate inability ofcarry out functions normal from the life.
- Cause abnormality congenital
- AND one event clinically significant according to assessment of researcher
- Assessment - application of questionnaire of quality of life (SF-36) (Annex I);
- Application from the scale of pain (Scale Visual Analog – EVE) (Annex II);
- Application from the scale of perception of health (Scale EQ-5D) (Annex III);
- Assessment of laboratory parameters: blood glucose, glycated hemoglobin, total cholesterol, HDL, LDL, triglycerides, complete blood count, uric acid, urea, creatinine, CRP, sodium and potassium. If there is result outside the normal range, assess whether there is clinical relevance (adverse event) or not there is relevance clinic (no considered event adverse);

- Assessment of the ankle-brachial index (ABI) of the affected limb: the participant must be in decubitus dorsal horizontal and in rest put, for the less, five minutes. You cuffs of sphygmomanometers must be positioned comfortably, adjusted to the arms, in the same height, above the ulnar malleolus with the " *cuff* " directed towards the path of the brachial artery of each side. Afterwards, the arm with the highest systolic blood pressure (SBP) is chosen to compare it with the MMII. When you values of PAS of the MMSS they are identical is elected the arm right (BD). If there isdifference in values, choose the arm with the highest PAS value. Brachial SBP will be determined in thefish node local from the artery radial with ultrasound Doppler portable of 10 mHz. With the same equipment the ankle SBP will be determined at the dorsalis pedis artery. To determine the valuefrom the ABI, divide the ankle systolic blood pressure (highest value) by the blood pressure systolic of the arm (bigger value);
- Assessment of arteriography carried out previously (case the participant you have done this exam) describing in detail the existing arteries and their conditions in the arteriographic examination (description to be performed by a vascular surgeon). It will be considered valid for the study if carried out until 6 months from the date from the screening;
- Evaluation of vascular Doppler ultrasound. A vascular Doppler ultrasound will be performed with a Logic S7 XD Clear ultrasound equipment with linear transducer 9MHz before any intervention on the participant. The participant must appear without any preparation prior. You will lie horizontally on a stretcher and a certified vascular surgeon will performan exam detailed of the arterial circulation of the member affected, identifying the permeability, occlusions of arteries, characteristics of waves of flow, velocities of flow and stenosis;
- Assessment from the skin from the leg and of foot put Thermography. One camera thermographic professional TermoCam T530-SC at a distance of 40cm from the participant, making at least 4 acquisitions of image (anterior, posterior, lateral and medial). The participant must appear without any preparation prior. You will lie horizontally on a stretcher and a trained healthcare professional with the device will do to the photographs;
- Assessment from the perfusion from the skin with Capnography. One capnograph IntelliVue TcG10® PHILLIPS it will beinstalled on the participant's lower limb, applying the sensor to a specific point on the basis of hallux and one point node ankle back of malleolus medial. THE participant he must attendwithout any prior preparation. You will lie horizontally on a stretcher and a health professional health trained with the device will do the readings of voltage of The $_2$ and CO_2;

4.13.2 Assessment from the ulcer:

The ulcer assessment will be performed using the *Pressure Ulcer Scale* for Healing instrument, as below:

- Assessment of width and length (measures in cm);
- Assessment from the amount of exudate (absent, small, moderate, big);
- Assessment of tissue type (closed wound, epithelial tissue, granulation tissue, slough, tissue necrotic);

In addition of these, others parameters will be evaluated:

- Measurement of the area (cm2 $^{)}$ by photographic analysis: the measurement of the size of the ulcer area will occur by photograph that will be obtained by digital camera at a distance of 30 cm from the lesion identified by label with initials, participant number, ulcer number, visit and date. Measurement of the area in photography it will be carried out for the software ImageJ™;
- Characterization of the edges (flat, little deep, very deep, hyperkeratotic);

- Signs of infection (absent, secretion purulent clear, secretion purulent yellowish, secretiongreenish purulent) and hyperemia of the edges (absent, small, moderate, large).

4.13.3 Reviews Laboratory

You results of the analysis laboratory must to be evaluated as the significance clinic. Will be carried out during the study to the following reviews laboratory:

- Complete blood count: will include hemoglobin, hematocrit, red blood cells, corpuscular volume mean corpuscular hemoglobin (MCH), mean corpuscular hemoglobin concentration (MCH), mean corpuscular hemoglobin concentration (CHCM), amplitude of distribution erythrocyte (RDW), count of leukocytes (WBC) with differential (neutrophils, lymphocytes, monocytes, eosinophils and basophils) and platelets;
- Blood glucose: glucose and hemoglobin glycated;
- Lipid profile: total cholesterol, low-density lipoprotein (LDL) levels will be assessed. lipoprotein of high density (HDL) and triglycerides;
- Liver function: Total bilirubin and fractions, aminotransferases (AST and ALT), alkaline phosphatase (FA), Gamma glutamyl transferase (Gamma-GT), Albumin, Total proteins and fractions and Coagulogram full (TTPA and TP with INR);
- Serologies: to HIV, Hepatitis B and W and HTLV;
- Function renal: urea, creatinine and clearance of creatinine;
- Profile inflammatory to response immune: dosage of cytokines serum what meet minimally youprofiles Th1, Th2 and Th17.

4.13.4 Dressings

Participants belonging to group 1 who will undergo cell therapy treatment will be instructed to change the secondary coverage whenever necessary, at least once a day, keeping a thin layer of moist gauze over the layer containing rayon gauze (dressing primary). After seven days will return to the center of search, where it will be withdrawn the bandage primary and secondary, washed the wound with 0.9% saline solution, applied Hydrogel with AGE Curatec®, dry gauze and finally crepe strip. You will be instructed to change the dressing at least once a day, maintaining the use of products according to the instructions given in the consultation, throughout the treatment period or until the wound is healed.

For participants in Group 2 – control, the wound must be cleaned with serum physiological saline solution 0.9%, apply the covering with Hydrogel containing AGE, dry gauze and finally crepe bandage. It will be oriented the to replace the dressing node minimum one time to the day, keeping the use of the products according to the guidance given in the consultation, during all the time of the treatment or until the wound be healed.

THE leave of then, all the patients will follow the same scale of visits.

4.13.5 Evaluation of Follow-up

Participants will be followed and assessed at 7, 30, 60 and 90 days after randomization. The closing assessment will be performed at visit 6 at 120 days. After your participation in the study you will be referred and monitored at the vascular surgery outpatient clinic at Hospital das Clínicas da FMB - UNESP (HC/FMB-UNESP). In this period visits extras they can occur, case there is need (Table 5).

Table 5 Timeline of Reviews and Visits

**Timeline of Activities – STUDY STEM-CELL I**							
**PROCEDURE-TOS**	**TRIAGEM**	**VISIT1**	**VISIT 2**	**VISIT 3**	**VISIT 4**	**VISIT 5**	**VISIT 6 – End**
	-1 to -30days	Day 0	7 days from the V1 (+/- 3 days)	30 days from the V1 (+/- 3 days)	60 days from the V1 (+/- 3 days)	90 days V1 (+/- 3 days)	120 days V1 (+/- 3 days)
Term of consen-free movement and clarified	X						
Consultation doctor: history, examination physical	X	X	X	X	X	X	X
Signs vital and an-tropometry	X	X	X	X	X	X	X
Medications con-comitants	X	X	X	X	X	X	X
Criteria of includes-they are and exclusion	X	X					
Pain scale (EVE)	X	X	X	X	X	X	
SF-36 and EQ-5D	X	X	X	X	X	X	
Assessment from the ulcer / photographic image-here / bandage	X	X	X	X	X	X	
Index Ankle Arm (ITB)	X			X	X	X	
Thermography	X		X	X	X	X	
Ultrasound ascular with Dop-pler	X			X		X	
Monitoring transcutaneous	X		X	X	X	X	
Collect of blood	X			X		X	
Serologies: HIV, Hepatitis B and C,HTLV	X						
Blood count With-complete	X			X		X	
Glucose	X			X		X	
Hemoglobin Gli-each	X			X		X	
Cholesterol total, LDL, HDL, triglycerides	X			X		X	
Bilirubin total and fractions	X			X		X	
TGO	X			X		X	
TGP	X			X		X	

Phosphatase alkaline	X			X		X	
Range-GT	X			X		X	
Proteins Totals, albumin and globulin	X			X		X	
Coagulogram: TP and TTPA	X			X		X	
Cytokines immuno modulators : Th1, Th2 and Th17	X			X		X	
Collect of sample of tissue adipose	X						
Randomization	X						
Application from CTM (G1) / Application of Cure-tivo(G2)		X					
Adverse events		X	X	X	X	X	X
Criteria of discon-continuation		X	X	X	X	X	
NOTE: you participants of group control they can to do the screening and visit 1 node same day since what you result states of the exams of screening already have been checked for the researcher.							

4.13.6 Criteria of discontinuation node study

To the discontinuations they can occur by the following criteria:

- You participants they can if to remove of study, if like this the want, the any moment andregardless of the reason, withdrawal of consent for the participant of study;
- For loss of follow-up;
- To evolve with major amputation in the member treated;
  Indication clinic immediate of amputation, worsens clinic the criterion of researcher, infections active from the ulcer
- No to present conditions clinics necessary to the realization of procedure of application from thecell therapy in period of one month after the date stipulated for application.

You reasons to the discontinuation of treatment must to be properly documented for the researcher. In the event of termination of the study, the researcher must ensure continuity of care doctor/hospital of participant from the search.

4.14 Outcomes

4.14.1Outcome primary

THE outcome primary the to be evaluated will be attached the occurrence of adverse events:

Security:

- Major: major amputations (amputations at leg or thigh level) or also called taxaof rescue of member (no occurrence of one amputation in relationship to the group comparator.

  Effectiveness:

- The primary efficacy endpoint of this study will be complete ulcer healing: assessed by total epithelialization of the ulcer; dressings and photographic images and no occurrence of amputation of member lower (rescue of member)

4.14.2 Secondary outcomeSecurity:

- Death, acute myocardial infarction (AMI), stroke, events thromboembolic as thrombosis venous deep (TVP) and embolism pulmonary (EP), picturesinfectious systemic and neoplasms;
- Infections local, bleeding, worsening from the pain and increase of the area from the wound

Effectiveness:

- Improved limb perfusion: assessed by thermography (significant increase in temperature limb > 0.3ºC), vascular ultrasound with Doppler (patency, waveform characteristic flow and flow velocity), ABI (increase in ABI value >/= 0.15 for participants with ABI<0.9), Capnography Transcutaneous (increase higher than 10% in the levels of The $_2$ measured and/or decrease of more than 10% in CO2 levels measured at a point at the base of the foot – base of the hallux andone ankle stitch back of the malleolus medial;
- Get better from the quality of life: evaluated by the SF-36 scale (Medical Outcomes Study 36 - Item Short - Form Health Survey);
- Reduction in pain: assessed by the Visual Analogue Pain Scale (VAS) and reduction in the use of painkillers (quantitatively) and qualitatively);
- Partial ulcer healing, assessed by partial ulcer epithelialization; dressings and images photographic (obtained by analyzing photographs using the area measurement tool software of use free ImageJ™);
- Get better of the level of perception health: assessed by scale EQ-5D by EuroQol Group.
- Get better clinic: observed for the improve of the parameters physiological (pressure arterial) and of laboratory parameters (blood glucose, glycated hemoglobin, HDL, LDL, total cholesterol, triglycerides, leukogram, hematocrit, uric acid, urea, creatinine, CRP, sodium, potassium, aminotransferases – TGO and TGP, total bilirubin and fractions, alkaline phosphatase – FA, Gamma glutamyl transferase – Gamma- GT, Albumin, Proteins totals and fractions and Coagulogram full – TTPA and TP with INR);
- Training of fabric of granulation in the ulcer the criterion of researcher;
- Change in the systemic inflammatory profile with a predominantly cellular immune response in detriment from the humoral, by quite of analysis of the cytokines immunomodulatory (Th1, Th2 and Th17).

Security / Effectiveness:

- Other adverse events: any occurrence not mentioned in the previous items and negative changes andclinically significant laboratory parameters (blood glucose, glycated hemoglobin, HDL, LDL, total cholesterol, triglycerides, leukogram, hematocrit, uric acid, urea, creatinine, CRP, sodium, potassium, TGO, TGP, bilirubin, PTF and coagulogram) and analysis histological tissue.

4.15 Collect and monitoring of the data

4.15.1 Collect and monitoring of the data of study clinical

THE verification and the authenticity from the quality of the data inserted in the record clinic electronics,

of therecords of data clinical node medical record electronic (MV) and of the documents printed to be will be carried outby data collection and monitoring procedures of the research team of the Research Unit Clinic of the Faculty of Medicine of Botucatu – UNESP (UPECLIN/ORPC), according to the monitoring plan,during the execution and at the closing of the clinical study.

The Principal Investigator of this clinical trial is responsible for taking all reasonable steps thatensure the proper conduct of the clinical study protocol in relation to ethics, adherence to the protocol andthe integrity and validity of the data registered us e-CRFs. Like this, the main function from the team of monitoring is to help the Researcher to maintain the quality technique, scientific, ethics and regulatory.

This clinical study will have an Investigator's File (according to the Document of the Americas, 2005), whose purpose is to create an Essential Document Management Plan. This plan will serve to specifically list which essential documents must be collected and how they will be maintenance during the Clinical Trial.

They are considered documents essential before of start of study:

A) Brochure of Researcher (already carried out);
B) Protocol and amendments signed;
C) Information given to the subject from the search, such as, the Term of Consent Free and Enlightened,documents of guidance to the participant during the study;
D) Documentation financial of study (budgets, contracts and insurance);
E) Letter of CEP/CONEP approval (Opinion Embodied);
F) Letter of Approval from the ANVISA;
G) Curriculum vitae and/or others documents relevant what highlight to the qualifications of (s)researcher(s) and sub-investigators;
H) Values normal/average (s) to procedure (s) and/or test (s) doctors/laboratory/technicians includednode protocol;
I) Certification of the laboratories what will perform you procedures of this study clinical;
J) Instructions to the handling of (s) product (s) under investigation and materials relating to the Rehearsal Clinical;
K) Records of shipping of products) under investigation and materials relative to the Rehearsal Clinical;
L) Certificate (s) of analysis of product (s) under investigation sent (s);
M) Decoding procedures for statistical analysis of data blinded clinical trial for analysisof data;
N) List main of randomization;
O) Report of monitoring pre-study;
P) Report monitoring of start of Rehearsal Clinical.

Will be considered essential documents during the execution of study:

A) Updates from the Brochure of Researcher;
B) Updates of Protocol;
C) Updates of information given to the participant from the search (Term of Consent Free andEnlightened and any others written information);
D) Approval of CEP/CONEP for to the updates of protocol and of the information given to the participant of research;
E) Curriculum vitae of new researchers and sub-investigators;
F) Updates of normal/mean values for procedure(s) and/or test(s) doctors/laboratory/technicians included in the

protocol;

G) Updates of values normal to procedures/tests doctors/laboratory/technicians;

H) Update from the certification of laboratory in the procedures that will be carried out to the protocol;

I) Documentation of shipping of products) under investigation and materials relative to the Rehearsal Clinical;

J) Certificate of analysis to the product of therapy cell phone advanced under investigation; K) Reports ofvisits of monitoring;

L) Communications relevant in addition of the visits to the local (letters, notes of meetings, notes of phone calls);

M) TCLEs signed and dated;

N) Documents source;

O) Copy of the chips clinics (CRFs) filled electronically;

P) Documentation of corrections in the chips clinics (CRFs);

Q) Notifications to the CEP on adverse events serious;

R) Notifications to the CEP of information of reports of security of product of therapy cell phone advancedunder investigation;

S) Biannual reports to the CEP and annual the ANVISA;

T) Registration of selection of the participants;

U) List of code of identification of the participants;

V) Registration of inclusion of the participants;

W) Registration that the product under investigation this being used as described node protocol;

X) Form of delegation of activities and record of signatures;

Y) Registration of storage of samples of CTM cryopreserved in laboratory during the period ofvalidity of study.

Will be considered essential documents to the closing of study:

A) All you documents listed previously;

B) Documentation of what the product of therapy cell phone advanced under investigation he was used of agreementwith the protocol;

C) Documentation of the final count of the investigational advanced cell therapy product receivednode local and applied to the participants, in addition than no he was applied to the participants;

D) Documentation from the undoing of the samples retained during the investigation;

E) List complete of codes of identification of the participants;

F) Certificate of audits what possibly have been carried out;

G) Report end of closing of Rehearsal Clinical to submission to the CEP/CONEP;

H) Documentation of allocation of treatment and decoding; I) Report end of Rehearsal Clinical toANVISA.

All the file of researcher he must be maintained archived put one period of 5 (five) years after the last approval of a registration request in Brazil (according to operational rule no. 1/2013 of the CONEP/CNS/MS and ANVISA RDC 09/2015), or for at least 2 years after discontinuation of the development clinical or conclusion formal of this development.

All you data clinical (reviews clinics, assessment from the ulcer, exams laboratory, dressing characteristics and adverse events) in addition to other data relevant to this study will be collected in standardized clinical records specifically for this study in electronic form (always with the performing *a back up*), also being carried out in physical

form and recorded in the electronic medical record of the research participant institution (of the System MV - MV Computing North East Ltd.).

4.15.2 Flat of Monitoring of study clinical

During the study, the team of professionals from UPECLIN/ORPC - UNESP will conduct periodic monitoring visits to ensure that the GCP protocol and guidelines are followed. In intervals regular during the study clinical, one representative from the team of center will accompany the progress of the study, participant and investigator adherence to protocol requirements. This team, linked for the secrecy professional, he must to maintain the confidentiality of all to the information medical personal data and personal identity (in accordance with confidentiality and personal data protection rules).The study center may be subject to IRB review and/or quality assurance audits and/or inspection by the respective regulatory authorities. Verification and authenticity of the quality of the data inserted in the electronic clinical record, clinical data records in the electronic medical record (MV) and documents printed will be carried out put procedures of collect and monitoring of the data for the same team from UPECLIN/ORPC - UNESP, according to the monitoring plan, during the execution and in the closing of clinical study.

THE Flat of Monitoring of this Rehearsal Clinical consists in:

A. Activities Training (training for the execution of research SOPs at all levels, training for the correct completion of the necessary documents; in addition to the registration of all training offered);
B. Provide a means of communication between the research center and the monitor, establishing that the point focal of contact will be the coordinator of search;
C. To establish the protocol of scheduling of visits;
D. Determine which documents must to be revised and collected, and check if the therapy cell phone advanced proposal in this Rehearsal Clinical is being correctly processed, stored and distributed;
E. To establish as and when must to be scheduled to the visits of monitoring, which documents mustto be revised and collected;
F. Verification of the forms of storage and evidence of quality from the therapy cell phoneadvanced;
G. Verification of the documents source and chips clinics (CRFs) (Figure 1).

Node closing of study, the visit of monitoring it will be carried out to to check the end of study, the undoing of samples, conference of documents, checking of file dead of the documents,preparation of the report end.

After to the due corrections, the bank of data it will be closed and forwarded to analysis statistic.

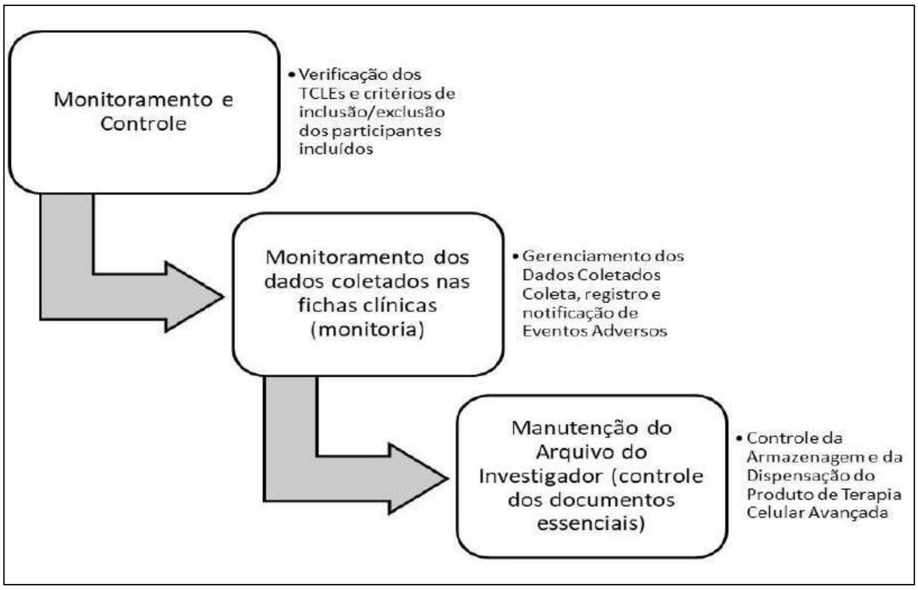

Figure 1 . Flow Monitoring of the data from the search clinic with therapy cell phone advancedproposal in this study.

4.15.3 Report and Management of Events Adverses

Adverse events will be collected during all participant follow-up visits. study clinical with therapy cell phone advanced (7 days, 30 days, 60 days, 90 days and 120 days) and registered both in the participant's clinical record and in the electronic evaluation form. A quantity is expected minimum number of adverse events related to injection or topical application and in the form of a stable biocurative, not being well established, to date, what the main manifestations of these events are, but they will be considered local adverse events (ulcer and peri-ulcer skin) and systemic (laboratory tests and symptoms clinical) with the start and end date of symptoms, intensity, whether it is severe or not, causality in relation study treatment (yes or no). According to the International Conference on Harmonization (ICH), adverse event (serious or not) is any undesirable clinical occurrence in a clinical research participant who receives or uses a product or treatment and who does not necessarily have a causal relationship with these. Like this, an adverse event can be any sign unfavorable and unintentional (including a found laboratory abnormal, symptom or illness temporally associated to the use of one product in investigation, considered related or no to the same). Conditions preexisting what suffer worsening during a study should be reported as adverse events. The event becomes serious when: results in death; threatens life; results in permanent, significant disability or impairment or to generate inability of carry out functions normal from the life; cause abnormality congenital; it is clinically significant for the assessment of researcher. Measures clinics will be taken based in treatments previously established to the treatment of events adverse according to they if present.

Events adverse serious will be announcements the Brazil Platform in until 24 hours of your occurrence.

4.16 Committee Independent of Monitoring of Security

This study will count with one committee independent of monitoring of security, compound putthree members of the host institution, who will evaluate the data on serious adverse events from this research. In confirmation of a serious adverse event, the coordinator of this clinical trial undertakes to declare them in the Brazil Platform, in until 24 hours.

THE committee it will be formed by the following members:

- Dr. Marcone File cork oak, Surgeon Vascular;
- Dr. Paula Angeleli Good de Camargo, Surgeon Vascular;
- Dr. Mariana Thais from the Silva Second, Surgery Vascular.

You data of events adverse or reactions adverse will be collected of the records of health from theinstitution of recruitment and, when necessary, of others professionals of health and of patient or representative.

The following information is collected: the type of event or reaction, including with regard to predefined events (allergy, local and systemic infection, loss of limb and death); date and time of onset; date and time of administration of the biocurative application; the extent of any causal link to the study; significance/severity of the event; measures taken regarding the subsequent use of the biocurative; and a summary of form free.

- Adverse events and reactions that, in the judgment of the site's principal investigator, are not part of the expected clinical course and may be related (at least possibly) to the study and are clinically significant or had sequelae serious.

Simple changes in the bacterial colonization of the ulcer (color, odor, and viscosity) are typical of these lesions, in addition to the occurrence of necrosis of the ulcer edges, minimal tissue loss, bleeding without the occurrence of hypovolemic shock and without significant changes in serum hemoglobin (decrease less than1g/mL) no will be reported as events adverse related to the procedures of study.

Surveillance period for adverse reactions and events, and allowing a safety margin, calling the attention of the researchers, he must include the period since the enrollment until 4 months after theadministration of biocurative, except put allergic reactions that will be limited to 7 days.

4.17 Process of report of security

Reportable adverse events and reactions will be communicated by site investigators to the principal investigator. This will generally occur in writing within 24 hours of the site investigator takingknowledge of event. The committee of management from the UPECLIN will evaluate all the reports of security received of the researchers. THE researcher boss will include those data in the Platform Brazil and will communicateto the authorities regulatory updates of security of agreement with the reports.

4.18 Flat of analysis interim

This study account with flat of analysis interim, what it will be carried out put one statistical independentwhen the study has already included half of the participants, at this point evidence will be considered benefits or harms of the treatment and if evidence of harm to the study participants is found it will be interrupted by reasons ethics.

4.19 Medications concomitants

There will be no restrictions on the use of concomitant medications, except medications topics to ulcers what interfere in the search.

4.20 Criteria of discontinuity of the participants

To the discontinuations they can occur by the following criteria:

- You participants they can if to remove of study, if like this the want, the any moment andregardless of the reason, withdrawal of consent by participant of study;
- For loss of follow-up;
- Occurrence of any kind of events adverse not expected.

You reasons to the discontinuation of treatment must to be properly documented for the researcher. In the event of termination of the study, the researcher must ensure continuity of caredoctor/hospital of participant from the search.

4.21 Methods statisticians

The results obtained through the primary and secondary outcomes will be tabulated weekly. in an electronic spreadsheet by the study monitoring team. A convenience sample of 40 is estimatedparticipants. The data will be initially evaluated using: 1- descriptive statistics, with analyses of median/variance and/or mean/deviation standard to variables quantitative and; 2- analysis of table of contingency for the variables categorical/qualitative. All the data monitored in the study for you groups G1 (CTM Application) and G2 (Dressing Application) will be statistically compared. The data quantitative data will be evaluated regarding their distribution (using the Shapiro-Wilk normality test) and compared using the T-test or the Wilcoxon test, depending on the type of distribution of the variables. For categorical variables, differences will be assessed using Pearson's Chi-square test or Fisher's exact test. Bonferroni coefficient and multivariate analyses will also be used. In all statistical tests, the significance level of the independent variable will be set at 5% ($p < 0.05$). Finally, the evaluation of the entire data set by multiple correspondence analysis is proposed. (popularly known as MCA, an acronym for the English term *Multiple Correspondence Analysis*). MCA and one technique exploratory and your application results in representations graphics of that set of data inthe same factorial plan, enabling the inference of correspondence between the information from Euclidean distances between these data on the plane, forming groupings (or *clusters*) of information correspondents. To your application, each variable quantitative it will be categorized of agreement with you quartilesof distribution of the your respective data.

Missing data are common in longitudinal clinical studies and may be related to: loss of participant; illnesses no related to the treatment in study; errors of transcription or typing;

inefficiency of the proposed treatment; lack of cooperation from the participant; poorly formulated questions in a questionnaire. To we deal with with this problem, we foresee the realization of strategies statistics good established as: analysis of complete cases; analysis of available cases and; statistical proceduresbased on imputation. The choice of the most appropriate method will be made after the collection of data and of custom shape the each type different of possible variable/data absent .

5. RESPONSIBILITIES

5.1Good Practices Clinics

This study clinical it will be driven in accordance with all to the laws nationals and guidelinesapplicable international standards; in accordance with the ethical principles defined at the 18th World Medical Assembly, Helsinki, 1964, and with all applicable amendments, and with the ICH Guidelines for Good Manufacturing Practices Clinics (BPC).

5.2Principles Ethical

This study was designed, planned and will be conducted in accordance with ethical principles for clinical research defined in the Declaration of Helsinki, in the Good Clinical Practice (GCP) guidelines and local regulatory requirements for clinical research. The study will begin as soon as approval is obtained by written by the Research Ethics Committee (CEP) and participants will be included only when the processof free and informed consent has been given and the free and informed consent form (TCLE) have been signed.

5.2.1 Laws and Regulations

This clinical study will be conducted in compliance with all international guidelines and laws applicable nationals, good as with all to the applicable guidelines.

5.2.2 Consent Free and Enlightened

The investigator, or the person designated by the Investigator and under his responsibility, must inform, clarify and guide the participant on all aspects relevant to the clinical study, including the information in writing as the approval by the CEP. Participants must receive the information available on the study, in language and under the terms so that may to understand.

Before any clinical trial procedure is performed on the participant, the ICFs must be signed, the name filled in and dated by the participants or their legal representative and by the person who led the discussion of free and informed consent. A copy of the signed and dated TCLE will be provided to the participant.

5.2.3 Committee of Ethics in Search (CEP) /CONEP

This clinical study protocol will be submitted to the CEP by the investigator and after its approval may start the procedures of the study.

5.2.4 Amendment to the protocol of study clinical

During the execution of the clinical study, any deviation, amendment or modification to the protocol will be submitted to the CEP prior to implementation, unless the change is necessary to eliminate an immediate risk to participants, in which case the CEP must be informed as soon as possible; as well as any event that may affect the safety of participants or when the change(s) involve only aspects logistical and administrative of study. To the updates from the Brochure of the Researcher will be sent to the ZIP code.

A progress report will be sent to the CEP, every six months, and a summary of the outcome of the study clinical node end of the study clinical.

Case the amendment demand changes to the TCLE, the researcher must receive the approval of the CEP in relationship to the TCLE revised, before of yours implantation. THE signature of participant he must to be obtained again,if necessary.

5.2.5 Deviations to the Protocol

In this study, one Detour to the protocol and considered any lack of greeting of the procedures or requirements defined in the version of protocol approved for the Committee of Ethics and ANVISA.

A deviation is considered minor if it does not have relevant implications for the integrity of the study, quality of the data or us rights and safety of participants of the research.

A deviation is considered the greater the compromise in data quality or study integrity, or that may affect the safety or rights of research participants. Major deviations from the protocol may include, but are not limited to: errors in the allocation of the investigational product, the use of unsuitable treatments, allowed by the protocol, low accession to the treatment, data no collected or loss of follow-up.

Any and all deviations (major or minor) and the reason for them must be documented in the medical record doctor.

5.3 Definition of the Source data

Source documents are defined as original documents, data and records. This includes, among others: others: hospital records, historical clinical, spreadsheets from source documents specific of the study, laboratory reports and requests, imaging tests, ECGs, medication dispensing records, impressions of computer, sources of information/data electronics and any other documentation related to the participant. The investigator may appoint other physicians as he or she deems appropriate, as sub-researchers to assistant in the driving of study clinical of agreement with the protocol. Yousub-researchers will be supervised for the and will work under the responsibility of researcher. THE researcher will provide to the sub-researchers one copy of protocol of study clinical and all to the necessary information. The data collected in this study will only be used for the purpose(s) of thestudy.

5.3.1 Retention of record node center of study – File of documents

The researcher and his team will keep all study documentation confidential and will take stepsto avoid the undoing early or accidental of the documents.

documents will be archived at the research center for 5 years after completion or discontinuation of this study clinical to allow what you data be investigated, monitored and or inspected.

The researcher will make all source documents and other records of the study available to members of CEP and or regulatory authorities nationals, if necessary.

5.4 Compensation of Safe

The Researcher certifies that he will provide a civil liability insurance policy for this study. This policy of safe this in accordance with local laws and requirements.

5.5 Publications and Communications

The researcher undertakes to publish and disseminate his/her results. This study represents a joint effort of the researcher, investigators, study team, Institution and MS and, as such, they agree that review should be carried out by all parties in the preparation of scientific documents for publication or presentation. The main findings of the study will be submitted for publication in a journal indexed. The researcher reserves the right to choose the first and last author of the publication. The others members of the study team will be named co-authors of the main article, considering their participation effective node development and execution of study, respecting yet the number of authors maximum established by the journal where it will be published. Study team members may submit proposals of publications and presentations parallels of the results of this study clinical. Node however, all to the publications and presentations proposals by the researchers/members from the team will be analyzed for the researcher.

5.6 Control of Quality and Guarantee of Quality

During the conduct of the study, UPECLIN will conduct periodic monitoring visits to ensure that the GCP protocol and guidelines are followed. At regular intervals during the study clinical, one representative from the team of center will accompany the progress of study, the adhesion of participant and investigator to the protocol requirements. These monitoring visits will include, among others, others, the review of the following aspects: TCLE, recruitment and monitoring of the participant, reporting and SAE documentation, AE documentation, participant adherence to treatment, medication use concomitant and quality of the data.

According to the ICH guidelines for Good Clinical Practice, the monitoring team shouldto check the accuracy of the entries node e-CRF in relation to the source documents. THE TCLE will include one statement that the participant allows the duly authorized researcher staff, the Research Committee, Research Ethics Committee (REC) and regulatory authorities have direct access to original medical records that corroborate the data from the e-CRFs (e.g., participant's medical file, diaries, records original laboratory data, etc.). This team, bound by professional secrecy, must maintain confidentialityof all personal medical information and personal identity (in accordance with confidentiality and rules of protection of data personal).

The study center may be subject to IRB review and/or quality assurance audits. and/or the inspection by respective authorities regulatory.

THE Researcher of this study clinical and responsible put carry out all to the steps reasonable what ensure the proper conduct of the clinical study protocol in relation to ethics, adherence to the protocol andthe integrity and validity of the data registered us e-CRFs. Like this, the main function from the team of monitoring is to help the Researcher to maintain the quality technique, scientific, ethics and regulatory.

6. RESULTS EXPECTED AND YOURS RELEVANCE TO YOU SICK AND THE SUS

6.1 Results expected

The results and the contributions he has two aspects to be considered:

Among you results expected, they are:

- Implementation of the Cell Processing Center of the HC of FMB – UNESP. It is expected that the possibility implementation of the CPC of HC UNESP will have an enormous scientific and technological impact on the approaches stem cell therapies, in addition to allowing the CPC to reach a large-scale production level of *in vitro* expanded stem cells . The implementation and execution period of this first project will be will serve as a basis for leading new studies with advanced cell therapy. For this purpose, only the execution of this project already provides the measurement of this goal.
- Dissemination of cell therapy technology at secondary, higher education, postgraduate and undergraduate levels among health professionals and related professionals. The impact of the dissemination of information should be reflected both in training professionals with critical sense and adapted to the time of technological evolution that scienceprovides. The form of measurement will be obtained by assessing compliance with extension goals andof the dissemination goals being fulfilled according to the schedule previously presented.
- For study participants, the project aims to provide an innovative and cutting-edge scientific therapy. fic, with the offer of an alternative that can improve the chances of not amputating the limb affected, in addition to providing a possible improvement in quality of life and overall health. The results of this study regarding the salvage of affected limbs, the rate of wound healing, the quality of life questionnaires, blood tests and other exams will support the actual results for participants regarding the effectiveness of the proposed treatment (advanced cell therapy) within the expected deadlines to this study.
- For the SUS and public health policies, advanced cell therapy urgently needs scientific proof through studies carried out in national territory, to support the breaking of some paradigms. but that in countries in Europe, Asia and the USA they have already consolidated themselves as an alternative. This cannot be mentionedsurable by simple numbers from a single study, but if at the end of this study and this line of financing- proposed by CNPq in partnership with DECIT, it is possible to observe the proposition of new pro- research projects with advanced cell therapy, will already represent an advance in this sense of popularizationof technology in Brazilian soil.

- For technology pricing, carrying out this study can contribute to providing value. average resources necessary for such treatment and the establishment of rules for investmentscoughs of this nature.
- For researchers involved in participating in scientific conferences, publishing articles and contact with researchers from other national and international institutions will provide growthof the scientific group, establishment of the scientific group, promotion of cutting-edge Brazilian research and in- internationalization.
- It will provide advanced technical training for the members of this research, which will be measurable by DT bags that will be provided and the increment curriculum and practical of technicians of this research.
- For the family members and caregivers of these patients, the treatment of the participants may represent a relief from suffering in seeing a patient suffering without any alternative treatment. This cannot be measured in this study, but will reflect in the dissemination of advanced cell therapy as a therapeutic alternative. real therapeutics in society, will increase visibility and bring hope associated with the vision of a University and of the SUS committed to promote the improvement from the health from the population.
- Possible obtaining of patent and license for advanced cell therapy product at ANVISA for future commercialization. This goal can be achieved depending on the results that are found during therer of the study. The measurement is made through the number of patent filing processes and the number of protocol of record and license of product of therapy advanced cell phone in the ANVISA.
- It is expected, put end, what one protocol of treatment with therapy cell phone advanced to DAP in ICMwith imminent risk of limb loss is established at the end of the project. This data will also be measurable through of the publications scientific related the this study.

6.2 Alignment with policies public of health

This project is found aligned with the policy public of health in illnesses chronicles no transmissible diseases (NCDs), in order to promote actions to contain potential risk factors for illness arterial peripheral and too much illnesses cardiocirculatory (instruction to the participants, family members, caregivers, patients of the peripheral arterial disease outpatient clinic at HC FMB UNESP, promoting then the reduction in mortality rates, reduction in hospitalization rates due to complications ofillness arterial peripheral and of the ulcers arterial, promoting treatment and hospitalization effective to the NCD treatment (in this case, use of advanced cell therapy for patients with arterial ulcers and high risk of limb loss). It still aligns with the premise of treating high-cost, high complexity patients with one alternative of high technology, promoting actions what will be executed immediately to what provide results effective in terms of quality of life. This form, intended avoid the worsening of the individual's health conditions in those with peripheral arterial disease and the plan of study is linked to actions that promote the improvement of the information system, training of professionals of health, financing suitable, obtaining of medicines and technology essential.

6.3 Impacts to the yours

This is an alternative as a clinical treatment solution for patients in advanced stages. from the DAP, where present ischemia criticism of members and ulcer arterial without possibility of revascularization, which greatly increases the likelihood of limb amputation. Very few studies clinical trials with advanced cell therapy have been carried out in Brazil and only recently have we had a clearer regulatory framework in this regard, with the publication of RDCs that allow for adequate management of this technology. This study can help strengthen cell therapy in the country, probably being more an alternative that may be made available by the SUS and that depends on scientific proof. The patients who will participate in this study, naturally generate a great cost for the SUS, since they arecarriers of a serious illness, associated with other comorbidities that lead to continuous consumption of painkillers, prolonged sequences of treatments with very expensive antibiotics, costs of dressings, transportation costs, serial hospitalizations and finally culminate in the amputation of the limb, becoming mostly dependent on the Brazilian social security system, losing years of labor productivity, compromising families with too much care, as well as causing frustration and psychological problems serious in these patients, more once, burdening the SUS.

In a therapeutic sense, advanced cell therapy can provide an evolution towards frontier of knowledge and therapeutic possibilities offered by the SUS, reinforcing its value in front of population user.

Thus, despite the scarce scientific literature available on this subject and the lack of a clinical study similar to the study proposed in this protocol, there is scientific literary support enough what guarantees what this study no if treats of one experimentalism unreasonable.

7. Cost-Effectiveness

The preparation of these cost-benefit calculations (cost/effectiveness) will be done using modelsclinical decision and economic evaluation by developing a decision tree and, if possible, carrying outtion of models of Markov. Will be taken in consideration:

a. Cost Identification - all costs associated with the new procedure will be listed, includingmedical equipment, staff training, facilities, consumables and operating costs.onals.
b. Benefits Clinical - will be described you benefits clinical expected of procedure, as improvement in the health of the patient, reduction of complications it is better quality of life
c. Analysis of Costs Direct - will be calculated you costs direct, as buy of equipment, maintenance, training of guys and costs of procedure in yes.
d. Analysis of Costs Indirect - will be considered all you costs indirect, as space physical,energy, safe and costs administrative.

e. Economic Benefits - economic benefits will be assessed, such as reduced hospitalizations hospital costs, reduced visits to the doctor or economic gains due to recovery from productivity of patient.
f. Cost-effectiveness analysis - the relationship between total costs and clinical benefits will be calculated and expected economic results. This can be expressed in terms of cost per unit of output, ascost per life saved, per year of life gained or per quality of life improved depending on the dataof those found. Remembering that patients will be initially monitored for 4 months, but to this data will be consulted put one year.
g. Comparison with alternatives - the new procedure will be compared with the existing therapy, todetermine whether and more cost-effective.
h. Risk Assessment - potential risks will be assessed, such as uncertainties in benefits or costsunexpected, acting to decrease them.
i. Communication and decision making - findings will be communicated clearly and concisely to parts interested, as doctors, managers of health and takers of decision to what may support to adoption of the new procedure.
j. Continuous monitoring and evaluation - monitoring to track costs and benefits offar away of time

8. Impacts and contributions to the complex economic-industrial from the health

THE introduction of therapy cell phone advanced can bring several benefits to the health system national:

- Improved Quality of Care – the new procedure can improve the quality of care medical care, offering more effective and less invasive treatment for patients in risk of amputation of members inferior;
- Cost Reduction – New cell therapy technology can reduce costs in the long run, especially if it is proven to be more effective in preventing complications or reducingtime of hospitalization;
- Advances Technological - THE search and the development of new procedures in therapy cell phonecan boost the medical and technology industry in the country, creating jobs and opportunities business, in addition of to be another option that can be offered to the patients served for the SUS;
- International Recognition – innovative procedures in cell therapy can attract international recognition. attention International, generating prestige to the community medical and scientific Brazil;
- Investment Attraction – innovations in health can attract investments from institutions research, pharmaceutical companies and foreign investors, boosting the economy at a local, regional and national. We will probably see the emergence of at least one local startup in this area of technological development in health;
- Strengthening from the Search - the development of new procedures in therapy cell phone strengthens the collaboration between institutions academics and medical nationals and international, strengthening the search in health in the country;

- Reducing Dependence on Foreign Technologies - by developing our own procedures in cell therapy, Brazil can reduce its dependence on imported technologies and products, saving foreign exchange for the country;
- Universal Access - if the procedure is effective and accessible, it can contribute to the promotion of access universal the health in the country.

In short, innovation in medical procedures in Brazil can bring a series of benefits for the health system, provided that it is accompanied by a rigorous evaluation and an appropriate strategyof implementation.

9. CHARACTER INNOVATIVE DO PROJECT

THE project of search clinic with therapy cell phone advanced proposed node present study intends perform the treatment of patients with peripheral arterial disease (PAD) with critical limb ischemia (ICM), with arterial ulcer in the lower limb for more than 3 weeks, without the possibility of limb revascularization, incomplete revascularization, or revascularization with restenosis or occlusion (Fontaine IV and Rutherford 5).

The product of this study consists of an advanced cell therapy with an innovative protocol that was developed put this team of study, the which will use stem cells mesenchymal (CTM) adults autologous obtained of tissue adipose abdominal and diluted in plasma autologous in association with the application of the same CTM in the form of a stable biocurative, in a single treatment session. Both the MSCs that will be injected perilesionally as the stable biodressing containing the same cells must provide a large supplement of MSCs both at the edges and on the surface of the lesion, which should play a paper modulator node inflammatory process and reparatory from the injury.

9.1 Study of feasibility patent

THE analysis prospective of process patent holder of process involved in this project, including the stable biocurative using autologous plasma and the use of Fibracol collagen membrane for feasibility of topical CTM on arterial ulcers, to the detriment of the number of scientific articles found, which shows high maturity of the technological route. The study of the prospective analysis of the patent showed that the technology used, despite its low level of technological maturity, has a character innovative before the Institute National of Property Intellectual, INPI.

Additionally, he was carried out one search in bases of data of patents and they were identified you following documents:
[1]https://patents.google.com/patent/KR20090086066A/en?q=stem+cell+AND+wound+heal&oq=stem+cell+AND+wound+heal
[2]https://patents.google.com/patent/US20030021850A1/en?q=stem+cell+AND+wound+heal&oq=stem+cell+AND+wound+heal

This form, you documents identified precede the technology and with certain similarity with thetechnology proposal in this study, although the technology proposal advance in the part technique of management from the therapycell phone in patients with DAP and ischemia criticism put increase you means of application of the cells, bothby injections or through topical use with a stable dressing, being an innovative and inventive technology. Considering the benefits of technology presented, recommended advance node development from the technology to if to achieve scales bigger of maturity to to have appeal commercial in applications,

mainly, node sector of therapy cell phone advanced.

10. PLANNING OF MANAGEMENT OF RISKS:

Frame 1 – Management of risks relative the execution of present study.

Event of Risk	Nature ofRisk	Cause	Consequence	Identified-cation ofLevel	Prevention/ Monitor-ment	Procedures of minimization
No reached-turn the naked- mere of participating- you have foresee-coughs in study	Operational and Financial	Aggravation resulting from the condition cli- unique of sick- you, amputation and occurrence of death	No greetings to of the deadlines provided for in the timeline	Low	Revaluation of the processes SOS employ- cattle in the recruitment- to	Act to promo- see a better control clinical of the participants
Contami- nation of culture of cells ofa parti- participant or Contami- nation of LPC	Technical and Financial	Presence of microorganisms- we in the cultures cellular ras Presence of microorganisms- we in several follow-ups of culture and products usedzed	Delayed start of the treatment and new procedure expansion ment-they are cell phone Delayed start of the treatment and decontaminationof LPC	Low	Follow suit- squarely the manual ofGood Practices Labora- cases tools for cell culture home and attention to the protocols the opera- onais pa- drão	Protocol review the operational ones standard and of all manipulation phases tion of tissues and cultures cell phones

It is worth noting that the prevention of events related to loss of culture due to contamination, despite to

promote some increase in costs, it is already foreseen and minimized in the protocols that will be usedin this clinical trial. Regarding possible delays in inclusion or treatment of participants, can be state that they are unlikely to generate delays in the overall schedule, since the protocol already provides for a reasonable margin temporal to the conclusion of this study. As to the establishment of number of participants who complete the study, it is believed that the impact of the treatment will be seen even with 50% of the proposed sample, but if this minimum number of participants (n=20) is not reached, new participants will be included until this minimum number is reached so that there is no compromise of the analyses of results.

11. PROPERTY INTELLECTUAL

THE property intellectual and of researcher coordinator of study.

This study he has potential to to generate one patent (dressing) what he must to be deposited in name of researcher and from the institution thirst of study (UNESP).

THE cost referring to the deposit initial from the patent will be of the institution thirst (UNESP/FMB).

To the licensing and transfer from the technology generated put that study and possiblypatented, we intend carry out a public-private partnership.

THE policy to eventual earnings economic earned with the exploration from the invention generated for the study it will be shared between the researcher, the institution (UNESP/FMB) and the Ministry from the Health (MS).

THE researcher no he must be under obligation of patent, to develop, to market or use ofother form you results of the study clinical.

If necessary, the investigator and/or sub-investigators and study and research center teams must provide all assistance requested by the Institution and the Ministry of Health, at their expense, to obtaining and defense of any patent, including the signature of documents cool.

12. FLAT OF WORK

12.1 Identification of object the to be executed - Flat of work:

Table 6. Timeline

GOAL	ACTIVITIES	DATE
Start of clinical trial execution	You patients what they are served node outpatient clinic of surgery vascular of HC/FMB-UNESP/UNESP and what answer you criteria of eligibility will be invited to participate in the researchwhat it will be executed in the Unit of Search Clinic from the Faculty of Medicine of Botucatu (UPECLIN – FMB/UNESP).	06/01/2025
End of period of inclusion of participants in the study	In this stage expected what the size sample proposed you have been reached, then new participants no may more to be included. You patients previously included will continuebeing served according to timeline of study.	07/01/2027

Closing of the study clinical	THE closing of study clinical will occur when the last participant included performs the last study visit, according to timeline. After the participation in the study, the participants will be oriented and accompanied node outpatient clinic of surgery vascular of HC/FMB-UNESP/UNESP, with the same team doctor what led the study.	12/20/2027
Preparation and submission offinal reports	The reports will be elaborated and sent according to the standards of regulatory bodiescompetent.	12/20/2027

12.2 Preparation, preparation and shipping of dossiers regulatory

Prepare clinical trial protocol, researcher brochure, Development dossier Institutional Advanced Therapy Product Clinician (DDCTA) (new technology with CTM), term of consent free and enlightened, statements institutional; register protocol of rehearsal clinical in open access virtual platform (Clinical Trials; REBEC); ethical and regulatory submissions, develop SOP, Case Report Form (CRF), terms of agreement/contracts, prepare dossiers according to the standards of ANVISA and CEP/CONEP and to send.

12.3 Start from the execution of study clinical

Patients who are treated at the vascular surgery outpatient clinic at HC/FMB-UNESP/UNESP and who meet the eligibility criteria will be invited to participate in the research that will be carried out in Unit of Clinical Research from the Faculty of Medicine of Botucatu (UPECLIN – FMB/UNESP).

12.4. Execution of study clinical

Continuity in the inclusion and care of already randomized participants, carrying out monitoring,according to the Good Clinical Practice Manual (GCP/ICH), to ensure the quality of the data collected, filling of bank of data, preparation and shipping of reports biannual to the ZIP code, update of documentation in the ANVISA (if necessary), between others.

12.5 End of period of inclusion of participants in the study

At this stage it is expected that the proposed sample size has been reached, as new participantscan no longer be included. Previously included patients will continue to be treated asstudy schedule.

12.6 Closing from the execution of study clinical

The clinical study will be closed when the last participant included performs the last study visit, according to schedule. After participating in the study, participants will be guided and monitored at the vascular surgery outpatient clinic at HC/FMB-UNESP/UNESP, with the same team doctor who led the study.

12.7 Management of the data and statistic

The verification and authenticity of the quality of the data entered in the clinical record will be carried out by UPECLIN/ORPC, according to the monitoring plan, during the execution and at the end of the study clinical. After the due corrections, the bank of data it will be closed and forwarded to analysis statistic.

12.8 Preparation and shipping of reports

Reports will be prepared and sent in accordance with the standards of the competent regulatory bodies. (ANVISA, CEP/CONEP). The accounts and presentation of results will be made according to guidelines of the financiers of project.

12.9 Participation in events scientific disclosure of the results

The project coordinator and team members will participate during this period in meetings with government agencies with the aim of presenting the new technology/therapeutics and its possibility of insertion into the Unified Health System. We believe that the study will make it possible to make available yet anotheralternative treatment for these patients, contributing to a reduction in deaths, surgeries lower limb amputations, length of hospital stay and improvement in quality and life expectancy of people with illness peripheral arterial.

Seminars of follow-up and assessment of project, presentation in meetings scientific as congresses nationals and international, elaboration of articles to publication in magazines scientific conceptualized must occur during and to the end of project.

13. SUMMARY OF THE INVESTMENTS

COST	CAPITAL	SCHOLARSHIPS	TOTAL
R$ 2,566,20.00(58%)	R$1,159,000.00 (26%)	R$ 6888000.00 (16%)	R$ 4,414,000.00 (100%)

14. COUNTERPART FROM THE INSTITUTION

THE counterpart to the driving of this study understand you resources humans and infrastructure institutional (HC-FMB-UPECLIN/UNESP).

15. REFERENCES

1. Mayerl C, Lukasser M, Sedivy R, et al. Atherosclerosis research from past to present—on the track of two pathologists with opposing views, Carl von Rokitansky and Rudolf Virchow. Virchows Archive 2006;449:96 -103.
2. Yusuf S, Reddy S, Ounpuu S, and al. Global burden of cardiovascular diseases: part I: general considerations, the epidemiological transition, risk factors, and impact of urbanization. Circulation 2001;104:2746 -2753.
3. Yusuf S, Reddy S, Ôunpuu S, et al. Global burden of cardiovascular diseases: Part II: variations in cardiovascular disease by specific ethnic groups and geographic regions and prevention strategies. Circulation 2001;104:2855 -2864.
4. Sanderson JE, Mayosi B, Yusuf S, and al. Global burden of cardiovascular disease. Heart 2007;93:1175 .
5. Rose G. Epidemiology of Atherosclerosis. BMJ: British Medical Journal: International Edition 1991;303:1537 -1539.
6. Herrington W, Lacey B, Sherliker P, and al. Epidemiology of atherosclerosis and the potential to reduce

theglobal burden of atherothrombotic disease. Circulation research 2016;118:535 -546.

7. Norgren L, Hiatt WR, Dormandy ALREADY, and al. Inter-society consensus for the management of peripheralarterial disease (TASC II). Journal of vascular surgery 2007; 45:S 5-S67.
8. Criqui MH, Aboyans V. Epidemiology of peripheral artery disease. Circulation research 2015;116:1509-1526.
9. Smith GD, Shipley MJ, Rose G. Intermittent lameness, heart disease risk factors, and mortality. The Whitehall Study. Circulation 1990;82:1925-1931.
10. Lane R, Harwood THE, Watson L, and al. Exercise for intermittent claudication. The Cochrane Library 2017.
11. Channel W, McGee D. Update on some epidemiological features of intermittent lameness: the Framingham Study. Journal of the American Geriatrics Society 1985;33:13-18.
12. Virag R, Zwang G, Dermange H, and al. Vasculogenic impotence: the review of 92 cases with 54 surgicaloperations. Vascular Surgery 1981;15:9-17.
13. Sheol SH. Leriche Syndrome. Journal of Medicine 2017;18:128 .
14. Halperin JL. Evaluation of patients with peripheral vascular disease. Thrombosis research2002; 106:V 303-V311.
15. Lambert M, Belch J. Medical management of critical limb ischaemia : where of we stand today? Journal of internal medicine 2013;274:295-307.
16. Ouriel K. Peripheral arterial disease. The lancet 2001;358:1257-1264.
17. Rutherford RB, Baker JD, Ernst W, and al. Recommended standards for reports dealing with lower extremity ischemia: revised version. Journal of vascular surgery 1997;26:517 -538.
18. Fontaine R, Kim M, Kieny R. Surgical treatment of peripheral circulation disorders. Helvetica chirurgica minutes 1954;21:499-533.
19. Jaff MR, White CJ, Hiatt WR, et al. An update on methods for revascularization and expansion of the TASC injury classification to include below-the-knee arteries: the supplement to the Inter-Society Consensus for the Management of Peripheral Arterial Disease (TASC II). Annals of vascular diseases 2015;8:343-357.
20. Treiman GS, Oderich GS, Ashrafi A, et al. Management of ischemic heel ulceration and gangrene: an evaluation of factors associated with successful healing. Journal of vascular surgery 2000;31:1110 -1118.
21. Federman DG, Ladiiznski B, Dardik A, et al. Wound healing society 2014 update on guidelines for arterial ulcers. Wound Repair and Regeneration 2016;24:127 -135.
22. Silva LR, Fernandes GM, Morales NAKED, and al. Results of One-Stage or Staged Amputations of LowerLimbs Consequent to Critical Limb Ischemia and Infection. Annals of vascular surgery 2018;46:218 -225. 23.Alavi THE, Sibbald ID, Phillips TJ, and al. What's new: Management of venous leg ulcers: Treating venous leg ulcers. Journal of the American Academy of Dermatology 2016;74:643 -664.
24. Bergqvist D, Lindholm C, Nelzén O. Chronic leg ulcers: the impact of venous disease. Journal of vascular surgery 1999;29:752-755.
25. Singer AJ, Tassiopoulos THE, Kirsner RS. Evaluation and management of lower extremity ulcers. NewEngland Journal of Medicine 2017;377:1559-1567.
26. Packet D, Phalange V. Leg ulcers. Clinics in geriatric medicine 2002;18:77-88.

27. Phillips TJ, Dover JS. Leg ulcers. Journal of the American Academy of Dermatology 1991;25:965 -987.
28. Abbot LP, Lastoria S, of Almeida Rollo H. Venous ulcer: clinical characteristics and risk factors. International journal of dermatology 2011;50:405-411.
29. Ferreira AM, Bogamil DD, Tormena PC. Nurses and wound care: in search of autonomyof Careful. Arch. Science Health 2008;15:105-9.
30. Grey JE, Harding KG, Enoch S. Venous and arterial leg ulcers. BmJ 2006;332:347-350.
31. Sprengers RW, Lips DJ, Moll FL, and al. Progenitor cell therapy in patients with critical limb ischemiawithout surgical options. Annals of surgery 2008;247:411 -420.
32. Isaac W, of Slope PRS, of Trench FMP, and al. Process of cure of the wounds: healing physiological. Magazine of Medicine 2010;89:125-131.
33. Diegelmann RF, Evans MC. Wound healing: an overview of acute, fibrotic and delayed healing. Front Biosci 2004;9:283 -289.
34. Barrientos S, Stojadinovic Oh, Golinko MS, et al. Growth factors and cytokines in wound healing. Woundrepair and regeneration 2008;16:585-601.
35. Velnar T, Bailey T, Smrkolj V. The wound healing process: an overview of the cellular and molecular mechanisms. Journal of International Medical Research 2009;37:1528-1542.
36. Schreml S, Szeimies R, Prantl L, and al. Oxygen in acute and chronic wound healing. British Journal ofDermatology 2010;163:257 -268.
37. Tokuda Y, Crane S, Yamaguchi Y, and al. The levels and kinetics of oxygen tension detectable at thesurface of human dermal fibroblast cultures. Journal of cellular physiology 2000;182:414-420.
38. RAZIYEVA, Kamila and al. Immunology of acute and chronic wound healing. Biomolecules, v. 11, n. 5, p. 700, 2021
39. Grazul -Bilska AT, Johnson ML, Bilski JJ, et al. Wound healing: the role of growth factors. Drugs Today (Boat) 2003;39:787 -800.
40. Singer AJ, Clark FROG. Cutaneous wound healing. New England journal of medicine 1999;341:738 - 746.41.Dhivya S, Padma VV, Santhini AND. Wound dressings–a review. BioMedicine 2015;5.
42. Fan K, Tang J, Scandon J, and al. State of the art in topical wound-healing products. Plastic and reconstructive surgery 2011;127:44 S-59S.
43. George Broughton I, Janis JE, Attinger CE. A brief history of wound care. Plastic and reconstructive surgery 2006;117:6S-11S.
44. Fonder MA, Lazarus GS, Cowan DA, et al. Treating the chronic wound: a practical approach to the care of nonhealing wounds and wound care dressings. Journal of the American Academy of Dermatology 2008;58:185-206.
45. Goossens THE, Cleenewerck M. New wound dressings: classification, tolerance. European journal of dermatology: EJD 2010;20:24.
46. Bakker K, Apelqvist J, Schaper NC, and al. Practical guidelines on the management and prevention of thediabetic foot 2011. Diabetes/metabolism research and reviews 2012;28:225 -231.
47. Futrega K, King M, Lott WB, and al. Treating the whole not the hole: necessary coupling of

technologiesfor diabetic foot ulcer treatment. Trends in molecular medicine 2014;20:137 -142.

48. Naruse K, Hamada Y, Nakashima AND, and al. Therapeutic Neovascularization Using Cord Blood–DerivedEndothelial Progenitor Cells for Diabetic Neuropathy. Diabetes 2005;54:1823-1828.
49. Strem BM, Hicok KC, Zhu M, and al. Multipotential differentiation of adipose tissue-derived stem cells. The Keio journal of medicine 2005;54:132 -141.
50. Lee RH, Kim B, Choi I, et al. Characterization and expression analysis of mesenchymal stem cells from human bone marrow and adipose tissue. Cellular physiology and biochemistry 2004;14:311 -324.
51. Winkler IG, Wiercinska E, Barbier V, et al. Mobilization of hematopoietic stem cells with highest self-renewal by G-CSF precedes clonogenic cell mobilization peak. Experimental hematology 2016;44:303 - 314. e1.
52. Baksh D, Song L, Tuan A. Adult mesenchymal stem cells: characterization, differentiation, and application in cell and gene therapy. Journal of cellular and molecular medicine 2004;8:301-316.
53. Dominici M, The White K, Mueller I, et al. Minimal criteria for defining multipotent mesenchymal stromal cells. The International Society for Cellular Therapy position statement. Cytotherapy 2006;8:315 -317.
54. Zuk PA, Zhu M, Ashjian P, et al. Human adipose tissue is a source of multipotent stem cells. Molecular biology of the cell 2002;13:4279 -4295.
55. Bourin P, Bunnell BA, Casteilla L, et al. Stromal cells from the adipose tissue-derived stromal vascular fraction and culture expanded adipose tissue-derived stromal/stem cells: the joint statement of the International Federation for Adipose Therapeutics and Science (IFATS) and the International Society for Cellular Therapy (ISCT). Cytotherapy 2013;15:641 -648.
56. Rodbell M. The removal and metabolism of chylomicrons by adipose tissue in vitro. Journal of Biological Chemistry 1960;235:1613-1620.
57. Dai R, Wang Z, Samanipour R, et al. Adipose-derived stem cells for tissue engineering and regenerative medicine applications. Stem cells international 2016;2016.
58. Gao F, Chiu S, Motan D, et al. Mesenchymal stem cells and immunomodulation: current status and future prospects. Cell death & disease 2017; 7:e 2062.
59. Wang Y, Chen X, Dog W, and al. Plasticity of mesenchymal stem cells in immunomodulation: pathological and therapeutic implications. Nature immunology 2014;15:1009 .
60. Glenn JD, Whartenby KA. Mesenchymal stem cells: emerging mechanisms of immunomodulation and therapy. World Journal of stem cells 2014;6:526.
61. Tateishi-Yuyama E, Matsubara H, Murohara T, et al. Therapeutic angiogenesis for patients with limb ischaemia by autologous transplantation of bone-marrow cells: a pilot study and a randomized controlled trial trial. The Lancet 2002;360:427-435.
62. Kawamura A, Horie T, Tsuda I, et al. Clinical study of therapeutic angiogenesis by autologous peripheral blood stem cell (PBSC) transplantation in 92 patients with critically ischemic limbs. Journal of Artificial Organs 2006;9:226 -233.
63. Horie T, Onodera R, Akamastu M, et al. Long-term clinical outcomes for patients with lower limb ischemia implanted with G-CSF-mobilized autologous peripheral blood mononuclear cells. Atherosclerosis

2010;208:461-466.

64. Jiang X, Zhang H, Teng M. Effectiveness of autologous stem cell therapy for the treatment of lower extremity ulcers: the systematic review and meta-analysis. Medicine 2016;95.
65. Tsuji W, Ruby J, Marra K. Adipose derived stem cells: implications in tissue regeneration. World J Stem Cells 2014;26:312-321.
66. Dainesi SM. The probe methodology can be considered an alternative to randomized studies double-blind? Magazine from the Medical Association Brazilian 2010; 56.2:132.
67. National Health Surveillance Agency (Brazil). Resolution of the Board of Directors - RDC No. 214, of 7 February 2018. Provides for Good Practices in Human Cells for Therapeutic Use and search clinical, and from the others provisions. Official Gazette of Unity No. 36, of 22 of February of 2018.
68. National Health Surveillance Agency (Brazil). Resolution of the Collegiate Board - RDC No. 260, of December 21, 2018. Provides the rules for conducting clinical trials with a product of investigational advanced therapy in Brazil, and provides other measures. Official Gazette of the Union No. 249 of 28ten 2018.
69. Bustin, S. a, Benes, V., Garson, J. a, Hellemans , J., Huggett, J., Kubista, M., … Wittwer, C. T. (2009). The MIQE guidelines: minimum information for publication of quantitative real-time PCR experiments. *Clinical Chemistry* , *55* (4), 611–622. https://doi.org/10.1373/clinchem.2008.112797
70. Fleige, S., & Pfaffl, MW (2006). RNA integrity and the effect on the real-time qRT -PCR performance. *Molecular Aspects of Medicine* , *27* (2–3), 126–139. https://doi.org/10.1016/j.mam.2005.12.003
71. Ye, J., Coulouris, G., Zaretskaya, I., Cutcutache , I., Rozen, S., & Madden, T. L. (2012). Primer-BLAST: a tool to design target-specific primers for polymerase chain reaction. *BMC Bioinformatics* , *13* , 134. https://doi.org/10.1186/1471-2105-13-134
72. Livak, K. J., & Schmittgen , T. D. (2001). Analysis of relative gene expression data using real-time Quantitative PCR and the 2(-Delta Delta C(T)) Method. *Methods (San Diego, Calif.)* , *25* (4), 402–408. https://doi.org/10.1006/meth.2001.1262

18. Attachments and Appendix

Annex I – Questionnaire of state of health SF-36V2

36-item Short Form Survey (SF-36)

Name: ____________________ **Date:** __________

The SF-36 asks for your perspective on your health. The information gathered from this survey will help monitor how you feel and how well you can do certain activities. Please answer the questions thoroughly and honestly.

1. In general, would you say your health is:

◯ 1. Excellent ◯ 2. Very good ◯ 3. Good ◯ Fair ◯ 5. Poor

2. Compared to one year ago, how would you rate your health in general now?

◯ 1. Much better now than one year ago
◯ 4. Somewhat worse now than one year ago
◯ 2. Somewhat better now than one year ago
◯ 5. Much worse than one year ago
◯ 3. About the same

The following items are about activities you might do during a typical day. Does your health now limit you in these activities? If so, how much?

3. Vigorous activities, such as running, lifting heavy objects, and participating in strenuous sports.

◯ 1. Yes, limited a lot ◯ 2. Yes, limited a little ◯ 3. No, not limited at all

4. Moderate activities, such as moving a table, pushing a vacuum cleaner, bowling, or playing golf.

◯ 1. Yes, limited a lot ◯ 2. Yes, limited a little ◯ 3. No, not limited at all

5. Lifting or carrying groceries.

◯ 1. Yes, limited a lot ◯ 2. Yes, limited a little ◯ 3. No, not limited at all

6. Climbing several flights of stairs.

◯ 1. Yes, limited a lot ◯ 2. Yes, limited a little ◯ 3. No, not limited at all

7. Climbing one flight of stairs.

◯ 1. Yes, limited a lot ◯ 2. Yes, limited a little ◯ 3. No, not limited at all

8. Bending, kneeling, or stooping.

◯ 1. Yes, limited a lot ◯ 2. Yes, limited a little ◯ 3. No, not limited at all

9. Walking more than a mile.

◯ 1. Yes, limited a lot ◯ 2. Yes, limited a little ◯ 3. No, not limited at all

10. Walking several blocks.

◯ 1. Yes, limited a lot ◯ 2. Yes, limited a little ◯ 3. No, not limited at all

11. Walking one block.

1. Yes, limited a lot
2. Yes, limited a little
3. No, not limited at all

12. Bathing or dressing yourself.

1. Yes, limited a lot
2. Yes, limited a little
3. No, not limited at all

During the past 4 weeks, have you had any of the following problems with your work or other regular daily activities as a result of your physical health?

13. Cut down the amount of time you spent on work or other activities.

1. Yes
2. No

14. Accomplished less than you would like.

1. Yes
2. No

15. Were limited in the kind of work or other activities.

1. Yes
2. No

16. Had difficulty performing the work or other activities (for example, it took extra effort).

1. Yes
2. No

During the past 4 weeks, have you had any of the following problems with your work or other regular daily activities as a result of any emotional problems (such as feeling depressed or anxious)?

17. Cut down the amount of time you spent on work or other activities.

1. Yes
2. No

18. Accomplished less than you would like.

1. Yes
2. No

19. Didn't do work or other activities as carefully as usual.

1. Yes
2. No

20. Emotional problems interfered with your normal social activities with family, friends, neighbors, or groups?

1. Not at all
2. Slightly
. Moderately
4. Severe
. Very severe

21. How much bodily pain have you had during the past 4 weeks?

1. None
2. Very mild
3. Mild
4. Moderate
5. Severe
6. Very severe

22. During the past 4 weeks, how much did pain interfere with your normal work (including both work outside the home and housework)?

1. Not at all
2. Slightly
. Moderately
4. Quite a bit
5. Extremely

These questions are about how you feel and how things have been with you during the last 4 weeks. For each question, please give the answer that comes closest to the way you have been feeling.

23. Did you feel full of pep?

- 1. All the time
- 2. Most of the time
- 3. A good bit of the time
- 4. Some of the time
- 5. A little bit of the time
- 6. None of the time

24. Have you been a very nervous person?

- 1. All the time
- 2. Most of the time
- 3. A good bit of the time
- 4. Some of the time
- 5. A little bit of the time
- 6. None of the time

25. Have you felt so down in the dumps that nothing could cheer you up?

- 1. All the time
- 2. Most of the time
- 3. A good bit of the time
- 4. Some of the time
- 5. A little bit of the time
- 6. None of the time

26. Have you felt calm and peaceful?

- 1. All the time
- 2. Most of the time
- 3. A good bit of the time
- 4. Some of the time
- 5. A little bit of the time
- 6. None of the time

27. Did you have a lot of energy?

- 1. All the time
- 2. Most of the time
- 3. A good bit of the time
- 4. Some of the time
- 5. A little bit of the time
- 6. None of the time

28. Have you felt downhearted and blue?

- 1. All the time
- 2. Most of the time
- 3. A good bit of the time
- 4. Some of the time
- 5. A little bit of the time
- 6. None of the time

29. Did you feel worn out?

- 1. All the time
- 2. Most of the time
- 3. A good bit of the time
- 4. Some of the time
- 5. A little bit of the time
- 6. None of the time

30. Have you been a happy person?

- 1. All the time
- 2. Most of the time
- 3. A good bit of the time
- 4. Some of the time
- 5. A little bit of the time
- 6. None of the time

31. Did you feel tired?

- 1. All the time
- 2. Most of the time
- 3. A good bit of the time
- 4. Some of the time
- 5. A little bit of the time
- 6. None of the time

32. During the past 4 weeks, how much of the time has your physical health or emotional problems interfered with your social activities (like visiting with friends, relatives, etc.)?

- ◯ 1. All the time
- ◯ 2. Most of the time
- ◯ 3. A good bit of the time
- ◯ 4. Some of the time
- ◯ 5. A little bit of the time
- ◯ 6. None of the time

How true or false is each of the following statements for you?

33. I seem to get sick a little easier than other people.

◯ 1. Definitely true ◯ 2. Mostly true ◯ 3. Don't know ◯ 4. Mostly false ◯ 5. Definitely false

34. I am as healthy as anybody I know.

◯ 1. Definitely true ◯ 2. Mostly true ◯ 3. Don't know ◯ 4. Mostly false ◯ 5. Definitely false

35. I expect my health to get worse

◯ 1. Definitely true ◯ 2. Mostly true ◯ 3. Don't know ◯ 4. Mostly false ◯ 5. Definitely false

36. My heart is excellent

◯ 1. Definitely true ◯ 2. Mostly true ◯ 3. Don't know ◯ 4. Mostly false ◯ 5. Definitely false

Total score:

Additional notes

Annex II - Scale Visual Analog (EVE) to assessment of level of pain

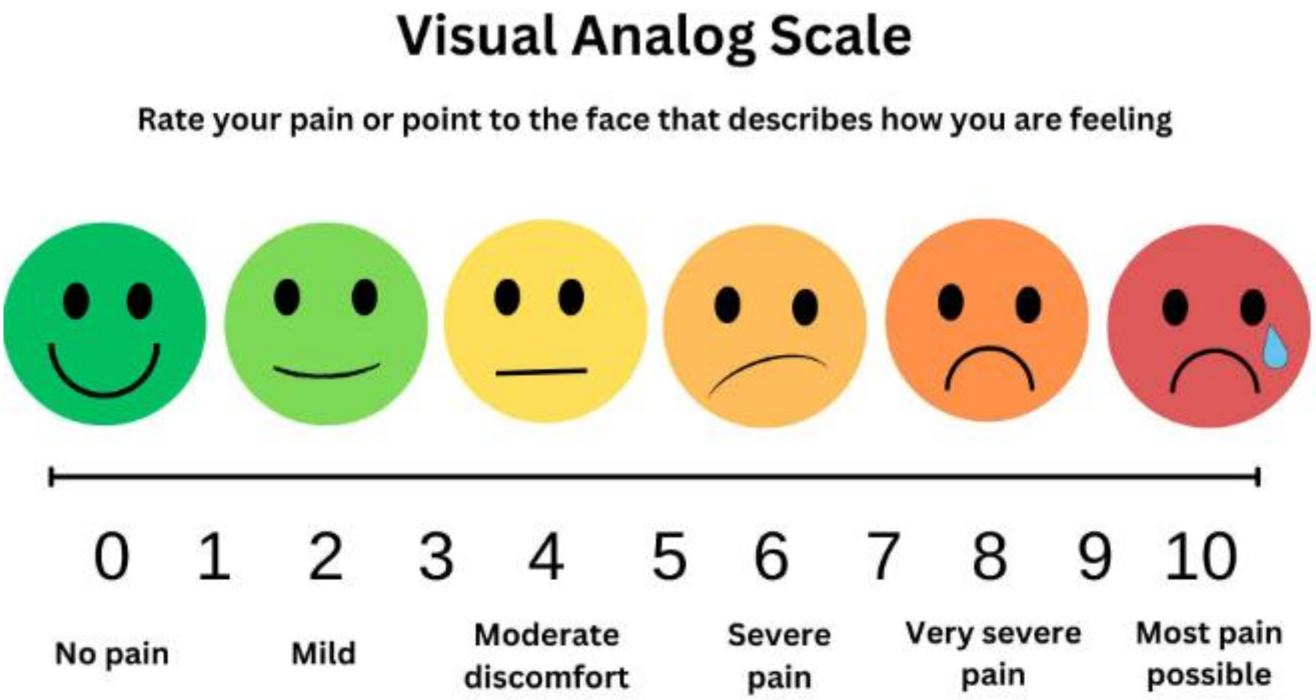

Annex III – Questionnaire EQ-5D

Under each heading, please tick the ONE box that best describes your health TODAY.

MOBILITY
- I have no problems in walking about ☐
- I have slight problems in walking about ☐
- I have moderate problems in walking about ☐
- I have severe problems in walking about ☐
- I am unable to walk about ☐

SELF-CARE
- I have no problems washing or dressing myself ☐
- I have slight problems washing or dressing myself ☐
- I have moderate problems washing or dressing myself ☐
- I have severe problems washing or dressing myself ☐
- I am unable to wash or dress myself ☐

USUAL ACTIVITIES (e.g., work, study, housework, family or leisure activities)
- I have no problems doing my usual activities ☐
- I have slight problems doing my usual activities ☐
- I have moderate problems doing my usual activities ☐
- I have severe problems doing my usual activities ☐
- I am unable to do my usual activities ☐

PAIN/DISCOMFORT
- I have no pain or discomfort ☐
- I have slight pain or discomfort ☐
- I have moderate pain or discomfort ☐
- I have severe pain or discomfort ☐
- I have extreme pain or discomfort ☐

ANXIETY/DEPRESSION
- I am not anxious or depressed ☐
- I am slightly anxious or depressed ☐
- I am moderately anxious or depressed ☐
- I am very anxious or depressed ☐
- I am extremely anxious or depressed ☐

1. We like to know how is your health today.

2. This scale is marked from 0 to 100.

3. 100 means the best health you can imagine.
0 means the worst health you can imagine.

4. Mark an X on the scale to indicate how is your health today.

5. Now, please note the number you marked on the scale in the box below.

Your Health Today = ☐

The best health you can imagine

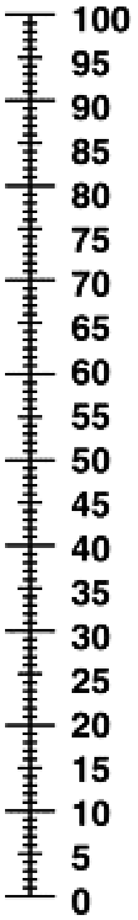

The worst health you can imagine

Appendix I - TCLE

TERM OF CONSENT FREE AND ENLIGHTENED (TCLE)
RESOLUTION 466/2012

I INVITE you to participate in the Research Project entitled " **CLINICAL STUDY RANDOMIZED, PROSPECTIVE, CONTROLLED, SINGLE-CENTER AND OPEN PHASE II TO TO ASSESS THE SECURITY AND THE EFFECTIVENESS FROM THE THERAPY WITH STEM CELLS MESENCHYMAL AUTOLOGOUS FOR ULCERS IN CARRIERS OF CRITICAL ISCHEMIA OF LOWER LIMBS – STEM-CELL STUDY I** ", which will be developed by me Prof. Dr. Matheus Bertanha, Vascular Surgeon, with the collaboration of Profs. Drs. Marcone Lima Sobreira, SurgeonVascular.

I am studying the disease peripheral arterial (DAP), responsible for the decrease of circulation blood in the lower limbs and causing their wounds that do not heal. So that I can have a result at this point I need to collect 20ml of your blood which will be used to perform the following exams laboratory (blood count full, urea, creatinine, sodium, potassium, glucose in fast, hemoglobin glycated, CPK, TGO, TGP, PCR, bilirubin, PTF, coagulogram, Acid Uric, Cholesterol total,HDL, LDL, Triglycerides, HIV type I and II, HTLV type I and II and Hepatitis B and C). This will be done in three moments during the study or more times if we consider it necessary, with the first collection being before any study procedure, to assess your general health conditions. The risk of collecting of blood it will be the minced meat from the needle and a little spot purple that will disappear good quickly.

You it will be included node study to to participate of form random of two possible groups of treatment and it will not be possible to change from one group to another. One of the groups will be the patients who will receive treatment only with Hydrogel dressings, and if you participate in this group, you will have a routine of appointments to attend and you must correctly follow the instructions that will be given more the front. From the same form, you may to participate of group that will be submitted to thetreatment with stem cells mesenchymal (CTM) what will be withdrawals of you same. Two procedures will have to be performed. In the first, a small surgery will be scheduled to collect a small fragment of skin and fat from your belly measuring 2cm in diameter, which will be performed under anesthesia local and without the need for hospital admission. This material will be taken to our laboratory and from there we will remove the CTM and feed them so that they increase in number. If you participate in this group, the The risks of this first procedure are small, such as: infection, non-healing of the wound, allergy to the anesthetic medication, among others. The second procedure will be performed three or four weeks after the collection of belly fat. Again, we will need some of your blood, around 50ml, as when you arrive for care, so we can prepare your cells. As soon as the cells are prepared, around 3 hours after the appointment, you will undergo a surgical procedure with local block or spinal anesthesia and some sedation for you no to feel pain, what it will be chosen for the anesthesiologist of form what it is the more safe to you. Afterof the surgical preparations, a good cleaning of the wound will be done, the injection of your MSCs by small punctures on the edges of the wound and a dressing containing MSC will also be placed on the wound. You will receive guidelines of care with that dressing. In this moment, you risks related will be: you risks anesthetics; allergies to medications, in addition to the possibility of the new technique not working. You must receive high yet in this same day.

I hereby inform you that the biological material collected from you, CTM obtained from the fatty tissue of your belly,will not be used in its entirety, as part of this material will be used for strict controls of the its quality and part will be stored at the Faculty of Medicine. This material will only be used in the duration of this research (2 years from the collection), strictly as explained and after the two years will be discarded.

I request also your consent to consult your medical record doctor to collect others information contained in queries made by you and data that may be relevant to this search

In addition, you must attend appointments that will be scheduled (7 days, 30 days, 60 days)days, 90 days and 120 days) for evaluation of dressings. During these consultations you will answer some questionnaires for the monitoring of your health, what will take about of 15 minutes of duration, in each query.

I inform also what, participating in any of the two groups, some others exams will be performed, such as vascular ultrasound with Doppler, Thermography of the skin using a camera special photographic, measurement of oxygen and carbon dioxide levels in the skin using a simple electrode, which will take about an hour and will be performed in 3 consultations. Your ulcer will be photographed only foruse in research or to demonstrate scientific results without you being identified. All these procedures do not require any preparation and should not expose you to any risk. additional.

Your benefit in participating in the research will be to have the chance to receive a new treatment with CTM taken from yourself that can help improve your circulation and increase your chances healing of your ulcer. If there is no benefit at this time and directly for you, this search will bring benefits to futures patients, after the collect and knowledge of the results of this search.

Please be aware that your participation in this study is voluntary and that even after you have given your consent, consent to participate in the research, you may withdraw it at any time, without any prejudice in continuity of your treatment.

This Term of Consent Free and Enlightened he was elaborated in 2 ways of equal content, the which 01via it will be delivered to the Madam) properly initialed, and the other via it will be archived and maintained by the researchers by one period of 5 years after the end from the search.

If you have any additional questions, you can contact the Research Ethics Committee. through of the phones (14) 3880-1608 or 3880-1609. He works of 2nd the 6th fair of the 8.00 to the 11.30 and of the 2:00 pm to 5:00 pm, at Chácara Butignolli s/nº in Rubião Júnior – Botucatu - Sao Paulo. The data from location of the researchers they are described below.

After have been healed all my doubts the respect of this study, I AGREE IN TO PARTICIPATE of form volunteer of this study, being aware what all you my data will be protected by the confidentiality to which the researchers have committed. I am aware that the results of thisstudy may to be published in magazines scientific, without, in the however, what my identity it is revealed.

Botucatu, / /20 .

______________________________ ______________________________

Participating Researcher from the Search

Name: Matheus Bertanha
Address: Av. Prof. Mário Rubens Guimarães Montenegro, s/n.
Neighborhood: UNESP - Campus of Botucatu CEP 18618687 -
Botucatu, SPPABX: (14) 3880-1001
Telephone: (14) 38801444
E-mail: matheusbertanha@fmb.unesp.br
